# Ultrafast Spectroscopy with an *Edge*—Basic Concepts and Tabletop Applications of Femtosecond X‑ray Transient Absorption Spectroscopy: A Tutorial Review

**DOI:** 10.1021/acsphyschemau.6c00024

**Published:** 2026-06-24

**Authors:** Caleb H. DeWitt, Aditi Bhattacherjee

**Affiliations:** Department of Chemistry, 4083 University of Iowa, Iowa City, Iowa 52242, United States

**Keywords:** ultrafast, electronic, transient, core, valence, X-ray, extreme ultraviolet, nonadiabatic, near-edge

## Abstract

Electronic absorption spectroscopy tools have long been part of the arsenal of molecular spectroscopy techniques to investigate the properties of valence electrons and chemical bonds. However, in recent years, this toolkit has rapidly expanded into the shallow and inner core orbitals, permitting a nuanced understanding of reaction dynamics in excited electronic states from the vantage points of distinct atomic sites. As these tools continue to be innovated, particularly in tabletop implementations, we provide an overview of how such versatile light pulses are (i) generated and (ii) applied in basic research laboratories, not only to answering long-standing questions in molecular spectroscopy but also to tackling emerging problems in energy conversion and quantum information science. In this tutorial review, we dive into a conceptual understanding of how femtosecond extreme-ultraviolet and soft X-ray pulses provide mechanistic understanding of ultrafast chemical dynamics through tracking wide regions of the molecular phase space with atomic-site fidelity. We further explain the nuts and bolts of a typical tabletop apparatus that control the parameters that determine the spectral and temporal resolution from the experimentalist’s standpoint. Finally, we circle back to spectroscopic selection rules from first-principles, connecting to several benchmark studies where femtosecond time-resolved X-ray absorption spectroscopy provides a cutting “edge” in the real-time mapping of nonadiabatic processes that operate far away from chemical equilibrium. Potential pitfalls and complementary methodologies that map other details of the evolution of electronic and nuclear structures on ultrafast time scales are discussed.

## Graphical abstract

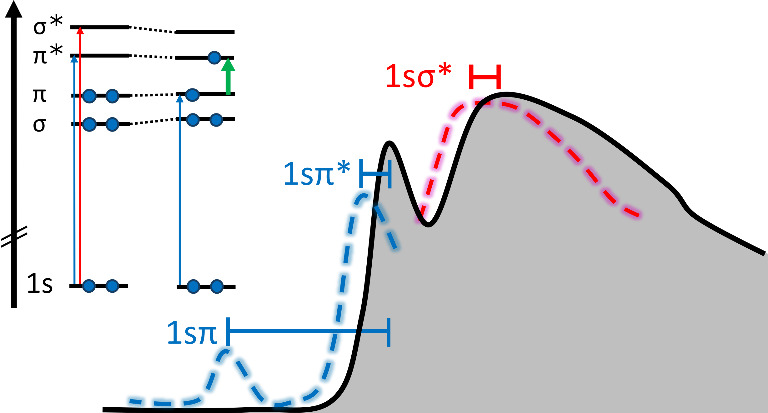

## 1 Introduction

The making and breaking of chemical bonds fundamentally ties the chemical sciences to a redistribution of the electron density within an isolated molecule or between two interacting molecules. Consequently, unimolecular and bimolecular reactions reveal rapid changes in the associated electronic structures. A gradual, noninstantaneous adjustment to the fast changing electronic structures on a dynamic potential energy surface triggers interatomic motions of the multinuclear (diatomic or polyatomic) framework. The separation in time scales for electronic and nuclear motion constitutes the basic premise of the Born–Oppenheimer (adiabatic) approximation.^1–3^ Within the adiabatic approximation, the molecular Schrödinger equation

Hψ=Eψ

can be separated into two decoupled equations for the wave functions and energies of electrons

$$H_{0}\phi_{n}\left(r,R\right)=E_{n}\phi_{n}\left(r,R\right)$$

and those for nuclei

$$\left(T_{nuc}+E_{n}\right)\chi_{n,i}\left(R\right)=E_{n,i}\chi_{n,i}\left(R\right)$$

where electronic energies, *E*_*n*_, are parametrically dependent on nuclear coordinates, *R* (clamped nuclei approximation). All relevant parameters and indices in the molecular Hamiltonian within the purview of the Born–Oppenheimer Approximation (Figure S1) are described in Table S1. With this separation of variables, the “true” molecular wave function (ψ) simplifies to a product state ψ = ϕ· χ of the wave functions for electrons (ϕ) and nuclei (χ).

Moreover, if the orbital (*L*) and spin angular momenta (*S*) for electron motion are taken into account and considered decoupled as a first approximation, which would be the case in the absence of spin–orbit (*L*·*S*) coupling, the “true” molecular wave function becomes

ψ=ϕ·χ·S

where ϕ itself is a product of “one-electron orbitals” (ϕ = ϕ_1_, ϕ_2_, ..., ϕ_*n*_) when ignoring electron–electron correlation. Although almost universally applicable to ground-state chemical reactions in equilibrium (Figure S1b), the Born–Oppenheimer approximation fails in many cases of excited state photochemistry (Figure S1c). These failures are observed in small or large ways when potential energy curves approach or cross each other, posing significant challenges for experimental and theoretical research methodologies.^4,5^ This tutorial review focuses on the particular aspects of an excited state reaction with inherent or potential failures of the Born–Oppenheimer approximation in which an experimentalist may benefit from using femtosecond transient X-ray absorption spectroscopy in the quest for mechanistic insight.

Figure 1a shows how the formation and breaking of chemical bonds occur in a representative bimolecular reaction of A + BC* → AB + C. A simple one-dimensional reaction coordinate is assumed where A collides with electronically excited BC to undergo an atom exchange reaction. Here, the use of * denotes electronic excitation. If A and BC are bonded together in a preactivated complex, the same reaction coordinate can be approximated to depict a unimolecular reaction, serving as a general reaction scheme. A quick back-of-the-envelope calculation shows why femtosecond pulses are needed for the characterization of over-the-barrier reactions within the equilibrium approximation.^6^ Consider that transition state theory formally predicts

$$k\left(T\right)=\frac{Q^{†}}{Q}\frac{k_{B}T}{h}e^{-E_{a}/k_{B}T}$$

for the temperature-dependent rate constant, *k*(*T*). It is determined by the exponential Boltzmann factor, *e* ^–E_*a*_/*k*_*B*_ *T*^, where *E*_*a*_, *k*_*B*_, and *T* are the activation energy, the Boltzmann constant, and the temperature, respectively. The ratio of partition functions, $\frac{Q^{†}}{Q}$ , for the transition state and reactants can be approximated as one since multiple bond breaking and formation is disallowed in transition state theory and reactants can cross the barrier only once. The factor $\frac{k_{B}T}{h}$ has the dimensions of inverse time and is intuitively a “frequency” to cross the barrier. The reciprocal of this crossing frequency is the “lifetime” of the transition state. Using *k*_*B*_ *T* ≈ 25 meV at room temperature (300 K) and Planck’s constant *h* = 6.626 × 10^–34^ m^2^kg s^–1^ (equivalently, 4.14 × 10^–15^ eV s), the lifetime of the transition state is approximately 10^–13^ s, which justifies the use of femtosecond (10^–15^ s) pulses to investigate reactive outcomes for the elucidation of mechanisms at the level of transition state theory.

**Figure 1:**
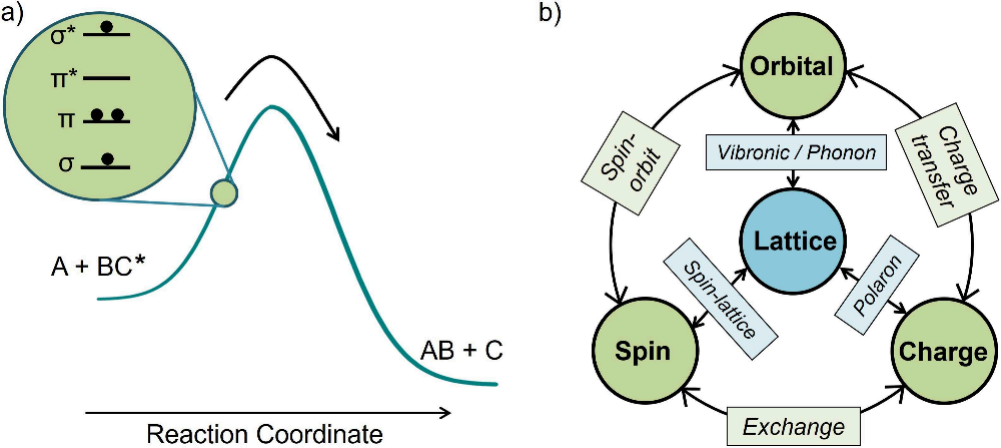
(a) Electronic structure changes in a representative A + BC* → AB + C chemical reaction are probed in time-resolved X-ray absorption spectroscopy (TRXAS) by exciting core electrons (not shown) into the frontier bonding and antibonding orbitals which are unoccupied or singly occupied. The molecular orbital diagram demonstrates a σ → σ* transition in a molecule with a π-bonding HOMO and a π*-antibonding LUMO. (b) Coupling mechanisms of electronic properties with nuclear positions in molecules and materials. X-ray absorption spectroscopy is directly sensitive to orbital and charge, and indirectly sensitive to electron spin through conservation of total (=orbital plus spin) angular momentum. Sensitivity to lattice motion is reflected through varying orbital overlap or partial charges on bonded atoms.

Generally, electronic or multiquanta vibrational excitation is necessary for bond dissociation, and in our representative example in Figure 1a, we focus on the former, shown as a one-electron transition from a bonding (example, σ) to an antibonding (example, σ*) orbital. The population of an antibonding σ* orbital is expected to be dissociative along the respective bond coordinate, for example, in the dissociation of the H_2_ molecule into H atoms for a σ → σ* excitation. Alternatively, the population of an antibonding π* orbital due to a π → π* transition is likely to produce a twist about the carbon–carbon double bond in ethylene or analogous unsaturated molecules, leading to transient pyramidalization, as an example.^7^ These ultrafast characteristics are so ubiquitous in bond dissociation and atom rearrangement reactions that they are colloquially referred to as loose bolt (bond elongation) and free rotor (twisting) effects in organic and inorganic photochemical reactions.^8–10^ Studying these effects, which underlie the majority of nonadiabatic reaction dynamics, is of great interest to identify mechanisms, mode selectivity, and vibrational coherences. Yet, unraveling the electronic and nuclear structural changes on femtosecond time scales is an outstanding challenge for many research methodologies with ultrafast temporal resolution.

Evidently, tracking electronic and nuclear motions in a chemical reaction involves the simultaneous measurement of electronic and nuclear degrees of freedom in molecular phase space. Electronic degrees encompass electronic charge, spin, and orbital occupancy, while nuclear degrees of freedom correspond to 3N translations, 2(3) rotations, and 3N – 5(6) vibrational degrees of freedom for (non)­linear molecules. These degrees of freedom are represented pictorially in Figure 1b. The outer core represents the electronic degrees, and the inner core is dubbed “lattice” as the collective nuclear degrees of freedom. The complexity escalates when each degree of freedom can further talk to each other via quantum-mechanical couplings dictated by the strength of the off-diagonal elements in the Hamiltonian interaction matrix. The experimentalist’s goal, at least at a glance, and greatly running the risk of oversimplification, is to study the electronic and nuclear degrees of freedom as several snapshots of the reaction measured in time. As we shall see later, the start of reaction clocking (time-zero) and the instrument response function play a key role in the depth of mechanistic understanding that can be attained. These experimental snapshots, when stitched together and analyzed *ex post facto*, should provide a clear mechanistic understanding of the electronic and nuclear degrees of freedom that characterize a reaction coordinate and reveal any significant quantum-mechanical couplings along these coordinates. The chemist’s approach could focus on frequency- or time-domain spectroscopies, and this choice is often a daunting task. Choosing a broadband probe is naturally advantageous, as we highlight in exemplary cases in this tutorial review, because it allows the increased accessibility of wider regions of the molecular phase space where out of equilibrium processes are both pervasive and predominant.

## 2 Potential Energy Surfaces and the “Optically Coupled” Region

The concept of molecular phase space helps greatly in the description and visualization of quantum states, transitions, and dynamics, especially in non-Born–Oppenheimer systems.^11,12^ Points in the phase space are described by a set of position and momentum coordinates (*r*, *p*) that, taken together, reflect a molecule’s microscopic state including its energy and location. As described earlier, the energy is distributed across the translational, rotational, and vibrational degrees of freedom of the molecule. Pioneering studies in femtochemistry by Zewail and co-workers can be used to rationalize the importance of the optically coupled region on potential energy surfaces within the molecular phase space (Figure 2), and how it critically informs the spectral (energy) and temporal (lifetime) characteristics that can be gleaned about the transition state region.^11^

**Figure 2:**
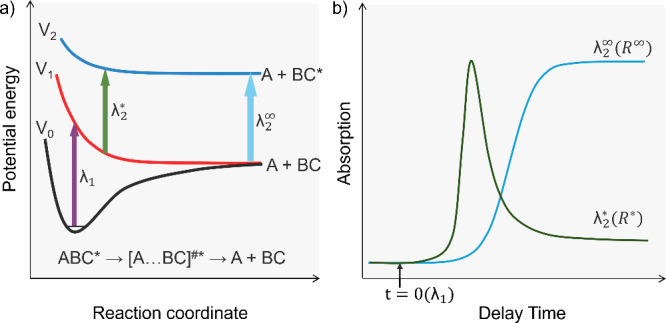
Optically coupled region in a pump–probe experiment. (a) Potential energy surface of a molecule in its ground state (V_0_) and excited states (V_1_ and V_2_). The pump and probe wavelengths are λ_1_ and λ_2_, and superscripts * and ^∞^ denote the transition state region and product asymptote, respectively. (b) A cartoon representation of the kinetic line-outs of reaction products (blue curve) and short-lived transients (green curve) in a pump–probe experiment as a function of femtosecond time delay. Modified with permission from ref 11 Copyright 1988 AAAS.

### Quantum State Preparation

The starting point of a chemical dynamics or pump–probe experiment is the preparation of a nonstationary quantum state. In ultrafast spectroscopy, this is usually described by a region in the phase space accessed by a vertical transition from the initial (ground) state (|*i*⟩ or |ψ_*i*_⟩) using a pump (excitation) pulse that is quantum-mechanically represented by a transition dipole operator, μ = *e*·*r*. Here, *e* and *r* represent the electronic charge and its position vector, respectively, for a one-electron transition. Thus, the transition dipole operator connects the initial and final states as ⟨ψ_*f*_|μ|ψ_*i*_⟩, and the corresponding squared quantity |μ_*fi*_|^2^ determines the transition strength. The region or subspace, |ψ_*f*_⟩(*t* = 0) at the ground-state equilibrium geometry is called the Franck–Condon region, where *t* = 0 denotes time zero (meaning, immediately after photoexcitation) and is represented by a vertical arrow in phase space (V_0_ to V_1_ at λ_1_, Figure 2a). The phase space footprint of the Franck–Condon region is governed by the initial distribution of ground-state equilibrium geometries projected on an excited-state surface such that *Δr* = 0 (vertical transition) and *Δp* = 0 (stationary phase approximation). The resonance condition or first-order transition probability is uniquely determined by the interaction of the ground state wave function |ψ_0_⟩ with the excitation light field within the framework of time-dependent perturbation theory. Note that the transition dipole is an induced, oscillating dipole that couples two stationary states and causes an electronic transition. Therefore, it represents the off-diagonal elements of the electric dipole operator when expanded in the quantum-mechanical basis of the stationary states corresponding to the unperturbed molecular Hamiltonian.

The Franck–Condon factors, |⟨χ_*f*_^*n*^|χ_*i*_^*m*^⟩|^2^, determine the spectral width of the electronic absorption, |*f*⟩ ← |*i*⟩, as the square of the vibrational overlap integral, where χ denote nuclear wave functions and *m* and *n* represent the quanta of vibrational excitation in the initial and final electronic states. The numeric values of *m* and *n* are therefore governed by the Boltzmann (equilibrium) and non-Boltzmann (nonequilibrium) vibrational population distributions in the ground and excited electronic states, respectively. The knobs available in ultrafast spectroscopy experiments to tune the quantum state preparation event are mainly through the photon energy, intensity, and spectral bandwidth of the femtosecond pump pulse. More advanced control parameters modulate the chirp or shape of the pump pulse, especially in the domain of quantum control experiments.^13,14^

### Quantum State Evolution

A superposition of several quantum mechanical wave functions (example, vibrational states in an excited electronic state, ∑_*n*_ *c*_*n*_ψ_*n*_ *e* ^–*iE*_*n*_ *t*/ℏ^) gives birth to a quantum mechanical wave packet on the excited state potential energy surface. Here, *c*_*n*_, ψ_*n*_, and *E*_*n*_ are the coefficients, wave functions, and energies of the *n* ^th^ vibronic level in the final electronic state, |*f*⟩. As the laser field couples the molecular eigenstates, the coefficients *c*_*n*_(*t*) become time-dependent. In the phase-space picture, the time evolution of the excited-state wave packet is determined by the gradient of the potential energy curve in the vicinity of the Franck–Condon region. The classical interpretation of the spread of the wave packet is that the “molecules fall along V_*f*_ with R → ∞ as t → ∞” (Figure 2a).^11^ The quantum-mechanical, nonadiabatic description requires an evaluation of the difference gradient and derivative coupling vectors (**g** = $\frac{\partial \left(E_{1}-E_{2}\right)}{\partial R}$ and **h** = $\left\langle \psi_{1}\right|\frac{\partial H}{\partial R}\left|\psi_{2}\right\rangle )$ , particularly in the vicinity of crossing points or conical intersections, where surface jumps are estimated by the Landau–Zener transition probability, exp $\left[\frac{-2\pi H_{12}^{2}}{\hbar v\frac{\partial \left(H_{11}-H_{22}\right)}{\partial R}}\right]$ , where *H*_12_ is the off-diagonal coupling element ⟨1|*H*|2⟩, $v=\frac{\mathrm{dR}}{\mathrm{dt}}$ is the velocity of the nuclear wave packet, *H*_11_ = *E*_1_ and *H*_22_ = *E*_2_ are the linear potential energies for the diabatic electronic states |1⟩ and |2⟩, and $\frac{\partial E}{\partial R}$ is the gradient of the potential energy surface. Detailed descriptions of the theoretical framework of adiabatic and nonadiabatic dynamics are found elsewhere.^15–17^

### Quantum State Detection

Reliable detection of the ensuing dynamics depends on whether or not a time-delayed probe pulse can be absorbed by the rapidly evolving transition-state geometries of the system. It is important to note that the transition state is traditionally associated with a barrier along an internuclear coordinate such as a saddle point, as shown in Figure 1a. However, a more advanced and encompassing viewpoint put forth by Zewail’s seminal femtochemistry work has extended this concept to dissociative reaction dynamics which have characteristic repulsive state surfaces. The chief argument is that several features of repulsive potential energy surfaces also conform to transition configurations; for example, all wave packet trajectories cross internuclear coordinates along this surface only once (as defined in transition state theory). The primary tool for observing these chemical transients as “configurations of no return” was initially coined femtosecond transition-state spectroscopy,^11,18–23^ and continues to be broadly applied in the context of photoinduced unimolecular reactions.^24–26^

Returning to Figure 2a, regions of the excited state surface and electronic/nuclear configurations that allow for the absorption of the probed photon are called the “optically coupled region”.^11^ Therefore, successful probing outcomes are determined by optical selection rules. To some extent, this restriction can be mitigated by a density of states argument - meaning that at progressively higher energies, there is always going to be a strong likelihood of finding a neighboring state to which the excited-state wave function can couple through the new transition dipole operator represented by the probe pulse. This likelihood stems from the higher electronic and vibrational density of states at higher energies, as can be examined in exact solutions of the hydrogen atom and quantum mechanical harmonic oscillator in text-book models.

Of the three preparation-evolution-detection steps described above, the quantum state preparation is the only one that is truly within the experimentalist’s control. The evolution and detection steps depend on our ability to clock reactions and detect the outcomes in a comprehensive and democratic way (Figure 2b). Suitable probing of the excited state derives from universal schemes that either overcome the selection rules entirely (example, through ionization to a vacuum state in time-resolved photoelectron spectroscopy, TRPES, that produces a free electron with all possible energies allowed, *i.e.*, no boundary conditions and therefore energy quantization) or offers easy tunability in the probe energy (example, core–electronic excitation into a vacant orbital according to an electric-dipole allowed transition in time-resolved X-ray absorption spectroscopy, TRXAS), or both (example, photoelectron spectroscopy involving the ionization of core electrons).^27,28^ Interestingly, theoretical work^7^ has shown that both the calculated TRXAS and TRPES contain information about the electronic dynamics in a molecule post excitation, but that this information seems to be encoded more clearly in TRXAS in the near-edge with sensitivity to specific atoms and orbitals.

## 3 Core or Valence Excitation as Choice of Probe: Why Care and What Difference does it Make?

The first direct experimental mappings of conical intersections, the most iconic of non-Born–Oppenheimer processes,^29,30^ utilized all-optical techniques.^31,32^ So why use core-level probes at all? This is a valid question whose answer depends on the objective of the experiment. In the following, we discuss the similarities and key differences between excitation of core and valence electrons as a probe. We frame this discussion from the point of view of Fermi’s golden rule (FGR), gradually working our way up to three important regions of the molecular phase space: the Franck–Condon region populated immediately after photoexcitation, the intermediate (transition state) region, and the asymptotic product states or vibrationally hot ground state due to the evolving/returning wave packet.

### Fermi’s Golden Rule Sets the Tone

Both sets of transitions, whether involving valence or core, are *exactly* governed by Fermi’s golden rule in oscillator strength. According to Fermi’s Golden Rule (FGR), the transition probability *W*_*fi*_ when a system in its initial state |ψ_*i*_⟩ absorbs incident photons with energy ℏω to reach the final state |ψ_*f*_⟩ is given by

$$W_{fi}=\frac{2\pi }{\hbar }\left|\left\langle \psi_{f}\right|T\right|\psi_{i}\rangle {|}^{2}\delta \left(E_{f}-E_{i}-\hbar \omega \right)$$

Here, *T* could be μ = *e*·*r* for the electric dipole transition, *x*_*i*_ *x*_*j*_ for the electric quadrupole transitions, μ_*m*_ for the magnetic dipole, *etc*. We consider the electric dipole term as the dominant light-matter interaction, such that the spectroscopic selection rules are governed by the parity of the transition dipole operator and of the initial and final electronic states. Therefore, for the electric dipole operator (with odd parity), the initial state ψ_*i*_ and the final state ψ_*f*_ must necessarily have opposite parity, *i.e., s* → *p* is allowed and *p* → *d* is allowed, but not *s* → *d*. The situation is reversed for electric quadrupole transitions, *i.e., s* → *d* *etc*. are now weakly allowed. Such dipole-forbidden transitions are routinely observed in the hard X-ray region, where the photon wavelength is comparable to atomic length-scales and the electric dipole approximation is no longer valid, which causes the selection rules to be relaxed greatly. This regime of X-ray spectroscopy is important in the >4.5 keV energies at free electron lasers. The K-edge transitions in titanium and iron metal–organic complexes present notable examples of dipole-forbidden but quadrupole-allowed 1s → 3d transitions in the pre-edge.^33,34^

Inspecting the transition probability from FGR, it is immediately apparent why molecular core-to-valence transitions bear a distinctive atomic edge. Since valence electrons found in frontier molecular orbitals are usually delocalized over bonded atoms, core–electron excitation provides a more localized probe of photoinduced charge transfer and electronic relaxation processes (Figure 3a). Therefore, the excitation of core electrons (represented by |⟨ψ_*f*_|*e*·*r*|1*s*⟩|^2^, |⟨ψ_*f*_ |*e*·*r*|2*p*⟩|^2^, *etc.*, in oscillator strength), unlike the excitation of valence electrons (represented by |⟨ψ_*LUMO*_|*e*·*r*|ψ_*HOMO*_⟩|^2^ *etc.*, in oscillator strength), uniquely encodes the atomic sites through the localized nature of the initial orbitals (1s, 2p, *etc.*) involved in the transition. This concept is illustrated schematically in Figure 3a–c. Thus, X-ray absorption spectroscopy is both element and orbital specific, and the ultrafast time resolution means that the changing electronic character and orbital mixing coefficients can be precisely tracked, offering a direct comparison to electronic structure theory. Note that Fermi’s golden rule dictates that transitions corresponding to the M edge show a higher oscillator strength in comparison to the L and K edges, as shown in Figure 3b–d. The former two edges also exhibit spin–orbit splitting (Figure 3c), which is more pronounced for the core-hole (≈eV) compared to the valence (tens to a few hundred meV).

**Figure 3:**
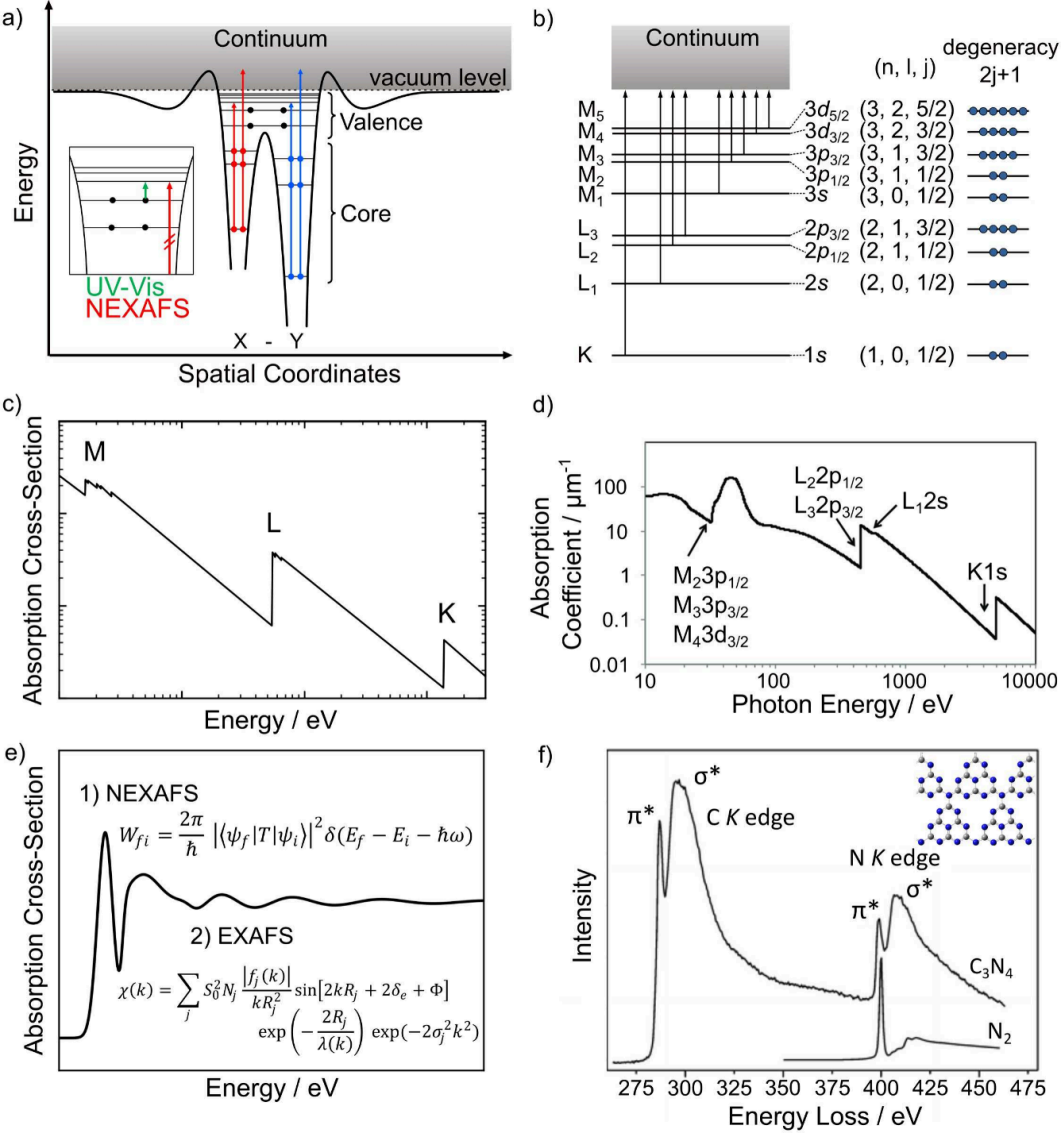
(a) Electronic transitions in a representative diatomic molecule (X-Y, Y is more electronegative than X) with a centrifugal barrier near the dissociation threshold. (b) Energy level diagram indicating the quantum states, quantum numbers, and quantum mechanical degeneracy (principal quantum number, *n*; orbital angular momentum quantum number, *l*; total angular momentum, *j* = *m* + *s*; and degeneracy, 2*j* + 1). (c,d) Schematic of X-ray absorption cross-section as a function of photon energy (c, for the K-, L-, and M-edges) with example of an experimentally measured X-ray absorption spectrum (d, of titanium) reproduced from ref 35. Available under a CC-BY 3.0 license. Copyright 2011 Seres et al. (e,f) Schematic and example of an atomic absorption edge in a molecular spectrum with the near-edge (e, NEXAFS) and extended-edge (e, EXAFS) absorption fine structures. The transition probability in NEXAFS is governed by Fermi’s Golden Rule (see text) and the EXAFS equation is described elsewhere (see the Supporting Information (SI)).^36,37^ An example of this edge structure is shown for a carbon nitride sample (f, at the carbon and nitrogen K-edges), adapted with permission from ref 38. Copyright 2008 Royal Society of Chemistry.

### Line Shape Broadening from a Few-Femtosecond Core-Hole Lifetime

Core-to-valence resonances usually undergo significant broadening as a result of the short, few-femtosecond lifetime of the core hole.^37^ Photoemission via Auger-Meitner decay is the primary mechanism for the filling up of core holes, and subfemtosecond lifetimes are also possible for core-hole states that decay via Coster-Krönig and super-Coster-Krönig processes.^39–41^ Peak widths can range from several hundred to thousands of milli-electron volts depending on the location of the edge. Atomic resonances present the sharpest line width because of the lack of any vibrational structure, whereas molecular resonances exhibit additional broadening from the underlying vibrational and rotational levels. Furthermore, in molecular spectra, the width of the σ* resonances is wider than that of π* due to a significant coupling with the ionization continuum at higher energies through molecular shape resonances. This mechanism is depicted in the schematic Figure 3e with a measured example in Figure 3f. Supersonic jet cooling can be used to determine the vibrational structure in core-to-valence resonances of molecular and free radical species. The core hole can decay by X-ray fluorescence emission in addition to Auger photoemission of electrons; when coupled with femtosecond optical pulses, these probing methodologies can provide complementary readouts of nonadiabatic dynamics and spin-crossover processes. Interested readers are referred to comprehensive review articles in these areas.^42–44^

### Spin–Orbit Splitting of the Core Hole for *p*- and *d*-Electron Transitions

With the exception of the K-edge (1s) and L_1_ (2s), M_1_ (3s), N_1_ (4s) edges for which *l* = 0, spin–orbit splitting (*L*·*S*) of the core hole is inevitable and must be taken into account in the analysis of X-ray absorption and transient absorption spectra. This feature is shown in Figure 3b–d. The spin–orbit coupling in the core hole can be few to tens of electron volts depending on the proximity to the atomic nucleus. In many-electron atoms, *L*_*z*_ = ∑_*i*_ *l*_*iz*_ is a good quantum number only in the absence of spin–orbit coupling. However, in the presence of spin–orbit coupling, *l*_*iz*_ does not commute with ∑_*i*_ *s*_*i*_ *l*_*i*_ (spin–orbit coupling operator). Consequently, *L*_*z*_ is no longer a good quantum number to denote a quantum state. The two types of angular momentum of an electron, spin and orbital angular momentum, can act as two sources of magnetic moment. We note that an electron has a spin quantum number *s* = 1/2 and that the spin magnetic quantum number is one of the two values *m*_*s*_ = ±1/2. Therefore

$$H_{SO}=-\mu \cdot B=\frac{1}{4\pi \epsilon_{0}}\frac{e^{2}}{m^{2}c^{2}r^{3}}S\cdot L$$

Physically, the spin–orbit Hamiltonian *H*_*SO*_ is attributed to the torque exerted on the magnetic dipole moment (μ) of the spinning electron due to the magnetic field (*B*) of the proton in the instantaneous rest frame of the electron. The operator *L*·*S* becomes^45^

$$L\cdot S=\frac{1}{2}\left(J^{2}-L^{2}-S^{2}\right)=\frac{\hbar^{2}}{2}\left[j\left(j+1\right)-l\left(l+1\right)-s\left(s+1\right)\right]$$

These are the eigenvalues of *L*·*S*.

$$E_{SO}=\left\langle H_{SO}\right\rangle =\frac{1}{8\pi \epsilon_{0}}\frac{e^{2}}{m^{2}c^{2}}\frac{\hbar^{2}/2\left[j\left(j+1\right)-l\left(l+1\right)-3/4\right]}{l\left(l+1/2\right)\left(l+1\right)n^{3}a^{3}}$$

which decreases rapidly as the principal quantum number *n* increases (see Tables S7–S9 for the various parameters). In this vein, spin–orbit splitting in the core hole (X-ray spectroscopy) is much higher relative to the valence orbitals (UV–vis spectroscopy). Furthermore, the spin–orbit splitting pattern follows an exact 1:2 or 2:3 (2*j* + 1, Figure 3b) quantum mechanical degeneracy expected for *p* and *d* sublevels with *l* = 1, 2, respectively, where *j* = *l* + *s*. In all cases of a core hole other than a 1s hole, multiplet effects are important to explain spectral shapes and intensity ratios precisely (Figure 4a).^46–48^ Specific examples of spin–orbit splitting in XUV spectroscopy applied to molecules are presented later with the XUV spectra of alkyl iodide (with spin–orbit splitting of the I atom in the core (4d) D_3/2_ and D_5/2_ states of 1.7 eV) and soft X-ray spectra of dimethyl disulfide (with spin–orbit splitting of the S atom 2p-core hole, *P*_1/2_ and P_3/2_ states, 1.6 eV).

**Figure 4:**
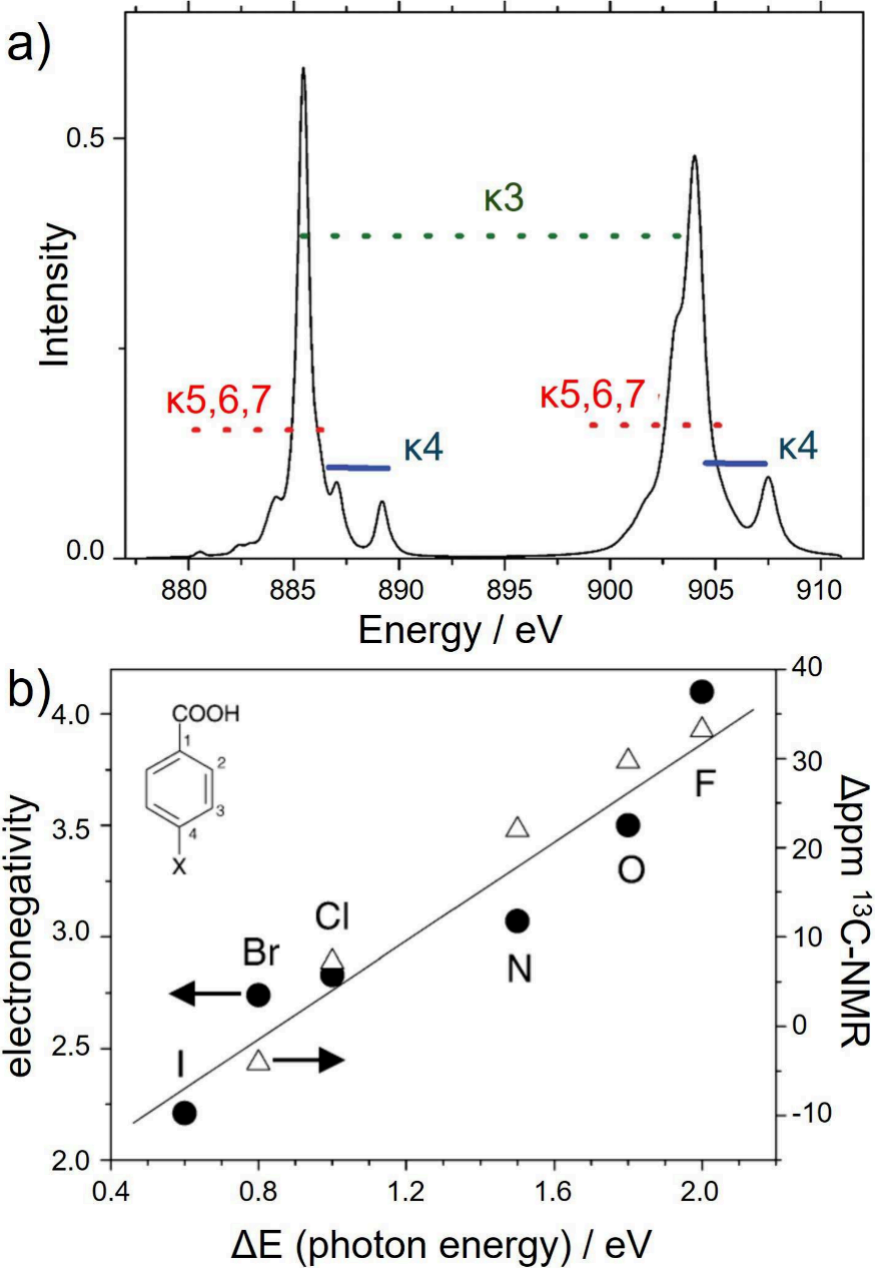
(a) Spin–orbit splitting (κ3), charge transfer (κ4) and multiplet effects (κ5,6,7) in cerium oxide measured at the Ce M edge. Reproduced with permission from ref 48. Copyright 2022 International Union of Crystallography. The effect of core-hole lifetime broadening is buried under the peaks and can be seen more clearly in Figure 1 of ref 48. (b) A linear correlation plot of X-ray chemical shift (Δ*E*, which measures the relative carbon NEXAFS peak positions of C-X, where X = F, N­(H), O­(H), Cl, Br, I, and the aromatic carbon C–H) and ^13^C nuclear magnetic resonance shift in a set of *para*-substituted benzoic acids with respect to electronegativity of the heteroatom X. Reprinted from ref 49. Copyright 2005, with permission from Elsevier.

### Chemical Shifts with Atomic Fidelity

Unlike valence electrons, core electrons are not shielded from the full Coulomb attraction of the nucleus. Hence, the binding energy of core electrons is highly sensitive to the charge state of the parent atom or ion. This effect is shown in Figure 3c,d, where within the HOMO–LUMO picture for molecular excited state processes (equivalently, valence and conduction bands for solids), the orbital characters of photoexcited electrons and holes can be tracked. Notwithstanding electron-correlation effects, the core-to-HOMO and core-to-LUMO transitions directly encode the HOMO–LUMO gap in molecular species and the electronic band gap in solids. Variations in the experimentally measured gap with the optical photon energy is an empirical measure of electron correlation, putting theoretical methods to the test. With attosecond resolution, fast processes, such as band gap renormalization, are successfully measured for semiconductors.^50^ In molecular systems, several factors affect chemical shifts, including charge transfer, electric fields, environmental charge density, and hybridization.^51^ As shown in Figure 4b, the difference in binding energies of the core electrons at the carbon K-edge (ΔE) strongly correlates with the electronegativity of the halogen atom in the para position for a series of *para*-substituted benzoic acids, a scaling that bears a close resemblance with the ^13^C-chemical shifts obtained from nuclear magnetic resonance spectroscopy.^49^ Therefore, as its most novel feature, ultrafast X-ray spectroscopy can be used to study a chemical reaction or process from the vantage points of distinct atomic sites.

### Indirect Sensitivity to Electron Spin Character

Inspecting the FGR equation, it is evident that X-ray spectroscopy is *not* sensitive to spin states. This lack of spin sensitivity stems from the fact that the electric dipole operator, μ = *e*·*r*, does not act on spin. Or put another way, electric-dipole transitions will always conserve the electron spin during photoexcitation such that it takes the same amount of photon energy to excite an electron from a fully filled lower energy level to an unoccupied upper energy level irrespective of whether the exciting electron is spin up or spin down. A small caveat here is that this statement holds specifically in the case of closed-shell molecules. In case of open-shell molecules such as oxygen, exchange splittings on the order of 3 eV are measured in the 1sσ* resonance which are absent in the main 1sπ* resonance at the oxygen K-edge (550 eV). This model text-book example of molecular oxygen^37^ is unpacked further in Figure S2, while noting here that for the closed-shell analogue of molecular nitrogen, a dipole-forbidden, ^3^Π ← ^1^Σ spin-flip transition can be observed at the nitrogen K-edge (410 eV) by using inner-shell electron energy loss spectroscopy (ISEELS) with low-energy electron impact excitation which bypasses the electric-dipole selection rule (unlike in the case of photoabsorption at the nitrogen K-edge).^52^

In polyatomic molecules with strong *L*·*S* coupling, X-ray absorption spectroscopy can *indirectly* probe spin configurations through its sensitivity to the orbital character. This indirect sensitivity stems from El Sayed’s formalism related to the conservation of total angular momentum (*J* = *L* + *S*), which requires a unit change in orbital angular momentum for a corresponding unit change in the spin angular momentum. Put another way, the torque generated from an electron jump, say from a *p*_*x*,*y*_ (*l* = 1; *m*_*l*_ = ± 1) to a *p*_*z*_ (*l* = 1; *m*_*l*_ = 0) orbital, can cause a spin flip from up (*s* = 1/2; *m*_*s*_ = +1/2) to down (*s* = 1/2; *m*_*s*_ = – 1/2) and vice versa. From a photochemical perspective, in many organic chromophores, an initially populated ^1^ ππ* state decays into a transiently populated ^3^ nπ* state upon spin crossover. We further discuss this interplay between spin and orbital angular momentum in Section 8 in the sub-2 ps intersystem crossing in acetylacetone,^53^ while noting here that El Sayed’s foundational rule has emerged as a design principle in the field of organic room-temperature phosphorescent materials.^54,55^ In many transition metal atoms bonded to organic chromophores, spin-crossover can be inferred experimentally from the energy gap between excited singlets and triplets, as the Pauli exclusion principle dictates that triplets are lower in energy than the corresponding singlets by the energy required for exchange stabilization.

The exchange interaction between a core hole and the net electron spin in the valence shell of an open-shell atom can be a sensitive reporter through spin-dependent X-ray excitation or X-ray emission spectroscopy.^56,57^ On tabletop setups employing high harmonic generation, extreme ultraviolet transient absorption spectroscopy has provided correct mechanistic understanding in transition metal complexes both in the gas phase^58,59^ and in the condensed phase,^60–62^ uncovering intersystem crossing that operates over extremely fast (100–200 fs) time scales. Notably, X-ray emission spectroscopy is a valuable probe of spin states, which has shown sensitivity of the emission peak energy and peak width to low-spin and high-spin states. This sensitivity has been demonstrated at a level where linear correlation plots can be constructed between the spin moment of the metal and its X-ray emission energy for Kα and Kβ lines.^56,63^ Most of these studies are performed at synchrotron facilities where the K-edge of the metal is accessible and is beyond the scope of tabletop measurements. However, when combined with ultrafast temporal resolution that can be targeted at free electron laser facilities, the critical role of intermediate spin states in governing ultrafast spin crossover mechanisms has been brought to the forefront.^64,65^

### Simultaneous Detection of the Franck–Condon Region, Intermediates and Final Products

Ultrafast optical pump and X-ray probe spectroscopy is devoid of coherent artifacts when the pump and probe pulses overlap in time; an inevitable circumstance in all optical pump–probe techniques. This key difference arises because the pump and probe wavelengths are nondegenerate and cannot “interfere”, so to speak. In contrast, cross-phase modulation effects greatly obfuscate the dynamics near a zero time delay between the pump and probe, *i.e.*, the Franck–Condon region. Therefore, depending on the duration of the pump pulse, ultrafast X-ray spectroscopy can map the Franck–Condon region specifically because the wavelength dependent time delay of the broadband probe pulse is not significant until one reaches tender X-rays.^66^ The use of a double crystal monochromator to select a narrow energy bandwidth at free electron laser facilities can cause variations in the path length as a function of the central X-ray energy and the Bragg angle of the crystals, which can be overcome by inline measurements and path-length compensation. Sequential double ionization of neon via two XUV photons can be utilized in an XUV pump–probe experiment at free electron lasers to quantify the extent of frequency chirp, including linear, quadratic and cubic components.^67^ Terahertz streaking and X-ray-induced phase shifts of a chirped optical probe pulse are also used to actively monitor the arrival time of X-ray pulses generated using free electron laser sources.^68,69^

Fortuitously, a wide spectral bandwidth of tabletop HHG probes ensures the simultaneous detection of chemical intermediates and final products in the same experiment. As an example, we present the case of the A-band photodissociation of allyl iodide (CH_2_=CH–CH_2_–I) at 266 nm.^24^ Here, the Franck–Condon region is accessed by a vertical transition from the ground-state molecular wave function (Figure 5a–d). The pump wavelength at 266 nm corresponds to an *n* → σ* transition where *n* is a nonbonding 5p electron on the iodine atom and σ* is antibonding with respect to the C–I bond. A differential absorption spectrum measured near the iodine N_4,5_ edge at 500 fs (Figure 5a) shows the ground-state resonances as four negative ΔOD peaks between 50 and 57 eV and the product I atom between 45 and 48 eV as two sharp peaks followed by a weak shoulder. Femtosecond clocking of the reaction from negative to positive time delays reveals spectroscopic transients labeled A, B, and C in Figure 5b. The sharp I atom features, denoted I and I*, correspond to ground-state and spin–orbit excited I atoms, respectively, which show comparable rise-times of 104 ± 20 fs and 74 ± 6 fs (Figure 5c) in the experiment. The transients, on the other hand, show an ultrafast rise and decay, corresponding to repulsive potential energy surfaces ^3^Q_0_ and ^1^Q_1_, where the quantum state preparation corresponds exclusively to the ^3^Q_0_ surface through a strong parallel transition at 266 nm. Thus, the appearance of I atoms in addition to I* is indicative of non-Born–Oppenheimer dynamics through a curve crossing behavior in the transition state region. In the one-electron picture, a straw-man’s model of the transition state region and curve crossing dynamics can be constructed from the measured peak energies of the 4d I atom resonances in the parent molecule and its product asymptote (Figure 5d). The A-band photodissociation has also been widely studied in other alkyl and acyl halides built on the same experimental scheme and further extended to the attosecond region.^25,58,59,70,71^

**Figure 5:**
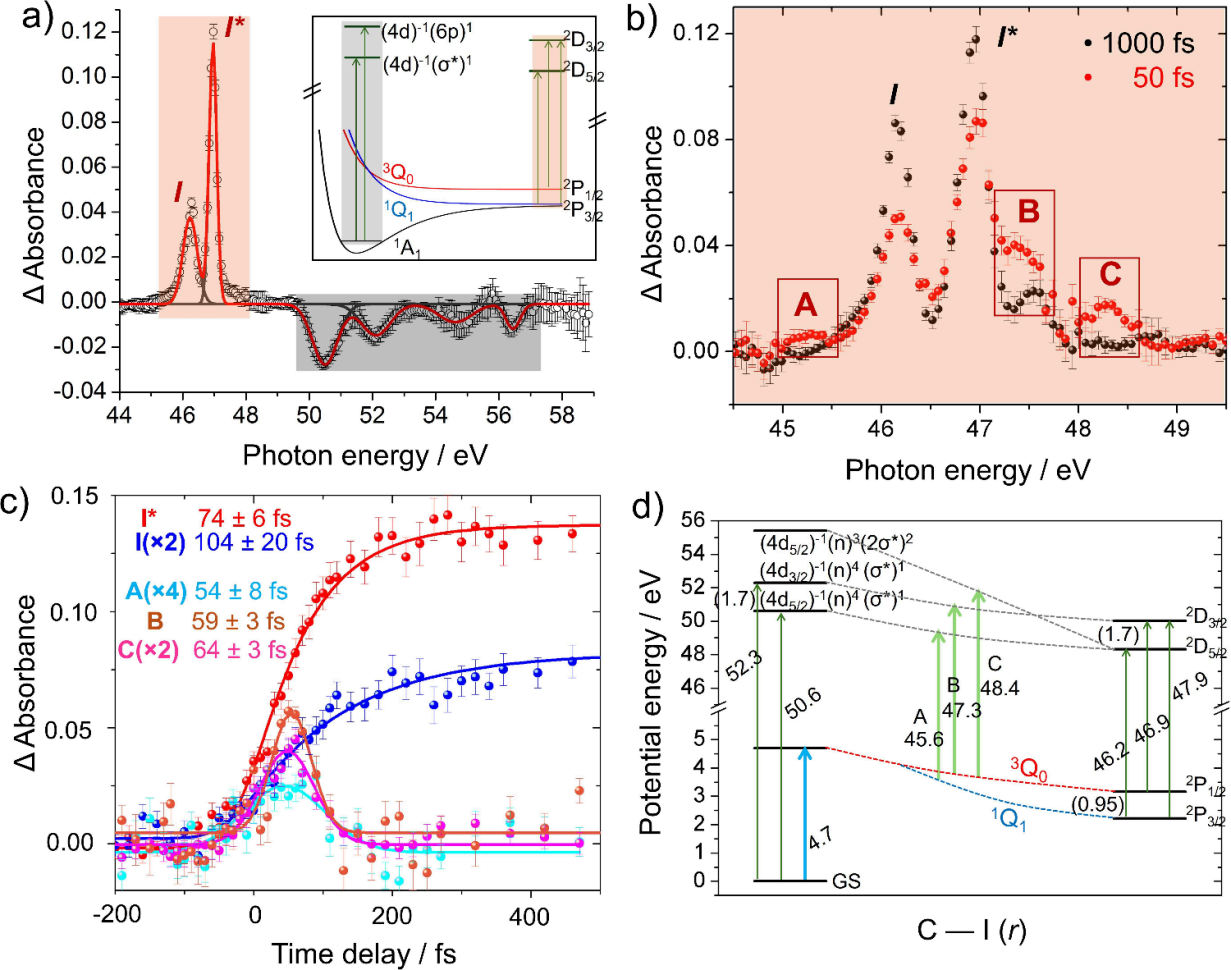
(a) Femtosecond XUV transient absorption spectrum of allyl iodide (CH_2_=CH–CH_2_–I). Inset shows a relevant potential energy schematic for A-band photodissociation and the experimental probing mechanism. The gray and peach shaded regions represent the molecular (C_3_H_5_I) and atomic (I) resonances in the reactant and product channels, respectively. (b) Transients labeled A, B, and C detected in the product I atom region (at 50 fs, red data points) in comparison to I atoms in the ground state (1000 fs, I) and spin–orbit excited state (1000 fs, I*). (c) Kinetics of I atom products and short-lived transients and (d) a straw-man one-electron model to understand the transition state region in the photoinduced reaction CH_2_=CH–CH_2_–I $\overset{266\,nm}{\to }$ CH_2_ = CH- *Ċ*H_2_ + I. Reprinted from ref 24 with the permission of AIP Publishing. Copyright 2016.

### Miscellaneous Examples of Broadband Atomic and Molecular Spectra

XUV and soft X-ray pulses can routinely measure core-to-LUMO, core-to-Rydberg, and above ionization thresholds in molecular spectroscopy. A synchrotron spectrum of CH_3_I (Figure 6) reported with quantitative oscillator strengths^72^ serves as an excellent starting point, as it reveals the ionization edge of methyl iodide both at the carbon K-edge and the iodine N_4,5_ edge for a direct comparison. Due to the technical difficulty and limited experimental accessibility of X-ray absorption spectroscopy, synchrotron data, when available, are the most valuable resource for planning the feasibility of tabletop experiments. In the figure shown, the oscillator strength is provided in megabarn on the right *y*-axis, which corresponds to a value of 10^–18^ cm^2^ molecule^–1^.

**Figure 6:**
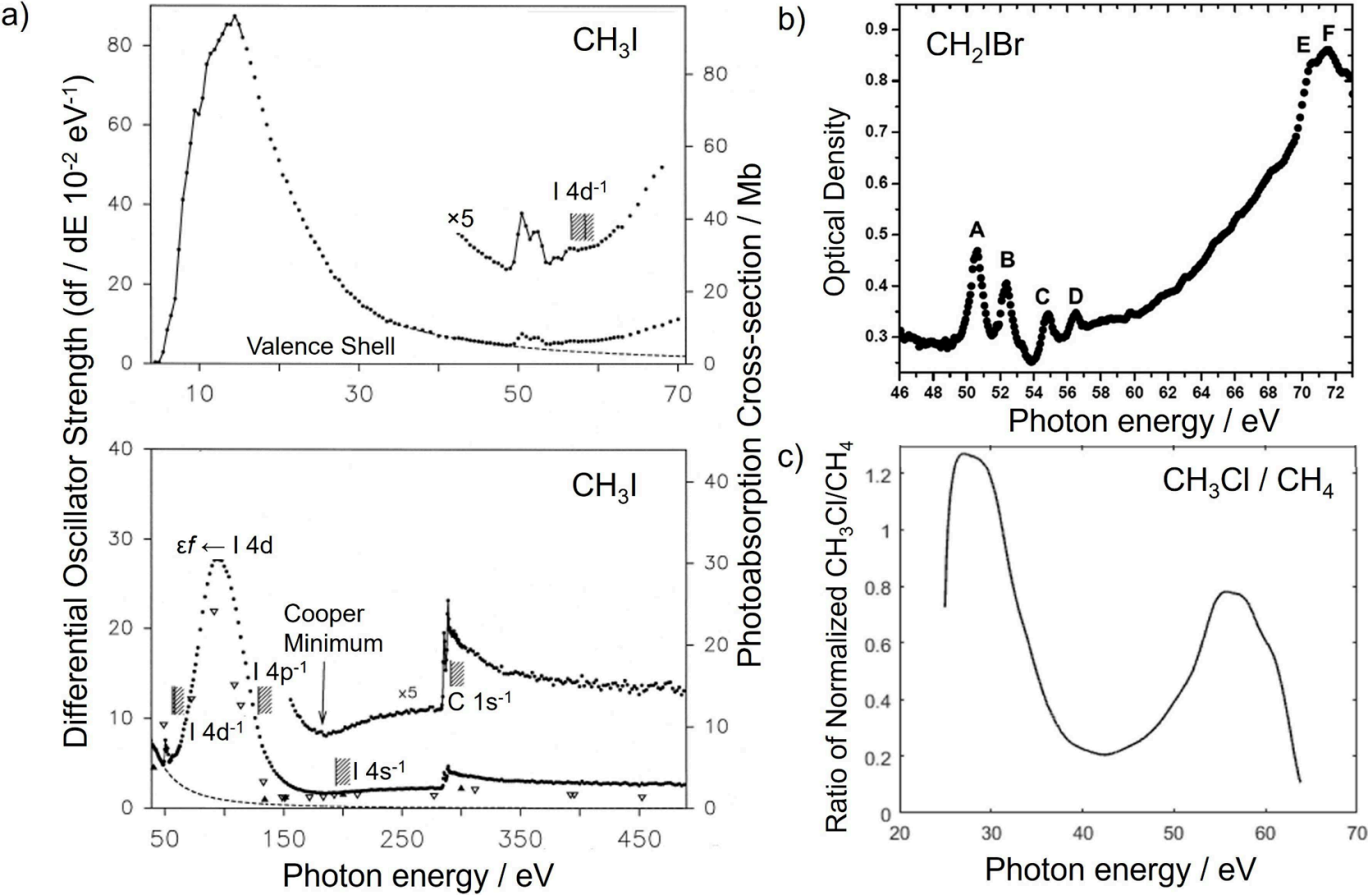
(a) Absolute photoabsorption oscillator strength for the XUV and soft X-ray spectrum of methyl iodide (CH_3_I) in the valence shell (5–40 eV), I­(4d, 50 eV), and C­(1s, 288 eV) inner shell regions. Mb stands for megabarn which is equivalent to 10^–18^ cm^2^ molecule^–1^. Reprinted from ref 72. Copyright 1998, with permission from Elsevier. (b) Static XUV absorption spectrum of CH_2_IBr measured at the iodine N_4,5_ and bromine M_4,5_ edges displays a giant resonance in the 4d­(I) core-level ionization. Reprinted from ref 73 with the permission of AIP Publishing. Copyright 2014. (c) Characterization of a molecular Cooper minimum taken as a ratio of the spectral intensity of methyl chloride (CH_3_Cl) relative to methane (CH_4_). Reproduced from ref 74. Available under a CC-BY 4.0 license. Copyright 2018 Scarborough et al.

The ionization threshold is the energy at which a bound electron in a system is set free from the confining Coulomb potential. Traditionally, mass analyzed threshold ionization (MATI) spectroscopy and zero kinetic energy electron (ZEKE) spectroscopy have been used to determine the onset of ionization by measuring the mass spectra of cations or kinetic energy of photoelectrons as the wavelength of the ionization laser is tuned.^75,76^ These methods provide information on ionization energy with a very high energy resolution (a few cm^–1^). Although at a much lower energy resolution (tens of a few hundred meV), X-ray absorption spectra reveal the onset of the ionization energy for core electrons at the respective atomic edges, and more accurate values may be obtained using X-ray photoelectron spectroscopy (XPS). Small volatile organic molecules such as dimethyl ether, methanol, acetone, formic acid, *etc.* exhibit chemical shifts in the ionization potential values at the carbon K-edge due to adjacency and bonding with electronegative oxygen atoms.^77^ When chemical shifts are considered in the framework of X-ray photoemission spectroscopy (XPS), where the final state is a one-electron vacuum state, photoejected electron intensities become directly proportional to the concentration of the chemical species to a first approximation. Moreover, according to the Thomas-Reiche-Kuhn sum rules, total integrated edge absorption (to bound and continuum states) is proportional to the number of orbital vacancies localized at the X-ray absorber atom and is reported to be well suited for investigating d-orbital vacancies at the L-edge.^78^ The sum rule dictates that the total (discrete and continuum) oscillator strength for an electronic transition in an atom or molecule is equal to the number of electrons (*n*) in that shell. In this context, K-shell (*n* = 2) integrated oscillator strengths in light atoms are often reported to be *less* than the theoretical value of 2 because dipole transitions to singly occupied valence orbitals are forbidden by the Pauli exclusion principle when the exciting core electron carries the same spin as the valence electron (Figure S2b).^79^

Before the ionization onset, Rydberg states are observed as a common feature in atoms and molecules as a series of electronically excited states of the neutral species that converge to the electronic ground state of the cation. These series of high principal quantum number (*n*) states are approximated by the well-known Rydberg formula.^80^ Spectroscopy of Rydberg states is a primary focus area in chemical physics and atomic, molecular, and optical physics. Figure 6a also shows a 4p^–1^ Cooper minimum and a 4*d* → *ϵf* giant resonance in methyl iodide, where the mere act of even discerning these features requires a wide spectral bandwidth. The Cooper minimum is a local minimum in the photoionization cross-section as a result of interference of the recombination dipole matrix element corresponding to two or more photoionization channels. Likewise, giant resonances are collective excitations in quantum systems that arise from strong electron correlation and many-body effects. These features are also reported in the XUV absorption spectra of CH_2_IBr and CH_3_Cl using tabletop HHG sources (Figure 6b,c).^73,74^

### Molecular Dynamics in the Water-Window Region

Tabletop soft X-ray sources have successfully measured molecular spectra and dynamics at the nitrogen K-edge (410 eV) and oxygen K-edge (550 eV) in the water-window region (284–550 eV) beyond the carbon K-edge (284 eV). The output wavelengths of an optical parametric amplifier pumped by a Ti:sapphire laser can nominally achieve HHG across the water window region from the carbon K-edge (*e.g.*, using the signal at 1400 nm) to the oxygen K-edge (*e.g.*, using the idler at 1800 nm).^81^ Strong-field ionization and fragmentation of molecular N_2_,^82^ nitric oxide,^83^ and photoinduced dissociation of NO_2_ ^84^ are reported at the nitrogen K-edge. These measurements have been possible in not only the gas phase but also condensed-phase systems, where liquid flat-jet technology has been instrumental in measuring solvents and solvated compounds.^85,86^ These significant technical developments have provided a wealth of information on non-Born–Oppenheimer processes such as excited state proton transfer reactions and coherence mechanisms that mediate electronic relaxation.^87,88^ Identification of exciton localization dynamics in organic photovoltaic devices has also been reported by tracking near-edge X-ray transitions.^81,89^ Time-resolved mappings of excited-state dynamics in molecules within solvent or extended lattice environments that contain carbon, nitrogen, or oxygen as the fundamental building blocks are powerful to answering central questions in physical organic chemistry, biochemistry, and materials science. Experimental breakthroughs in this emerging direction have recently been reviewed.^90^ Beyond spectroscopy, soft X-ray imaging with coherence tomography will bode well for visualizing site-specific reactivity.^91^

## 4 How are XUV Pulses Made?

Coincidentally, the same principle that forms the foundation of the Born–Oppenheimer approximation, *i.e.*, the sheer lightness of the electron (1/1837 of the mass of a proton), also allows a free electron to acquire very high kinetic energies when accelerated in an intense laser field. This feature makes high harmonic generation a unique probe as a tabletop source because its spectral coverage can span tens or sometimes over a hundred electron volts.^28,92^ By controlling the accelerating potential of the electron through the light field (center wavelength, intensity, *etc*.), it is possible to tune the XUV photon energies and pulse durations precisely. Figure 7 shows the central XUV photon energies and bandwidths produced in many tabletop implementations around the world. The floor of this 3D graphical rendering is indicative of the phase matching conditions employed, and the vertical axis indicates the X-ray cutoff energies. As shown, phase matching is a delicate balance of the laser driving wavelength and pressure of the generating gas medium, alongside the ionization potential (*I*_*P*_) of the gas medium, where cutoff energies increase according to Ar (*I*_*P*_ of 15.8 eV) < Ne (*I*_*P*_ of 21.6 eV) < He (*I*_*P*_ of 24.6 eV). Together, these experimental parameters determine the ionization rate, the ion-to-neutral ratio in the plasma for ideal phase matching, as well as the kinetic energy of tunnel ionized electrons toward cutoff energies. A cursory glance of Figure 7a shows that higher cutoff energies are achieved with increasing gas pressures of the generating medium and longer wavelengths of the driving laser field, and we describe the underlying causes for this type of scaling in this section.

**Figure 7:**
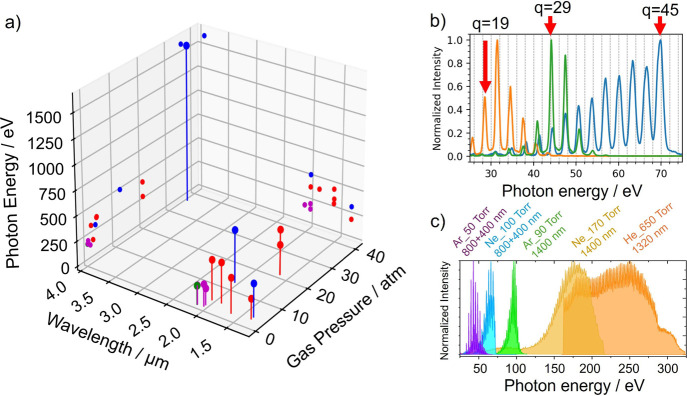
(a) X-ray energies produced on tabletop sources showing representative phase matching conditions (blue circles denote He, red circles denote Ne, magenta circles denote Ar, and green circles denote N_2_ as the generating medium). This panel captures the state of the art^93–98^ prior to the reported carbon K-edge harmonics shown in panel (c). A more up-to-date summary of tabletop, laser-based sources may be found in Table 1 in ref 99. (b) Typical high harmonic spectra on a tabletop source generated in the authors’ laboratory using a commercial Ti:sapphire laser (800 nm) and generating media of argon (25 Torr, orange trace, and 35–40 Torr, green trace) or neon (100 Torr, blue trace). Odd order harmonics of the fundamental are produced without a symmetry-breaking 400 nm field, where representative harmonic orders (q) are indicated with bold red arrows. c) Tunable XUV and soft X-ray high harmonic spectra on a tabletop HHG source. Reprinted with permission from ref 28. Copyright 2018 American Chemical Society.

### Technical Merit

First, one needs to rely on high harmonic generation because there is simply no option to buy tabletop X-ray lasers, *i.e.*, commercial short-wavelength lasers in the XUV and X-ray do not exist. This fundamental gap derives from the fact that when Einstein coefficients of spontaneous (*A*_21_) and stimulated (*B*_21_) emission are worked out explicitly, the rate of spontaneous emission far exceeds that of stimulated emission $\left(\frac{A_{21}}{B_{21}}=\frac{8\pi h\nu^{3}}{c^{3}}\right)$ with increasing frequency ν from infrared to deep- and extreme-ultraviolet. Therefore, high spontaneous emission rates and short excited-state lifetimes make tabletop X-ray lasers out of reach. One has to resort to relativistic electron motion manipulated by magnetic undulators only possible at X-ray free electron laser (FEL) facilities. These facilities have historically provided unprecedented photon energies and flux and are now embracing attosecond pulse durations and up to megahertz repetition rates.^100^

### Femtosecond Supercontinuum (or White-Light) Generation Is a Perturbative Process

We pivot to the topic of femtosecond supercontinuum or filamentation as it is central to most femtosecond laser laboratories, whether it be as versatile probe pulses in optical transient absorption experiments or as seed pulses for nonlinear parametric processes. These pulses can be subsequently up-converted or down-converted using parametric amplification and difference frequency generation schemes. The principles of femtosecond supercontinuum generation are discussed here briefly before working up our way to high harmonic generation, as this practical and pedagogical approach allows to set the distinction between χ^(*n*)^ and non-χ^(*n*)^ processes expected at the perturbative and nonperturbative limits of nonlinear spectroscopy, where χ^(*n*)^ is the *n* ^*th*^-order nonlinear optical susceptibility. Supercontinuum generation works on the principle of self-phase modulation for ultrashort pulses, a consequence of the optical Kerr effect, which describes how the refractive index of a material changes in response to an electric field.

Self-phase modulation is discussed in terms of the intensity-dependent refractive index $n=n_{0}+\frac{1}{2}n_{2}I\left(t\right)$ , where *n*_0_ and *n*_2_ represent the linear and nonlinear refractive indices, respectively, the latter dependent on intensity *I*(*t*). For a plane wave propagating through a medium, the electric field is denoted by *E*(*x*, *t*) = *E*_0_ *e* ^–*i*(*k*_0_ *x*–ω_0_ *t*)^, where *E*_0_ is the electric-field amplitude, $k_{0}=\frac{n\left(t\right)\omega_{0}}{c}$ is the wave vector, and ω_0_ denotes the angular frequency. However, an ultrashort laser pulse is not a monochromatic plane wave; therefore, its instantaneous frequency, ω­(*t*), is defined as the time derivative of the phase as

$$\omega \left(t\right)=\frac{\partial }{\partial t}\Phi \left(t\right)=\omega_{0}-\frac{x\omega_{0}}{c}\frac{\partial n}{\partial t}=\omega_{0}-\frac{n_{2}x\omega_{0}}{2c}\frac{\partial I\left(t\right)}{\partial t}$$

Thus, the instantaneous *change* in frequency

$$\delta \omega \left(t\right)=\omega \left(t\right)-\omega_{0}=-\frac{\omega_{0}n_{2}x}{2c}\frac{\partial I\left(t\right)}{\partial t}$$

is dependent on the negative derivative of the intensity profile in the time domain. For a Gaussian pulse, the rising edge (with a positive slope in intensity) will have a negative change in frequency (red-shift), and vice versa for the falling edge (Figure 8). Note that although the carrier frequency has an effect on the bandwidth of the generated supercontinuum,^101–103^ the central spectral-broadening mechanism is the intensity-dependent refractive index. The remaining question to consider is the origin of a nonlinear refractive index for the high electric field intensities that are routinely delivered by ultrashort pulses.

**Figure 8:**
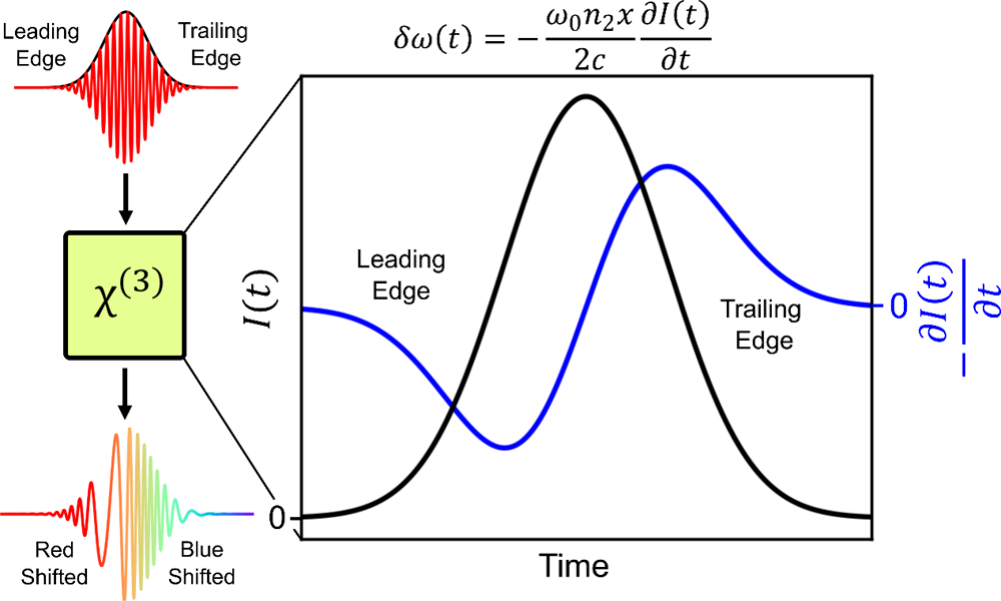
Schematic of white-light generation by self-phase modulation. When a near-IR pulse encounters a χ^(3)^ medium bearing an intensity-dependent refractive index, *n*_2_, it experiences an instantaneous frequency shift proportional to the negative time-derivative of the temporal intensity profile (right) of the Gaussian pulse envelope. This leads to a red shift in the leading edge (early time) of the pulse frequencies and a blue shift at the trailing edge (late time).

When a time-varying electric field interacts with a material, the problem can be cast within the framework of perturbation theory by considering the time-varying field as a small perturbation, *V*(*t*), that modifies the stationary ground-state Hamiltonian *H*_0_ as *H*(*t*) = *H*_0_ + *V*(*t*). Avoiding a laborious derivation,^104^ this perturbative approach allows the macroscopic polarization *P̃*(*t*) to be expressed as a Taylor series expansion in the electric field strength

$$\mathrm{P̃}\left(t\right)=\epsilon_{0}\left[\chi^{\left(1\right)}Ẽ\left(t\right)+\chi^{\left(2\right)}{Ẽ}^{2}\left(t\right)+\chi^{\left(3\right)}{Ẽ}^{3}\left(t\right)+...\right]$$

that describes the dipole moment per unit volume of the material and neatly separates the linear response, ϵ_0_χ^(1)^ *E*(ω), and the nonlinear responses, ϵ_0_χ^(*n*)^ *E* ^*n*^(ω) for *n* > 1. The use of the tilde symbol is for the complex-valued, time-dependent electric field as *Ẽ*(*t*) = *E*(ω)*e* ^–*iωt*^. The refractive index derives from the dielectric constant *n* ^2^ = ϵ_*r*_, which is defined by the effective susceptibility as ϵ_*r*_ ≡ 1 + χ_*eff*_. For light propagating through a nonlinear centrosymmetric material, the total polarization *P* ^*TOT*^(ω) ≡ ϵ_0_χ_*eff*_ *E*(ω), of the material is defined by

$$P^{TOT}\left(\omega \right)=\epsilon_{0}\chi^{\left(1\right)}E\left(\omega \right)+3\epsilon_{0}\chi^{\left(3\right)}|E\left(\omega \right){|}^{2}E\left(\omega \right)$$

such that the effective susceptibility is χ_*eff*_ = χ^(1)^ + 3χ^(3)^|*E*(ω)|^2^.^104^ Since the intensity is related to the square of the electric field (*I* = 2*n*_0_ϵ_0_ *c*|*E*(ω)|^2^), it follows that the refractive index, *n* ^2^ = ϵ_*r*_ ≡ 1 + χ^(1)^ + 3χ^(3)^|*E*(ω)|^2^, is dependent on the intensity of the light field. Altogether, this perturbative approach demonstrates the intensity dependence of refractive index, the practical application being that focusing a sufficiently intense near-IR beam in a χ^(3)^ medium generates a broad spectrum that stretches from the visible to mid-IR, *i.e.*, white-light generation (WLG).

### High Harmonic Generation Is a Nonperturbative Process

The practical components of high-harmonic generation are *similar* to white-light generation in that focusing a near-IR beam into a medium results in a broadband spectrum in the XUV to soft X-ray regime. However, the difference is made clear when an order of magnitude or higher intensity disparity is considered between white-light generation (10^12^ W/cm^2^) and high-harmonic generation (10^13^–10^15^ W/cm^2^). Keldysh theory, which describes the photoionization of an atom by frequencies well below the ionization potential, provides a metric for this comparison.^105^ The Keldysh parameter, γ, is a measure of the ratio of the tunneling time and the period of the driving frequency, $\gamma =\frac{t_{\mathrm{tu}}}{T_{0}}=\frac{\omega_{0}}{\omega_{\mathrm{tu}}}$ , where the tunneling frequency, $\omega_{\mathrm{tu}}=\frac{\mathrm{eE}}{\sqrt{2{\mathrm{mI}}_{p}}}$ .^106^ Therefore, the Keldysh parameter simplifies to

$$\gamma =\frac{\omega_{0}\sqrt{2{\mathrm{mI}}_{p}}}{\mathrm{eE}}=\sqrt{\frac{\omega_{0}^{2}2{\mathrm{mI}}_{p}}{e^{2}E^{2}}}=\sqrt{\frac{I_{p}}{2U_{p}}}$$

where *I*_*p*_ and *U*_*p*_ denote the ionization potential and ponderomotive energy, respectively. In the cases where the field strength, *E*, is sufficiently high such that the tunneling time is restricted to well below its oscillation period and γ < < 1, tunnel ionization dominates. In the opposite case, γ > > 1, multiphoton absorption is the dominant ionization mechanism. Figure 9a provides a look-up chart to orient the dominant processes in various intensity regimes of nonlinear optics.

**Figure 9:**
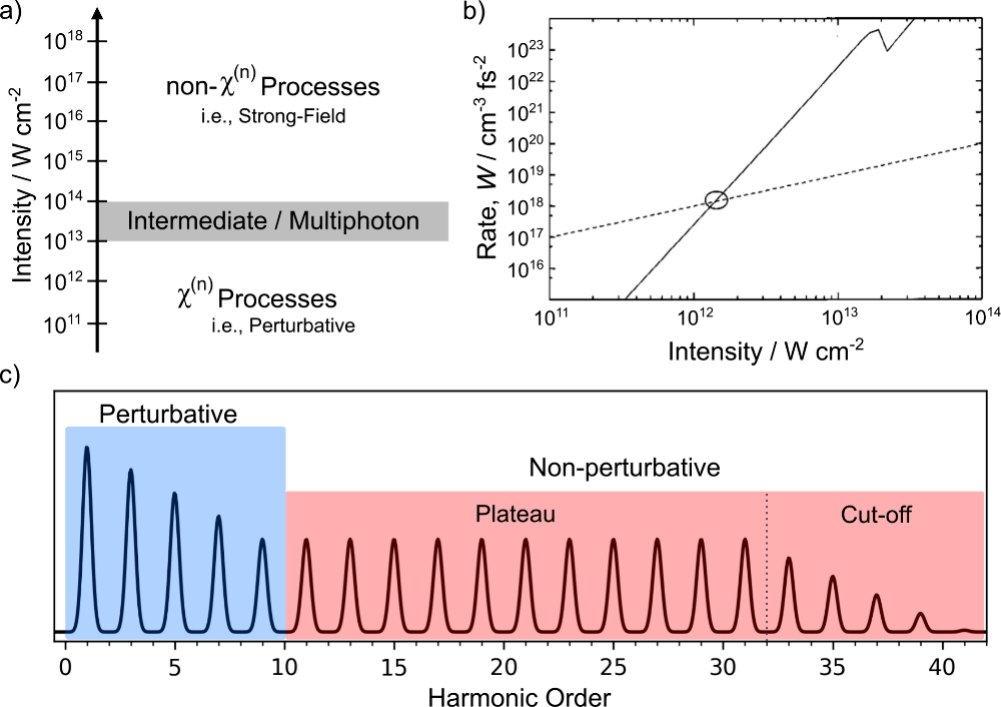
(a) Regimes of nonlinear optics showing the orders of magnitude for intensities needed to drive perturbative processes or strong-field processes. Note that the boundaries and interim regions of multiphoton ionization are not sharply defined. (b) A specific example of the intensity cutoff is shown, which plots the multiphoton excitation rate *W* versus the incident intensity in water (solid black line), reprinted with permission from ref 102. Copyright Optical Society of America. The dashed black line shows the multiphoton excitation rate against the intensity for which the Kerr index and plasma index cancel in water. The intersection of the lines denotes the intensity at which self-focusing stops in water. (c) Schematic of a high harmonic spectrum divided into the regions that can be described perturbatively or require a strong-field analysis. The nonperturbative region shows a long plateau, followed by a sharp cutoff.

According to Keldysh theory, multiphoton excitation begins to dominate at higher intensities and the dispersion due to free electrons counters the effects of self-focusing, causing an intensity cutoff.^102^ A specific example of this cutoff, shown in Figure 9b, graphs the intensity at which self-focusing stops in water due to plasma-induced self-defocusing effects. This high intensity, which causes ionization of the medium, is a basic requirement for high harmonic generation. Thus, the language of high harmonic generation is that of strong-field physics.

At the strong-field intensities required for HHG (10^13^ – 10^15^ W/cm^2^), the electric field strength approaches the Coulomb potential of an electron at the Bohr radius (*E*_*atom*_ ≈ 5 × 10^9^ V/cm, equivalent to an intensity of ∼10^16^ W/cm^2^), such that the interaction Hamiltonian (−μ·*E* in the dipole approximation) is no longer small compared to the ground state Hamiltonian *H*_0_ and cannot be treated as a weak perturbation.^107^ In this range, a perturbative treatment that considers an expansion of the transition dipoles will not converge. The general approach is provided in the strong-field approximation, where bound states are ignored, and the energy possibilities are considered for single electrons ejected into free space and propagating in the laser field.

### The Three-Step Model

The process of high harmonic generation is often discussed in the context of the “Three-step model”, which describes HHG *conceptually* using classical equations of motion for a single active electron in a laser field.^108^ A typical near-infrared ultrashort pulse shown in Figure 10 contains multiple optical cycles. In the semiclassical picture, the strong laser field ionizes the gas atom creating a free electron (step 1). As the electric field oscillates and the field changes sign, the potential reverses, which accelerates the electron back toward the ion. The free electron then recombines with the parent ion, releasing a strongly upconverted photon with energy that equals the ionization potential (*I*_*p*_) of the atom plus the instantaneous kinetic energy of the recombining electron.

**Figure 10:**
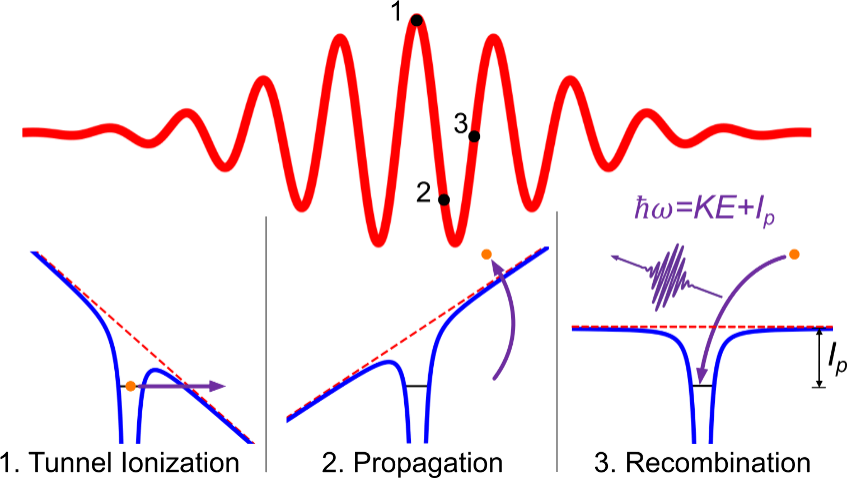
Schematic representation of the semiclassical Three-Step Model. The top trace represents a few-cycle optical pulse, the numbered markers (1–3) corresponding to the three stages below. In the first stage (panel 1), the superposition of the laser field potential (red dashed line) with the Coulomb potential (solid blue line) of the atom causes the latter to distort, forming a small energy barrier that allows direct or tunnel ionization of valence electrons depending on the extent of the distortion of the Coulomb potential. As the electric field amplitude changes sign in panel 2, the free electron is accelerated back toward the parent ion before recombining in panel 3 with instantaneous energy equal to the kinetic energy (*KE*) gained in the laser field and the ionization potential (*I*_*P*_) of the atom that leads to the emission of an X-ray photon (with energy ℏω = *KE* + *I*_*P*_).

The accelerating potential for the electron can be deterministically controlled as a superposition of the Coulomb potential of the generating atomic gas and the laser field that drives the HHG process. The probabilistic factors are tunnel ionization and electron recombination, although detailed theories of quantum mechanical scattering and Ammosov-Delone-Krainov (ADK) ionization permit educated estimates of the rates from the electron trajectory in the laser field.^109,110^ The important parameters to consider here are the electric field amplitude and its phase, and these are represented pictorially in Figures 10–11.

**Figure 11:**
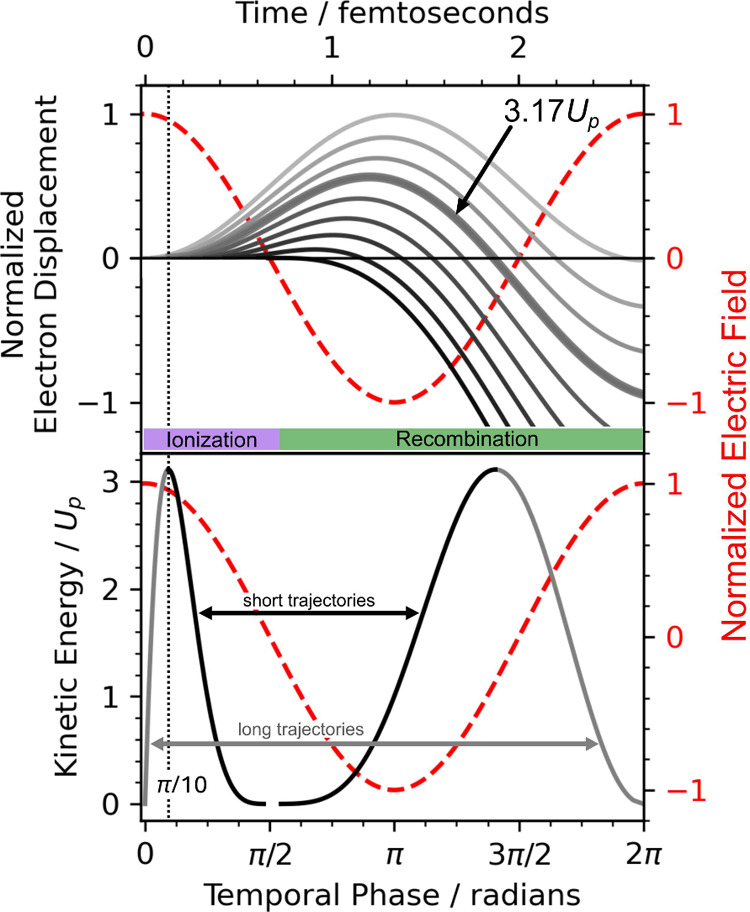
(Top panel) A plot of the electron displacement (black and gray traces) for free electrons that are ionized in the first quarter of an 800 nm optical cycle shown as a superimposed dashed red line. The electron displacement curves are color coded in grayscale to differentiate between the electrons ionized at early times (light gray) versus late times (dark gray). The bold gray line corresponds to the trajectory for the maximum electron kinetic energy (3.17 U_*P*_) acquired at a phase of 18° or π/10 radians. Shown directly below is the kinetic energy acquired by a free electron plotted against its ionization time (left curve at early times) or recombination time (right curve at late times). The traces are separated to distinguish between electrons that take a short (black) trajectory or a long (gray) trajectory in the laser field (superimposed dashed red line).

### Equations of Motion in the Three-Step Model

The equations of motion for the tunnel-ionized electron are worked out using classical electrodynamics, which enables starting researchers in the field to derive HHG as an ultrafast probe with multi-eV spectral bandwidth using pen and paper. The electrostatic force (*F*) experienced by a charged particle (carrying charge *q*) in an electric field (*E*) is defined as *F* = *q*·*E*. It follows that the acceleration of the particle (in one-dimension, for linearly polarized light) can be written as $\frac{d^{2}x}{{dt}^{2}}=\frac{\mathrm{qE}}{m}$ . For an electron in a monochromatic laser field *E* = *E*_0_ cos­(ω_0_ *t*) with frequency ω_0_ and amplitude *E*_0_, the velocity *v* and position *x* of the free-electron at time *t* can be solved for by integrating the equation of motion. At the initial time, *t*′, the electron will be defined with zero initial position (*x*) or velocity (*v*) (*e.g.*, the time at which the electron is freed by ionization)

$$\frac{d^{2}x}{dt^{2}}=-\frac{e}{m}E_{0}\;\cos\left(\omega_{0}t\right)$$

$$v\left(t\right)=\frac{dx}{dt}=\int_{t\prime }^{t}-\frac{e}{m}E_{0}\;\cos\left(\omega_{0}t\right)\;dt=-\frac{eE_{0}}{m\omega_{0}}\left[\sin\left(\omega_{0}t\right)-\sin\left(\omega_{0}t^{\prime }\right)\right]$$

$$x\left(t\right)=\int_{t\prime }^{t}v\left(t\right)\;dt=\frac{eE_{0}}{m\omega_{0}^{2}}\left[\left[\cos\left(\omega_{0}t\right)-\cos\left(\omega_{0}t^{\prime }\right)\right]+\omega_{0}\;\sin\left(\omega_{0}t^{\prime }\right)\left(t-t^{\prime }\right)\right]$$

The kinetic energy of a particle with velocity *v* and mass *m*, can be found by $\frac{1}{2}{\mathrm{mv}}^{2}$ . Therefore, the photon energy produced upon recombination will be

$$\hbar \omega =I_{p}+\frac{1}{2}m{\left[-\frac{eE_{0}}{m\omega_{0}}\left[\sin\left(\omega_{0}t\right)-\sin\left(\omega_{0}t^{\prime }\right)\right]\right]}^{2}$$

To solve for the generated photon energies, the recombination time (*t*) must be found for a given ionization time (*t*′) by determining when the electron position *x*(*t*) returns to zero, as shown graphically in Figure 11. The bottom panel of the figure plots the allowed kinetic energies for given ionization trajectories in a single optical cycle of an 800 nm monochromatic field, approximately 2.67 fs in duration. The kinetic energy is found relative to the ponderomotive energy $U_{p}=\frac{{\left({\mathrm{eE}}_{0}\right)}^{2}}{4m\omega_{0}^{2}}$ , which describes the cycle-averaged quiver energy that an electron acquires in an oscillating electric field. A major factor to consider here is the carrier frequency underlying the pulse envelope, which the reader will recall is not required for the description of femtosecond supercontinuum generation.

The semiclassical three-step model provides a theoretical maximum energy, or cutoff, for the XUV photons. At a temporal phase of 0.1π radians, the optimal electron trajectory is achieved with a final kinetic energy of 3.17*U*_*p*_, producing a photon with energy

$$\hbar \omega_{\max}=I_{p}+3.17\frac{{\left({\mathrm{eE}}_{0}\right)}^{2}}{4m\omega_{0}^{2}}.$$

Conversely, by converting the field amplitude to intensity,

$$\hbar \omega_{\max}=I_{p}+3.17\frac{e^{2}I}{2\epsilon_{0}\mathrm{cm}\omega_{0}^{2}},$$

one also obtains

$$\hbar \omega_{\max}=I_{p}+3.17\frac{e^{2}}{8\pi^{2}\epsilon_{0}c^{3}m}I\lambda^{2}=E_{\max},$$

where ϵ_0_ is the vacuum permittivity constant, *c* is the speed of light, and *e* and *m* are the charge and mass of an electron. Multiplying all universal constants to provide an equation in terms of intensity (in *W*/cm^2^) and wavelength (in nm) as the only variables, the ponderomotive energy can be found as *U*_*p*_ = 9.336 × 10^–20^(eV W^–1^ cm^2^ nm^–2^) × *I* (W cm^–2^) × λ^2^ (nm^2^). As an example, the maximum achievable energy for a 1.5 mJ driving pulse at 800 nm and 40 fs pulse duration when focused to a beam waist of 70 μm (*I* ∼ 2.8 × 10^14^ W/cm^2^) in argon (*I*_*p*_ = 15.8 eV) is 61 eV, approximately.

From the above expression (*E*_*max*_ ∝ *Iλ* ^2^), it follows that the energy cutoff scales linearly with the intensity and quadratically with the driving wavelength. Figure 7a will remind the reader that the phase-matching conditions explored to extend the cutoff energy intentionally utilized longer wavelength driving sources that provide more kinetic energy to the returning electrons. A natural consequence of longer wavelength driving lasers is a decreased recombination efficiency. The longer traversal time of the electron allows the electron wave packet to spread in time and space (quantum diffusion), leading to an inverse scaling of the recombination efficiency (η) with the wavelength, η­(λ) ∝ λ^–5^ – λ^–6^.^111–113^ To a certain extent, a higher cutoff frequency can be achieved by increasing the laser intensity. However, as the ionization rate increases with intensity, it follows that intense fields past the saturation threshold will start to deplete the ground-state population at nonoptimal phases for recombination, thereby decreasing the overall efficiency.^114^ The macroscopic conversion efficiency is dictated by the phase matching of the driving frequency with the produced XUV pulses, and is discussed in more detail later. Nonetheless, the semiclassical approach can provide a straightforward understanding of the achievable photon energies and even provides some indication of the temporal chirp of the produced pulses (as the energies produced are dependent on the phase of their return and the type of electron trajectories, short and long).^107,115^ Full quantum mechanical modeling of high harmonic generation has also been developed (the so-called Lewenstein model) which casts the semiclassical picture in a quantum theory, solving the time-dependent Schrödinger equation in the length gauge.^116^

### Periodicity in the Harmonic Spectrum

ADK (Ammosov-Delone-Krainov) theory dictates that the probability of ionization is maximum when the electric field strength is at its highest, dropping off steeply as a sharp exponential decay everywhere else. In other words, ionization events (and thus recombination/emission events) occur every half optical cycle. The resulting “train” of pulses produced will interfere to construct the familiar spectrum of sharp, discrete high harmonics (Figure 9c). As the spacing (repetition rate of the pulses) is equivalent to half an optical period (*T*_0_/2), it follows that the spacing in the frequency domain is twice the frequency of the fundamental laser source (2ω_0_). As even functions of the induced polarization cancel out in centrosymmetric media (*e.g.*, atoms), only odd-order harmonics are produced.^117,118^

Consider the electric field (*E*_ω_*q*__) of an arbitrary XUV frequency, ω_*q*_, of the *q* ^th^-order harmonic, summed over many half-optical periods (*n*) that change sign with every half-optical period as (−1)^*n*^

$$E_{\omega_{q}}=\underset{n}{\sum }A_{\omega_{q}}{\left(-1\right)}^{n}\;\exp\left[-i\left(\omega_{q}\left(t+\frac{nT_{0}}{2}\right)+\phi_{q}\right)\right]$$

$$E_{\omega_{q}}=A_{\omega_{q}}\;\exp\left[-i\left(\omega_{q}t+\phi_{q}\right)\right]\underset{n}{\sum }\exp\left[-i\left(\frac{\omega_{q}nT_{0}}{2}-n\pi \right)\right]$$

The amplitude is defined by the complex sum $\underset{n}{\sum }\exp\left[-i\left(\frac{\omega_{q}{\mathrm{nT}}_{0}}{2}-n\pi \right)\right]$ , such that when many half-optical periods (*n* > > 1) are summed, the geometric sum rule simplifies it to

$$\underset{n}{\sum }\exp\left[-i\left(\omega_{q}nT_{0}/2-n\pi \right)\right]=\frac{1-\exp\left[-\mathrm{ni}\left(\omega_{q}T_{0}/2-\pi \right)\right]}{1-\exp\left[-i\left(\omega_{q}T_{0}/2-\pi \right)\right]}$$

It follows that the amplitude is large when 1 – exp­[−*i*(ω_*q*_ *T*_0_/2 – π)] → 0, which is the case when ω_*q*_ = (2*n* + 1)­ω_0_. In other words, a monochromatic pulse with multiple optical cycles that result in ionization events will only produce energies that are *odd* harmonics of the driving frequency.^119^ This proof of principle can be demonstrated with a simple fast Fourier transform of a simulated train of pulses (Figure 12, top panels). As shown, a train of pulses that are ∼700 attoseconds in duration with a central frequency equal to the argon ionization potential (15.7 eV) and repeated at half the optical cycle of an 800 nm wave yields sharp frequency components separated by double the fundamental frequency of 800 nm (3.10 eV), with the strongest harmonic appearing closest to the central frequency chosen for the pulse (H9 = 13.95 eV). We note that this example does not produce an accurate energy range for what is expected in argon (30–55 eV) but is meant to provide an intuitive picture for the periodic spacing of harmonics.

**Figure 12:**
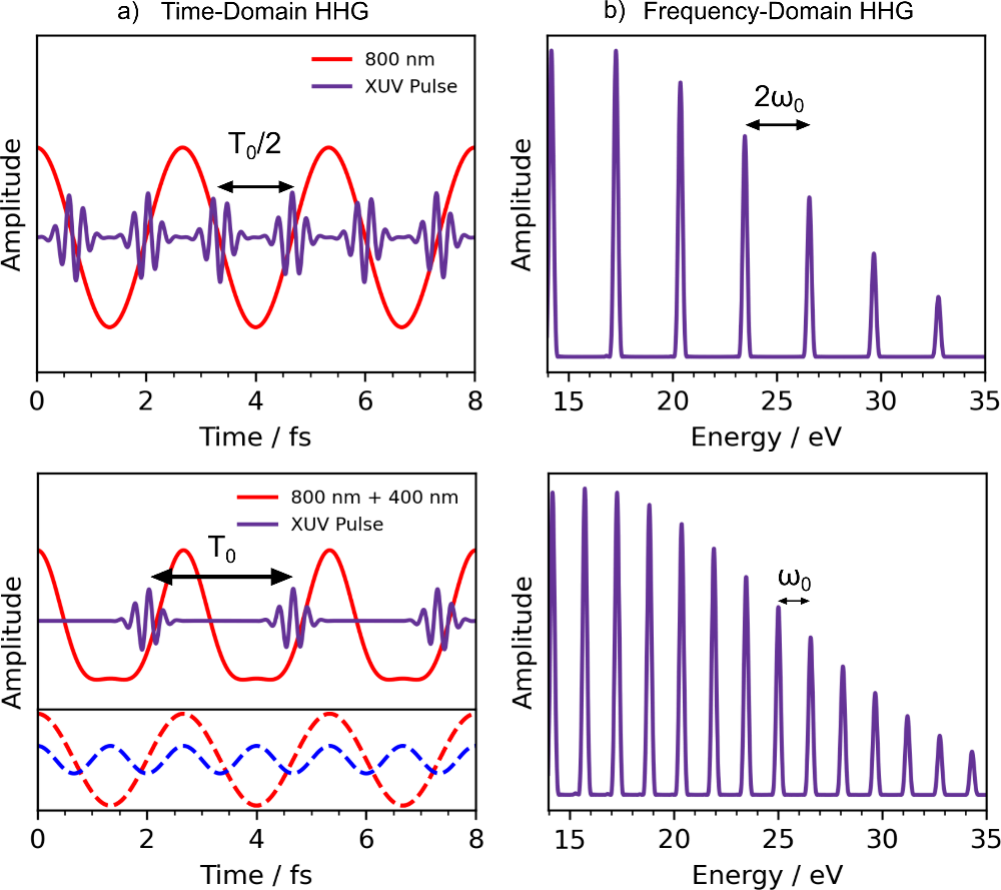
(a) A simulated train of attosecond pulses (blue trace) with 1/*e* ^2^ width of 700 as and central frequency equal to the ionization potential of argon (15.8 eV). These pulses repeat with half the period (*T*_0_/2) of an 800 nm laser field (red trace). (b) The fast Fourier transform (FFT) of the attosecond pulse train shows the extracted frequencies are separated by 2ω_0_. The lower two panels correspond to the same analysis but in the case where the attosecond pulses only appear with a periodicity of a full optical cycle of the fundamental field (*T*_0_). The red trace is an example of the broken symmetry that results when the fundamental is overlapped with a small amplitude (≈0.3) of its second harmonic (deconstructed in the bottom panel). In this case, the FFT yields spacing in the frequency domain equal to the frequency of the fundamental field, ω_0_.

### HHG in Two-Color Laser Fields

The discrete spacing of harmonics in the frequency domain produces alternating peaks and valleys which translates into a lower signal-to-noise ratio (SNR) at the valleys. This variation in SNR is especially an issue for operating in the XUV range with femtosecond pulses, driven by an 800 nm IR laser that produces harmonics spaced by 3 eV. As the 2ω_0_ spacing is determined by the *T*_0_/2 repetition rate of the returning electron wave packets, it follows that reducing the frequency of ionization events should also reduce the spacing between harmonics. Therefore, a common practice to fill the low-valley regions in a high harmonic spectrum is to overlap a small intensity of the second harmonic of the IR laser in essence to “break the symmetry” of the driving field.^120–122^ Since the ADK rate is strongly dependent on the amplitude of the field, the effect of introducing a small heterodyned, (10% amplitude or 1% intensity, Figure 13) second-harmonic field can cause a difference in the ionization rate of up to 3 orders of magnitude! This method effectively reduces the ionization frequency to integer multiples of *T*_0_, thereby decreasing the spacing between harmonics from 2ω_0_ to ω_0_. Such a heterodyne effect can be demonstrated again with a simple fast Fourier transform in Figure 12 (bottom panels) which considers the same train of attosecond pulses as before (top panel of the same figure), but with exactly half the repetition rate.

**Figure 13:**
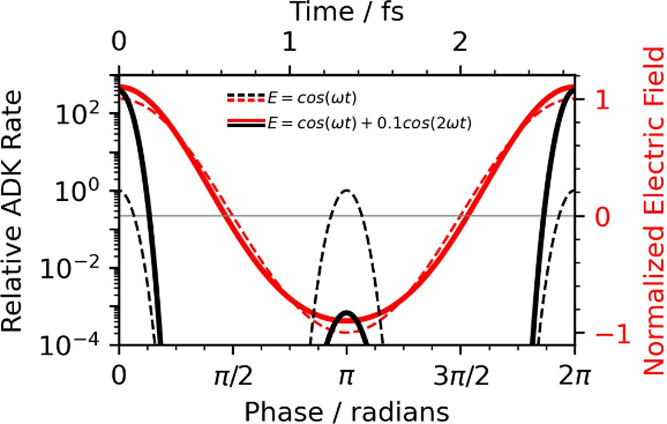
Relative ADK ionization rate (black) for a two-color laser field that comprises a small amplitude of the second harmonic with no phase offset (solid lines) compared to that for the monochromatic 800 nm field (dashed lines). The rates are plotted relative to the maximum rate in the monochromatic field, which is set to one (10^0^, black dashed trace) for easy identification of the drastic decrease in ionization rate at half-cycle (π radians) in the two-color case. The ionization rate (black) is plotted on top of the ionizing laser field corresponding to the monochromatic (dashed red) and two-color (solid red) cases for direct comparison of the field amplitudes.

### Phase Matching

In nonlinear optics, phase matching refers to a zero difference or mismatch between the wave vectors of the incident and generated waves (*Δk* = 0), such that output waves travel at the same phase velocity (ω/*k*) and combine constructively. In nonlinear crystals, this is most commonly achieved using birefringent materials that have different refractive indices along the crystallographic axes, such that perpendicular polarizations allow independent control of the phase velocities of the two waves (fundamental and second harmonic) due to the material’s anisotropy and frequency-dependent dispersion properties. In HHG, phase matching remains a central goal to ensure multiple, broadband harmonics are generated constructively by matching their phase velocity with that of the driving field. The propagation of a laser field in a gaseous medium is defined by its wave vector, which can be written as

$$k=\frac{2\pi }{\lambda }+\frac{2\pi N_{a}n\left(\lambda \right)}{\lambda }-N_{e}r_{e}\lambda$$

where *N*_*a*_ is the number density of neutral atoms, *n*(λ) is the linear refractive index per neutral atom density at wavelength λ, *N*_*e*_ is the density of free electrons and *r*_*e*_ is the classical electron radius.^123^ We note the opposite signs of neutrals and electrons in the above equation. The classical expression of phase-matching for the *q* ^th^ harmonic order is

$$\Delta k_{q}=k_{Xray}-qk_{laser}=0$$

$$\Delta k_{q}=\frac{2\pi q}{\lambda }\left(1-\eta \right)P\Delta n-P\eta N_{atm}r_{e}\lambda \left[\frac{q^{2}-1}{q}\right]$$

where a pressure term, *P*, is substituted for the neutral atom density, and *N*_*atm*_ corrects for the atom density at atmospheric pressure. Here, η represents the ionization fraction, *Δn* represents the difference in the refractive indices of the neutral gas at atmospheric pressure *Δn* = *n*_*laser*_^*atm*^ – *n*_*x*–*ray*_^*atm*^, and the X-ray wavelength is taken into account through the relation λ_*Xray*_ = λ/*q*.

When an electromagnetic beam passes through a focus, it experiences a phase shift π, known as the Gouy phase shift (Figure 14b).^124^ The shift is well described through the Rayleigh range as $\Delta \phi_{\mathrm{Gouy}}=-\arctan\left(\frac{z}{z_{R}}\right)$ , or in terms of the wave vector, $k_{\mathrm{Gouy}}=\frac{d}{dz}\arctan\left(z/z_{R}\right)\approx 1/z_{R}$ .^125,126^ Therefore, when harmonics are generated by focusing directly into the medium (*i.e.*, gas cell or gas jet), this phase offset must be corrected by adding the term *Δk*_*Gouy*_ = (*q* – 1)/*z*_*R*_ to the phase matching equation.^107,126^ Capillary waveguides provide greater interaction length and precise control over the phase velocity of the driving beam. The phase velocity is determined by the geometry of the waveguide and is well described by Bessel functions.^127^ A geometric term, which accounts for the normal dispersion of the waveguide, replaces the Gouy phase and is given by $-\frac{u_{\mathrm{nm}}^{2}\lambda }{4\pi a^{2}}$ , where *u*_*nm*_ is the *m*th root of the *J*_*n*–1_(*z*) Bessel function and *a* is the inner diameter of the waveguide.^123,128^

**Figure 14:**
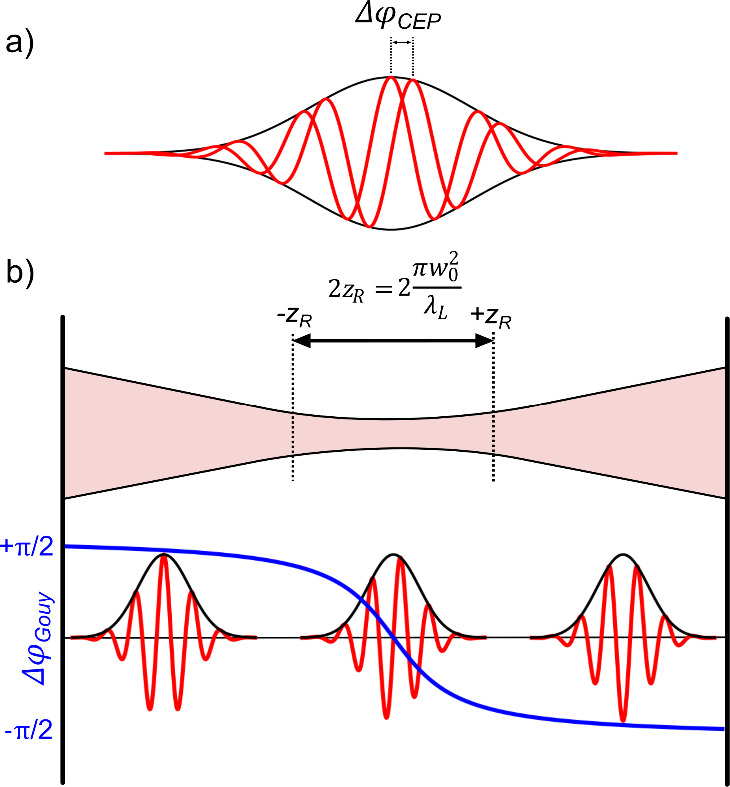
(a) Representation of carrier envelope phase offset, *Δφ*_*CEP*_, between a Gaussian pulse envelope and the underlying carrier frequency and (b) the evolution of the carrier envelope phase through a focus, defined by the Gouy phase shift across the Rayleigh, *z_R_*, set by the beam waist, *w*_0_, and the wavelength of the beam, λ_*L*_. Through the focus, a beam which starts as a cosine function with respect to the carrier envelope (*Δφ*_*CEP*_ = 0) will evolve as a sine function at the focus (*Δφ*_*CEP*_ = π/2), then a negative cosine on the opposite side of the focus (*Δφ*_*CEP*_ = π).

The three terms that make up the phase-matching equation describe contributions to the wave propagation from neutral atoms, free-electrons, and geometric dispersions (described by either the waveguide term or the Gouy phase). These phase-matching terms provide handles to tune the nonlinear conversion efficiency, most notably by balancing the neutral atom and free-electron density via the gas pressure and incident laser intensity.^123,128^ There exists some critical ionization fraction, η_*c*_ (which is typically <5%), for which the pressure dependent dispersions are balanced and the wave vector mismatch, *Δk*, is minimized.^94,123,128^ In sufficiently strong, ultrashort laser fields with high peak intensities capable of producing a laser-induced plasma, the pulse can become deformed along the propagation axis due to plasma-induced refractive index changes that cause defocusing and substantial reshaping of the pulse (recall Figure 9b). This time-dependent variation in the pulse shape causes microscopic deformations of electron trajectories along the propagation axis. Should these photon pulses with varying energies combine constructively, the initial high intensity enables the extension of the cutoff energy and overall bandwidth of the resulting HHG spectrum. In fact, high intensity, few-cycle pulses have demonstrated a notable extension of the cutoff energy.^129–131^ Phase matching models have been developed for this nonadiabatic regime to compensate for the dispersive plasmas caused by extremely high peak intensities of >4 × 10^15^ W/cm^2^.^115,132–134^

## 5 How are XUV Pulses Characterized?

A typical high harmonic generation spectrum presents as multiple peaks with a periodicity dictated by the symmetry of the ionizing field. The first harmonic spectra collected^135^ had a definitive shape (schematic in Figure 9c), with three prominent regions of interest. The first of these regions shows a progressive decline in intensities at low orders, which can be thought of as harmonics generated in the perturbative regime of intensities, *i.e.*, as χ^(*n*)^ processes. The next, at intermediate harmonic orders, is a broad region of harmonics with nearly identical intensities, resembling a plateau. Finally, a sharp cutoff at the highest produced harmonic orders is identified, the energy of which is dictated by the prototypical cutoff equation discussed above.

### Harmonic Spectra

Early theoretical work focused on developing a model that explained the process of high harmonic generation while accounting for its familiar shape.^136–139^ In general, low order harmonics can be described in the perturbative regime; thus, their intensities fall off as a power law, *I* ^*q*^, where *q* is the harmonic order and *I* is the incident laser intensity.^139,140^ A simple-man explanation as to the near equal intensities in the plateau region before a precipitous drop at the cutoff, is argued in the semiclassical picture. For example, when considering the electron trajectories capable of producing intermediate harmonic orders in the plateau region, there are two contributions (long and short, Figure 11); however, as the cutoff is approached, there is only one unique trajectory to consider.^107^ In other words, there is equal probability of producing the intermediate energies from short and long trajectories that fall off rapidly as progressively higher energy trajectories become less likely.

### Bandwidth

XUV pulses present a broad bandwidth, spanning from perturbative ordered harmonics up to the theoretical cutoff energy. The expected spectral bandwidth depends on the generating gas, the wavelength of the driving laser, and the pulse duration of the driving source. Sources driven by 40 fs pulses at 800 nm central wavelength typically produce a bandwidth of 20–40 eV, depending on the gas source. In this XUV range, commonly used noble gases include argon, neon, and helium, which generally produce harmonics in the range of 35–55 eV, 50–75 eV, and 80–120 eV, respectively. As has been demonstrated, the theoretical upper energy limit is inversely related to the driving frequency, so lower frequency IR (3.9 μm) driving sources are capable of producing much higher energies reaching over 1 keV.^96^

### Phase

As the ionization rate is dependent on the absolute intensity of the electric field, the relative phase of the field oscillations to the pulse envelope (or carrier-envelope phase, Figure 14a) becomes critical. In longer duration femtosecond pulses, where several optical cycles are contained (*e.g.*, ∼ 8 optical cycles within the full-width at half-maximum of a 35 fs 1/*e* ^2^ pulse), slight phase offsets of a few hundred attoseconds hold minimal effect on the maximum time-averaged intensities (*e.g.*, > 0.01% through a full π/4 phase shift). However, carrier envelope phase stability becomes critical for few optical cycle pulses (say 6 fs, containing only 2 cycles within the full-width at half-maximum) for which small phase offsets can change the time-averaged electric field intensity by up to 0.5%, which affects the ionization phase, alters the electron kinetic energy and therefore affects the energy spectrum produced.

The carrier-envelope phase (CEP), more precisely defined by the phase offset between the peak of the pulse envelope and the nearest peak of the electric field (Figure 14a), must be precisely controlled for attosecond experiments.^141,142^ For a long time now, commercial oscillators can be purchased with CEP stabilization specifications. This is often done in a complex scheme of multiple feedback loops, where an acousto-optic modulator (AOM) controls the oscillator pump beam to induce a refractive index change in the Ti:sapphire crystal, producing an intentional CEP jitter. The CEP of pulses are measured using an interferometer which detects the beat frequency of a pulse overlapped with its second harmonic. The AOM is adjusted until the CEP jitter matches some harmonic of the laser repetition rate that can be programmed into the pulse picker, such that each pulse selected for amplification has the same CEP.^143^ For Gaussian pulses, this phase stability becomes further complicated by the introduction of the Gouy phase (Figure 14b), which, as mentioned previously, causes a time varying shift of the carrier envelope phase through the focus.^125^

### Pulse Duration

The generated XUV pulse duration is dictated by the pulse duration of the incident near-infrared (NIR) source. For multicycle laser pulses (*e.g.*, 35 fs) that contain multiple optical cycles producing ionization events and therefore attosecond photon pulses, the overall XUV pulse duration is a summation of the attosecond pulse train. However, for shorter, few cycle pulses (*e.g.*, 6 fs), the intensity variation becomes comparable to a single optical cycle; thus, only one cycle is capable of producing an ionization event, resulting in an isolated XUV pulse. It has been demonstrated that the maximum pulse duration capable of producing an isolated attosecond pulse is 6 fs.^141^

For attosecond pulses, where the pulse duration is significantly shorter than an optical cycle of the driving NIR field, streaking experiments can be performed to better understand the temporal profile of the XUV pulse.^143–147^ In krypton, for example, the oscillation of an NIR electric field transfers momentum to electrons.^141^ When these electrons are ionized to form photoelectrons, that momentum transfer leads to a ponderomotive shift of their detected kinetic energies. The result - for an XUV pulse overlapped with an NIR pulse in krypton atoms, the detected kinetic energy will depend on the relative phase of the NIR pulse with which the XUV is overlapped, allowing a direct mapping of the phase of the NIR pulse. The profile is dependent is dependent on the pulse duration of the attosecond pulse, therefore, the attosecond pulse duration can be extracted by fitting procedures to the streaked data.

### Photon Counts

High harmonic generation is regarded as a low-efficiency nonlinear optical technique, with efficiencies generally around 10^–7^ – 10^–5^.^94,123,148–152^ For a 1 mJ driving 800 nm pulse, this low conversion efficiency translates to a total XUV photon flux on the order of 10^8^ – 10^10^ photons/pulse. The efficiency of the process is dictated by all the factors in the generation process itself, including laser intensity, pulse duration, gas pressure, interaction length, driving frequency, and absorption cross-section of the medium. The total intensity of the harmonic field produced is proportional to the macroscopic phase-matching factor, *Δk*, according to the relationship $\left|E_{q}{|}^{2}\propto \right|\frac{\sin\left(\Delta \mathrm{kL}/2\right)}{\Delta k}{|}^{2}$ .^104,148^ Here, *L* is the interaction length and *E*_*q*_ is the amplitude of harmonic order *q*. The complexity of the technique makes it difficult to predict efficiency, but comprehensive reviews on phase matching can provide some guidance.^148^ The total flux per pulse can be determined by integrating the total counts on a detector and correcting for the quantum efficiency of the detector, the efficiency of the grating used, and the expected loss from optics and filters. The Center for X-ray Optics X-ray database is an invaluable resource for accessing reflectivity and transmission curves for various materials to estimate photon loss, noting that diffraction gratings also vary in efficiency at different diffraction orders. For reflections off a gold surface at 5°, one can expect 10–20% loss in the XUV, while transmission through 200 nm of aluminum can cost 30–40%. In the soft X-ray (160–300 eV), an average number of 10^5^ soft X-ray photons per pulse reach the interaction region, and less than 10% of these photons get detected when the grating diffraction efficiency in the first order, filter transmission, and detector quantum efficiency are taken into account.^153^

### Shot Noise and Fluctuations

Noise in an XUV experiment originates an XUV experiment originate predominantly from the fluctuations of the high harmonic spectra. Shot noise, which arises from the discrete nature of photons resulting in an inherent randomness in the detected flux, often defines the theoretical minimum of the noise floor.^154^ Shot noise scales with the square root of the total number of photons detected σ_*shot*_ = $\sqrt{N}$ . Thus, the signal-to-noise ratio scales as $\mathrm{SNR}=\frac{N}{\sqrt{N}}=\sqrt{N}$ , which naturally decreases in regions of low photon flux (Figure 15). Pulse-to-pulse variations of the high harmonic source, like any nonlinear process, are tied directly to the pulse-to-pulse fluctuations of the laser source. Considering a χ^(2)^ process, such as second harmonic generation – the intensity of the second harmonic produced is proportional to the square of the incident intensity, *I*(2ω) ∝ *I* ^2^(ω).^119^ In other words, the fluctuations of a χ^(2)^ process will vary as the square of the fluctuations of the incident intensity. In comparison with white-light generation, incident intensity fluctuations will affect both the intensity *and* the frequency spectrum of the produced supercontinuum. The spectral profile of the produced white-light is not as straightforward a relation to the incident intensity, though numerical simulations have proven successful in solving for nonlinear corrections to the Schrödinger equation.^155,156^

**Figure 15:**
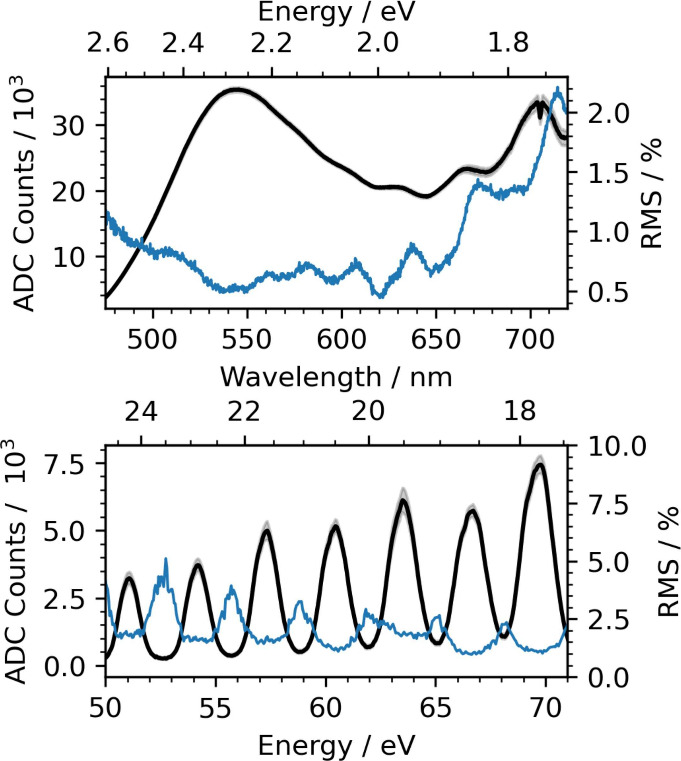
Comparison of the stability of a white light spectrum generated in a sapphire window (top) versus a harmonic spectrum generated in neon (bottom) measured in the authors’ laboratory. Analog-to-digital conversion (ADC) counts are obtained using exposure times of 10 μs (top) and 100 ms (bottom). The spectra represent an average of 1000 measurements (black trace) with error bars (gray) representing the standard deviation of these measurements. The blue trace denotes the root-mean-squared (RMS) fluctuations as a percent.

Despite the complexity of the problem, experimentally, the stability of white light is generally considered only slightly worse than (or even comparable to) the energy stability of the laser source itself (in general, a 1% root-mean-square or RMS fluctuation can be expected compared to 0.2% RMS fluctuation of the laser source).^103,156,157^ The RMS stability of a white light spectrum in sapphire collected over 1000 laser pulses in our laboratory is shown in Figure 15. Incident intensity fluctuations will also affect the intensity and frequency spectrum of the generated harmonics, similar to white light. Not only will the relative intensities of the harmonics produced change, but it is well understood that subtle variations in the generating conditions (gas pressure, intensity, relative focal position) will vary the frequency of individual harmonic peaks.^158–160^ This causes a derivative-like profile of the RMS stability of a spectrum, and frequency shifts are strongly correlated with pulse-to-pulse intensity fluctuations.^161^ Due to the complexity of the problem, small intensity fluctuations in the driving laser (0.5% RMS) can translate to 2–20% spectral power density fluctuations of the XUV, and is dependent on the photon energy being measured.^161,162^

High frequency fluctuations from shot noise are difficult to correct for, but increased averaging time often limits their contribution to the final spectrum. Further, the high level of correlation between the laser fluctuations and the generated harmonic spectrum allows for correction by noise correlation or edge-pixel referencing.^161,163,164^ Low frequency fluctuations that originate from pointing instability, air-currents, table vibrations from vacuum pumps, gas pressure variation, *etc.* are more difficult to correct for with increased averaging, as their slow variation requires longer observation times. When taking a measurement (say a static absorption measurement that requires a separate sample and reference spectrum to be taken), if the sampling time is sufficiently long such that these drifts in the spectrum occur between measurements, it will modulate the resultant absorption spectrum from improper referencing. A common practice is to mark the upper limit of this sampling time by identifying the dominant low- and high frequency spectral fluctuations. This can be done by measuring a large number of XUV spectra at short time intervals, then performing a Fourier transform to extract the frequencies that contribute to the fluctuations.^165^ One can then easily delineate between fast shot noise and slower drifts.

## 6 Typical Tabletop HHG Apparatus—Nuts and Bolts

The attenuation length of photons ranging 20–100 eV in atmospheric pressures of nitrogen is just a few microns, thus XUV beams can only propagate in vacuum, requiring high-vacuum systems. Fortunately, the refractive index for XUV photons is just below 1 for most materials, enabling a critical angle for total external reflection at very narrow incidence angles. This imposes two requirements for the design of a tabletop X-ray beamline: (1) high vacuum and (2) grazing incidence optics. Figure 16 shows a schematic of an example HHG apparatus. From left to right, the NIR beam (red) overlapped with a small portion of its second harmonic (blue) is focused into a semi-infinite gas cell (SIGC). The generated harmonics diverge from this point with the remaining visible-IR light, before passing through a thin metal foil mounted on a push–pull adapter between the first (generation) and second (focusing) chambers (and in between each chamber thereafter, namely, sample and spectrometer chambers). This foil serves to filter out remaining photons from the fundamental NIR beam before reaching a refocusing optic in the second chamber that houses a toroidal focusing mirror. The harmonics are focused into a sample mounted on a motorized XY-stage in the third chamber. Finally, the beam reaches a diffraction grating where it is spatially dispersed by photon energy and detected on a charge-coupled device (CCD). Each component is discussed in detail below.

**Figure 16:**
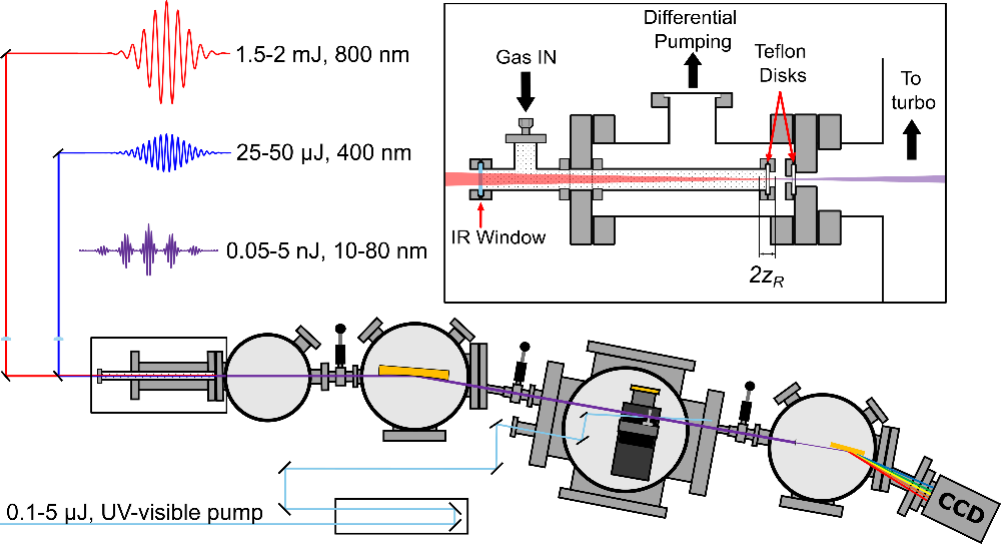
Schematic drawing of an HHG apparatus (implemented in the authors’ laboratory). From left to right, the vacuum chambers constitute the source of high-harmonic generation, followed by XUV refocusing, sample interaction, and dispersive detection on a charge coupled device (CCD). The entire beamline fits on a standard 12 ft × 4 ft optical table with 1.5 ft and 2 ft to spare at both ends of the table. The inset in the upper right corner is a detailed drawing of the differentially pumped semi-infinite gas cell. The cell begins with a standard CF 1.33 in. tee, that is capped on one end with an IR window and has a 1/4 in. Swagelok adapter for the gas feed. This gas cell is connected to a CF 1.33 in. to CF 4.5 in. zero-length reducing flange that couples with a 6 in. long, standard CF 1.33 in. full-nipple capped with a PTFE (Teflon) disk. The long volume, which constitutes a semi-infinite gas cell, is held inside a CF 4.5 in. to CF 2.5 in. reducing tee, with the CF 2.5 in. port connected to a roughing pump for differential pumping.

### Source

As discussed, high harmonics are generated by focusing high intensity (∼10^13^–10^15^ W/cm^2^) and few-cycle NIR pulses into, typically, a noble gas. In the early days, this was done with a small jet of gas.^135^ Gas jets, which consume large amounts of gas, are still utilized today; however, the gas density has a complex three-dimensional profile that strongly affects harmonic generation, requiring precise alignment.^166^

With the development of the field, more efficient generation methods have been introduced. The most common method is a semi-infinite gas cell (SIGC),^160,167,168^ which comprises a long cell with an IR window at one end and a pinhole at the opposite. As the beam is meant to focus at the pinhole for optimal transmission of the generated harmonics, gas cells are usually capped with a thin metal foil or polytetrafluoroethylene (PTFE) disk, capable of withstanding the pressure differential, but thin enough to be drilled through by the high intensity beam. This ensures that the pinhole is always aligned with the incident beam and is the minimum allowable diameter so that the beam fully passes through while wasting minimal gas. The generation of high harmonics depends on the quality of the incident beam mode. This drilling technique provides a simple rule of thumb: if the incident beam drills through the foils, the beam mode is of sufficient quality to generate high harmonics.

The inset of Figure 16 shows a schematic drawing of a typical SIGC. In this case, the SIGC volume is separated from the turbo-molecular pump by two thin PTFE disks. This creates a volume between the two chambers that can be differentially pumped by a separate roughing pump to limit the load on the turbo. Since the focal length of the beam is long (75 cm), the Rayleigh range of the focused beam is long (>6 mm), so the beam is still capable of drilling these separated disks. However, a thinner PTFE is preferred after the differentially pumped volume, as optimal phase matching requires that the focal volume be mainly inside the gas cell, causing a divergent beam after the initial pinhole.^160^

In this schematic, there are four regions of different pressure: (1) the high-vacuum chamber connected to the turbo pump (∼10^–7^–10^–6^ Torr), (2) the differentially pumped volume connected to a roughing pump (∼10^–2^ Torr), (3) the SIGC (∼10^1^–10^2^ Torr), and (4) ambient atmosphere outside the IR window (∼10^3^ Torr). These pressure differentials across thin barriers create an important consideration when choosing the thickness of the separating media for the SIGC (*e.g.*, PTFE disks and glass windows): too thin, and the pressure differential can cause the disk or IR window to rupture; too thick, and the beam may not drill through. The integrity of the disk is determined by a ratio of thickness to free-standing diameter along with the flexural strength of the material (∼ 50 MPa for PTFE). A desired ratio for thickness to free-standing diameter, $\frac{t}{D}$ can be calculated with the equation $\frac{t}{D}=\sqrt{\frac{{\mathrm{KpS}}_{f}}{4Y}}$ , which relates the ratio to the pressure differential, *p*, the flexural strength, *Y*, the desired safety factor, *S*_*f*_ (where *S*_*f*_ > 4 is considered adequate), and a proportionality constant, *K*, which varies between 0.75 and 1.125 for a clamped or free edge, respectively.^169^ We have found that with PTFE, a 1/16 in. thick disk does not distort for a free-standing diameter of 10 mm, while a 0.5 mm thick disk is allowable for a 7 mm diameter.

Gas cells, though the simplest to implement, have limited focal interaction lengths with the gas, dictated by the focusing parameters of the driving laser. This has been circumvented with the introduction of hollow capillary waveguides as a generating source.^123,128^ Waveguides give an increased interaction length and careful control over the phase velocity of the incident beam.^170^

### Filtering

After harmonic generation, the residual visible/IR photons need to be removed by selectively filtering from the XUV. This is done with thin metal foils that allow significant transmission of the high energy photons while remaining opaque to visible and IR. The identity and thickness of the metal depends on the desired energy range. Aluminum is a suitable choice for energies between 20 and 72 eV with an attenuation length on the order of 100s of nm. However, for energies above 72 eV (above the L_2,3_ edge of Al), zirconium is typically used, although the attenuation length is about half that of aluminum, requiring thinner and more delicate foils.

Filters (200 nm Al, Lebow) are mounted on spring-loaded, push–pull linear motion feedthroughs in between chambers (Figure 16) allowing filters to be easily inserted or removed to vary the amount of XUV attenuation or to visualize the IR/visible beam for alignment purposes. Each filter removes 30–40% of the incident XUV photon flux. The X-ray database maintained at the Center for X-ray Optics (Lawrence Berkeley National Laboratory) provides useful predictions of the transmission of the XUV flux through various materials and provides an accessible source that should be consulted when considering appropriate choice of filters.

### Focusing

After generation, the diverging XUV beam must be refocused into the sample of interest. As discussed, XUV reflection occurs most efficiently at grazing incidence (very wide angle of incidence). In this geometry, a toroidal shaped optic provides two separate circular radii of curvature to separately focus the sagittal (vertical) and tangential (horizontal) dimensions of the beam, accounting for the angle of incidence. In general, the radii of curvature must be chosen such that the focal point (*i.e.*, the pinhole of the SIGC) is reimaged on the sample. For circularly/spherically curved mirrors, the tangential (*f*_*tan*_) and sagittal (*f*_*sag*_) focal lengths vary with the angle of incidence, θ, as *f*_*tan*_ = *R*_*tan*_ (cos θ)/2 and *f*_*sag*_ = *R*_*sag*_/(2 cos θ), where *R*_*tan*_ and *R*_*sag*_ represent independent radii of curvature for the tangential and sagittal planes.

### Light-Matter Interaction

Samples are mounted on motorized stages for easy manipulation in vacuo. Despite the high vacuum requirements, methods have been developed for measurement of samples in the solid, gas, and even solution/liquid phase. The preparation and selection of these samples is a delicate process as the attenuation length of most materials is tens to hundreds of nanometers. Solid state samples are typically prepared as some thin film deposited on a semitransparent, thin membrane such as silicon nitride, although organic polymer substrates have been shown to be a cheaper and more transparent alternative.^171^

In the gas phase, sample cells are designed with a set path length and drilled pinholes at the entrance and exit. These pinholes (usually 150 μm-200 μm) allow the beam to enter and exit, while the use of optically transparent windows would attenuate the beam. The gas that exits the pinholes escapes into a large volume environment at very low pressures (<1 × 10^–3^ Torr), causing rapid expansion. Thus, the gas density outside of the nominal path length of the cell is negligible in a typical absorption measurement.^150^

Measurements in liquid or solution phase pose several challenges, the most significant being the very short attenuation length for liquids. Still, flow cells comprised of sandwiched silicon nitride windows separated by a polymer spacer, originally developed for XAS at synchrotron sources,^172^ have been successfully utilized in tabletop HHG experiments.^173^

A recent development has allowed surface sensitivity of solid-state samples via XUV reflection–absorption spectroscopy at the M_2,3_ edge.^174–179^ While the transmission mode probes the imaginary part of the refractive index, *k*, the reflectivity is sensitive to both the real and imaginary components, *N* = *n* + *ik*, complicating the measurement. XUV reflection–absorption spectra can be simulated in strong agreement with experimental results by calculating the real part of the refractive index, *n*, through a Kramers–Kronig transformation from the imaginary component, *k*, which reports on the attenuation of an incident light spectrum. The power of this relationship is that it allows one to deconvolve the real and imaginary components of the refractive index from one measurement, which independently report on the surface morphology and chemical state of the sample, respectively.^174^ Time-resolved XUV reflection–absorption spectroscopy has been applied in the study of surface electron dynamics and small polaron formation in single crystalline, polycrystalline, and functionalized hematite (α-Fe_2_O_3_),^177,179^ identification of defects at a NiO-hematite heterojunction,^178^ and hole-localization dynamics in various metal oxides and sulfides.^180^

### Detection

Detection at X-ray beamlines is done with a variety of different techniques. For example, X-ray fluorescence is often detected with an avalanche photodiode, where energy dispersion is performed by exploiting the Bragg-diffraction of some analyzer crystal. Detectors play an important role in the time-resolution of experiments at beamlines that generally produce long (10–100 ps) pulses. In these experiments, a fast time-resolving detector is able to “streak” the long pulse in time to provide ultrashort time information. This measurement is commonly done with the use of fast-response avalanche photodiodes or streaking cameras, which can spatially encode both the time and energy profile of a pulse.^36,181,182^

In tabletop HHG setups, where the temporal resolution is dictated solely by the pulse duration, energy dispersive detection on a spatially resolving detector is more common. Grazing-incidence, gold-coated gratings disperse the pulse by energy before detection, generally on an X-ray CCD or an imaging microchannel plate (MCP) assembly. Although an MCP’s phosphor screen still requires an imaging camera, their low quantum efficiencies in the visible and IR wavelength range forgoes the need of filtering the fundamental unlike using a bare CCD.^143,183^ Typical “front-illuminated” CCDs place the photoactive semiconductor behind the gating electrical components and insulation (typically comprised of polysilicon and SiO). For high energy photons, the limiting attenuation length requires a back-illuminated CCD such that the photons hit the bulk semiconductor directly.^184^ Although the bulk silicon substrate on which the CCD is manufactured must be etched away, this back-illumination enables quantum efficiencies between 20% and 80% (affected by the presence of absorbing elemental edges, *e.g*. silicon at 100 eV.).^185,186^

### Spectral Resolution

After the XUV pulse is spectrally dispersed, there are a number of variables that will affect the spectral resolution: the line spacing on the grating, the angular divergence of the beam, the distance from the grating to the detector, and the pixel size on the detector. Grazing-incidence, gold-coated diffraction gratings are required for high efficiency dispersion. X-rays incident on a reflective diffraction grating follow the grating equation *mλ* = *d*(sin α – sin β), where *m* is the order of diffraction, λ is the wavelength, *d* is the line spacing, and α and β describe the angles of incidence angle and diffraction, respectively, relative to the surface normal. As the beam is diverging at this point, it is necessary to refocus or otherwise account for the angular divergence prior to energy dispersion. This compensation is often achieved using a second toroidal mirror just before the grating with a focal length optimized to focus at the detector.^150^ Alternatively, it is common to use a concave grating that focuses and diffracts the beam.^187^

A uniform line-spaced grating, though unable to achieve a flat-field focus, can still achieve a considerable resolution of 200 meV.^150^ The large footprint (several cm) on the grazing incidence grating requires slightly different diffraction angles over the length of the optic for the energies to end at the same point on the CCD. Mechanically ruled, variable-line spaced gratings were developed for just this purpose.^188^ Concave gratings are designed with groove densities that vary across the length of the optic to account for the difference in diffraction angle needed to achieve flat-field focus. These gratings are designed with specific incidence angles and image-object distances in mind, so spectrometers are built around the specifications of the grating. With optimized concave, variable line-spaced gratings, a resolution as high as 10 meV is achievable.^189^

### Divergence

The divergence of the XUV beam derives from the divergence at the source. Similar to white-light continua, the divergence is not always the same as the divergence of the source. The divergence of a white-light continuum is dependent on the half angle divergence of the fundamental beam, the depth of the focal plane in the generating media, and differs for each produced frequency due to Kerr-lens effects.^102,190,191^ In general, the divergence of the continuum is larger than, albeit comparable to, that of the incident fundamental.^102,103^ In high harmonic generation, the divergence differs from what is expected for the incident beam (*i.e.*, in vacuum or air) as the beam propagation can vary dramatically for a high intensity beam in a plasma of ions and free electrons, and can even show multiple focal planes.^192^ It depends on several factors, such as the half angle divergence of the fundamental beam, the position of the focus in the generating medium, and even the identity of the generating medium.^128,150,167^ The divergence is further complicated with the use of waveguides.^193^

In a spectrometer for which the angular divergence in the sagittal (vertical) plane is preserved (*i.e.*, not focused), the angular divergence of the XUV beam can be measured from the vertical profile of the XUV spectrum and back-calculated by considering the distance to the harmonic source or image plane of the sample.^167,192,193^ Alternatively, the wavelength-integrated beam profile can be imaged directly by placing an X-ray CCD a known distance from the beam focus without energy dispersion by a diffraction grating.^150^ Despite its complexity, half angle divergence of an XUV source of 1–6 mrad is typical.^150,194–197^

### Spatial Resolution

The propagation limitations of XUV/X-ray photons (requiring the use of diffractive/grazing incidence optics due to abysmal attenuation lengths and high scattering factors) place constraints on the spatial resolution achievable with typical tabletop HHG sources. In other words, XUV photons cannot achieve spatial resolution near the fundamental wavelength limit by the traditional visible microscopy technique of using a high numerical-aperture lens.^198^ Indeed, in typical tabletop HHG sources, the spatial resolution is dictated by the long focal lengths used to enable the use of grazing incidence focusing optics, providing focal spot sizes on the order of tens of microns (typically 40–60 μm).^150,199,200^

An emerging technique has found a way to break this limitation by means of coherent diffractive imaging.^198,201–203^ This technique works on the principle of reconstructing an object from a wave diffracted off it by a coherent source. The image can be reconstructed by considering that the diffracted wave is proportional to the Fourier transform of the object.^204,205^ Thus, rather than using multiple optics to reconstruct an image of the object, the diffracted light is detected directly and phase information is recovered computationally with phase-retrieval algorithms that enable image reconstruction.^206,207^ This technique continues to evolve with the development of higher numerical-aperture beams and ptychography, which uses redundant information from overlapping areas of the sample to enable simultaneous reconstruction of the phase and amplitude.^208,209^ In all, this has enabled imaging with resolution as small as 0.9λ using a 13.5 nm high-harmonic light source.^203^

### Temporal Resolution

The temporal resolution of an XUV experiment is limited by the pulse duration of the driving laser. Typical femtosecond Ti:sapphire lasers have pulse durations that minimize at 25 fs but are most commonly used in the 35–40 fs range. In a femtosecond pump-XUV probe experiment, an excitation pulse (UV/vis/NIR) is routed through a mechanical delay line and then generally meets the probe pulse at a shallow crossing angle (<5°). When these beams are focused to relatively small spot sizes (∼100 μm), these shallow crossing angles impose a timing mismatch between the edges of the beam of tens of fs (30 fs for a crossing angle of 5° and a spot size of 100 μm). When the cross correlation of the pump and probe beam is on the order of 50–100 fs, this loss of resolution is not significant. However, for single attosecond pulses, this small crossing angle can completely negate the time-resolution achievable. To circumvent this, pump–probe experiments with attosecond pulses are performed in a collinear orientation, where the pump pulse is routed using an annular mirror with a hole drilled through the center to allow transmission of the XUV probe.

In generating attosecond pulses, Ti:sapphire laser pulses must be spectrally broadened to overcome the transform limit and undergo temporal compression. This is done with hollow core fibers, which broaden the pulse via self-phase modulation giving a larger frequency bandwidth for a shorter accessible transform limit.^143,200,210–212^ Then, pulses can be compressed via typical means, such as chirped mirror pairs and fused silica wedges, achieving pulses as short as a few femtoseconds. As discussed previously, a pulse of 6 fs is sufficiently short to produce a single isolated attosecond pulse.^213^ Furthermore, techniques such as double optical gating and ionization gating allow longer multicycle driving pulses directly from a laser amplifier to be used for generating isolated attosecond pulses.^214–217^

## 7 XUV Spectroscopy: Overview of Data Analysis

Extreme ultraviolet or soft X-ray spectroscopy is a form of electronic absorption spectroscopy that measures the absorbance (in steady-state or static conditions) and differential absorbance (under optical pump-induced conditions) of core electrons. Therefore, formal data analysis methods bear close resemblance to the techniques of broadband electronic transient absorption spectroscopy. Here, we provide a brief overview of data analysis, drawing on six important considerations that Beckwith et al. provide in their comprehensive review of methods in transient electronic absorption spectroscopy.^218^

### Background Correction, Steady State

Because of the alternating peaks and valleys in HHG, the absorbance of molecular and solid samples can contain sharp discontinuities when the flux is low in the valleys. Attar et al. note the appearance of discontinuities in the XUV absorption spectrum of CH_2_IBr.^73^ Lin et al. suggest a mitigation scheme that uses a broad rather than a sharp (*i.e.*, structured) dispersed HHG spectrum.^199^ Femtosecond soft X-ray pulses near the carbon K-edge suffer from spectral modulations to a lesser extent due to the closer spacing of HHG peaks from a smaller driving laser frequency (longer wavelength infrared) that make the valleys far less pronounced. Attosecond X-ray pulses nominally produce broader spectra with lower harmonic modulations, but high harmonic modulations are never altogether absent. A good practice is to report spectra resulting from the average of multiple measurements over different days. This approach ensures that the valleys in the harmonic spectra are somewhat different day-to-day, generating confidence in assignments made especially past an absorption edge where photon counts are naturally diminished.^53^

### Background Correction, Transient

Time-resolved XUV absorption data have no contamination due to pump scatter because of the vast separation in the photon energies of pump and probe, *i.e.*, as it is a nondegenerate pump–probe spectroscopy. Optical pump pulses are usually in the UV and visible to near-IR range, varying between 200 to 800 nm. Tabletop XUV photons between 30 and 120 eV correspond to 41 to 10 nm, and near the carbon K-edge (284 eV) are closer to 4 nm in wavelength. As shown schematically in Figure 17a,b, the first-order diffraction for a HeNe laser (633 nm) and the fundamental (800 nm) output of a Ti:sapphire laser deflect closer to 100°–60°, while the XUV beams used in the grazing incidence geometry deflect closer to 2°–3°. The CCD chip is nominally one inch wide and will not register optical pump pulses in first-order diffraction.

**Figure 17:**
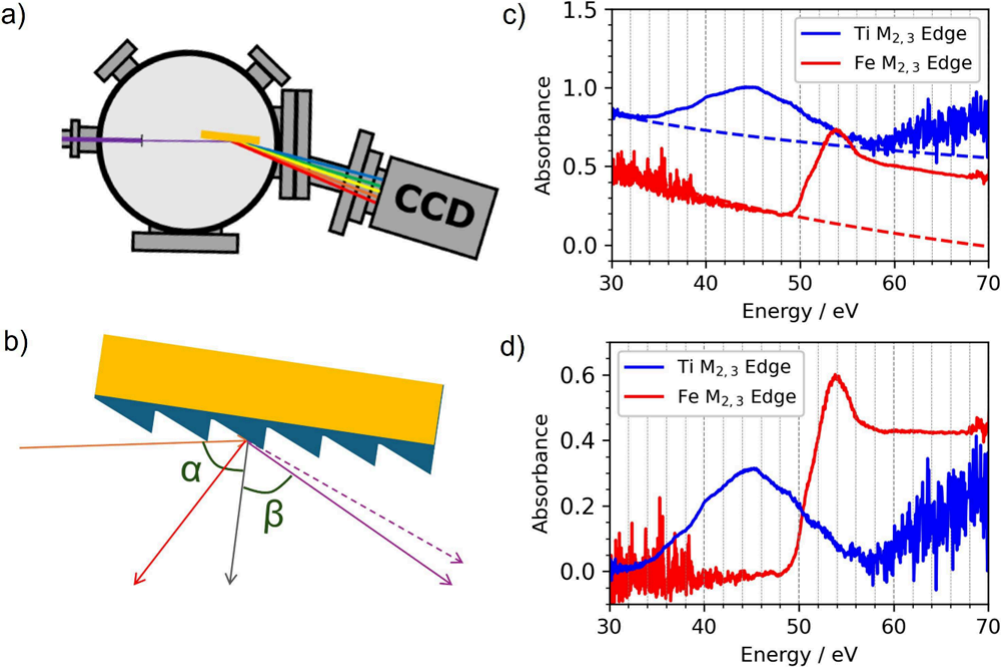
(a) Design of an off-center, flat, plane-ruled XUV grating spectrometer implemented in the authors’ laboratory. (b) Schematic of an XUV grating showing the angles of incidence (α) and diffraction (β) with respect to the surface normal (gray arrow). Dashed purple denotes the zero-order diffraction (specular reflection), solid purple arrow is the first-order XUV diffraction, and red arrow is the first-order HeNe (633 nm) diffraction. (c,d) Raw (c) and baseline-subtracted (d) XUV absorption spectra of Ti (10 nm) and Fe (10 nm) thin films deposited on 100 nm thick Si_3_N_4_ membranes, measured in the authors’ laboratory.

A caveat is that the zero-order diffraction must be meticulously blocked as the zero-order is the same specular reflection for XUV, the driving wavelength (near IR, 800 nm), and the pump pulse (UV–vis), which makes them all copropagating for the same angle of incidence. This selective blocking is usually achieved by installing thin metal filters with strong optical attenuation while maintaining a higher relative transmission for XUV. On the flip-side, valuable XUV photons are sacrificed in this process; for example, 100 nm thick aluminum foils usually cut down the XUV flux by slightly less than one-half, as discussed earlier. It would be clever to utilize zero-order diffraction, nominally blocked or rejected in XUV transient absorption setups, for active noise correction via reference and balanced detection and Heinrich et al. report implementing this scheme in their XUV setup using two diffraction gratings.^219^

A unique form of background contribution XUV spectroscopists have to consider is background due to nearby atomic edges in the sample. This point is best exemplified in the work of Schnorr et al. and Yang et al.^70,153^ Schnorr et al. report joint sulfur L_1_-edge, sulfur L_2,3_-edge, and carbon K-edge measurements of dimethyl disulfide and Yang et al. reported joint chlorine L_2,3_-edge and carbon K-edge measurements of chloroiodomethane (Figure 18a,b). Every higher atomic edge absorption rises above the trailing background from the previous edge. This feature is also noted in the XUV spectra of alkyl halides, where photoionization of valence electrons causes a substantial constant background at the shallow edges (M_4,5_ and N_4,5_) of bromine or iodine atoms.^24,25,73^ Note that power law decays are subtracted as background for solid state samples deposited on a substrate (Figure 17c,d), and in some cases of molecular samples a cubic background correction is applied.^220,221^

**Figure 18:**
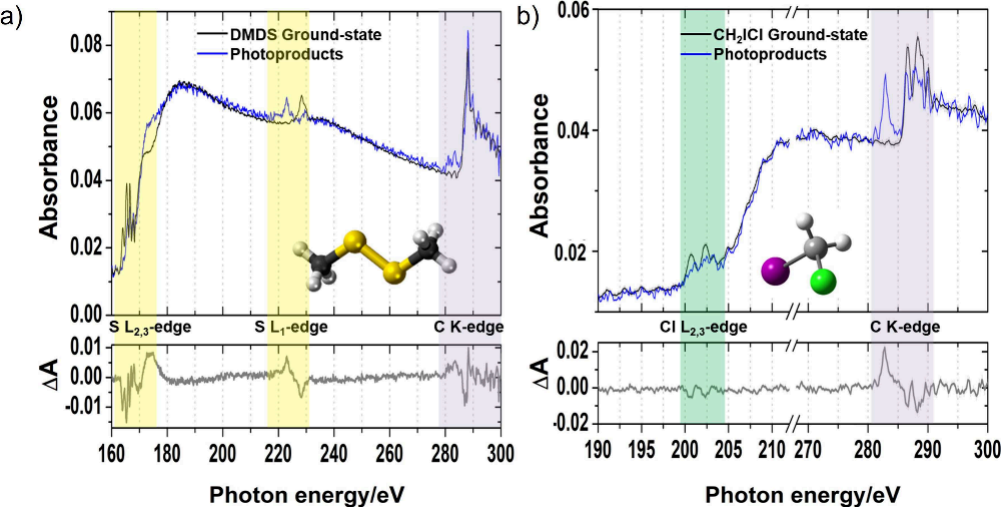
Steady-state and transient absorption spectra of (a) dimethyl disulfide (DMDS) and (b) CH_2_ICl revealing key differences between the molecular ground state and the radical photoproducts. Reprinted with permission from ref 28, Copyright 2018 American Chemical Society, Adapted with permission from ref 153, Copyright 2019 American Chemical Society, and ref 70, Copyright 2018 American Chemical Society.

Finally, the broadband nature of the XUV probe often leaves the ability to subtract signals from edge pixels without any sample absorption.^163^ This method demonstrably improves the signal-to-noise ratio. Interested readers are referred to two-dimensional infrared spectroscopy work, where edge-pixel referencing has been developed and applied to suppress correlated baseline noise.^222,223^ Of interest, a principal component regression method is also reported for the removal of correlated background noise in an HHG source while circumventing the need to identify pixels free of any signal.^164^

### Energy and Time Calibration

The periodicity in the harmonic spectrum and knowledge of the properties of the generating medium are good rough indicators of energy calibration as a starting point. For example, one can literally count the number of odd harmonics by tuning the intensity and gas pressure to get a sense of the spectral coverage even without measuring an absorption spectrum. Metal filters used in the beamline to block residual infrared can also provide additional reference points for quick calibration purposes. The grating equation is used in home-built X-ray spectrometers for a more precise energy calibration in electron volts from pixels on the CCD chip. High precision calibration can be obtained day to day by measuring core-to-Rydberg transitions in atomic gases or known molecular core-to-valence absorption resonances. Care must be taken to have reference points on both ends of the CCD chip (*i.e.*, toward lower and higher pixels) to avoid any errors in the calibration process due to extrapolation. A Jacobian transformation converts raw harmonic XUV spectra measured in the pixel space to real spectra in the energy space.^224^

The instrument response function (IRF) is a cross-correlation of the pump and probe pulses in a gas medium. The IRF is usually measured as a ponderomotive shift of the core-to-valence resonances of rare gas atoms such as xenon and argon. The signals expected for the ponderomotive shifts and a measured Gaussian IRF are presented in Figure 19. The center of the Gaussian width of the instrument response function can be used as a measure of time-zero. In other experiments, charge carrier absorption in hematite has been used to determine IRF in femtosecond pump–probe experiments.^62^ Rare gas atoms are the preferred approach for all attosecond techniques.

**Figure 19:**
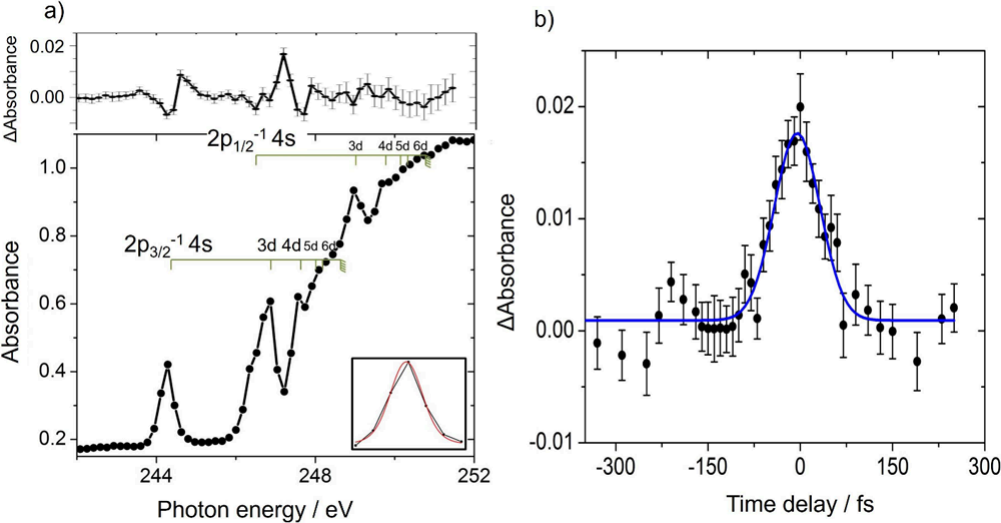
(a) (Top) Ponderomotive shift of the atomic core-excited Rydberg state resonances in argon in the presence of a 266 nm optical field. (Bottom) Argon core-to-Rydberg state resonances at the L_2,3_ edge that are used to calibrate the spectrometer and determine the resolution from a fit of the full-width-at-half-maximum of the main resonance as shown in the inset. (b) The full-width-at-half-maximum of the soft X-ray cross-correlation measured as the change in absorbance of the 2p_3/2_^–1^3d core-excited Rydberg state of argon in the presence of the 266 nm field and as a function of the pump (soft X-ray)-probe (optical, 266 nm) time delay transcribes an instrument response of 90 fs. The peak center in this Gaussian cross-correlation denotes time-zero in the experiment. Adapted with permission from ref 53. Copyright 2017 American Chemical Society.

### Coherent Artifacts and Chirp Correction

Coherent artifacts are absent in optical pump-XUV probe spectroscopy for the same reasons that data have no contamination due to pump scatter, *i.e.*, this is a nondegenerate pump–probe method. The absence of coherent artifacts presents a key advantage in measuring dynamics in the Franck–Condon region immediately upon excitation, as discussed earlier. A chirp correction or (probe wavelength dependent time-zero) is not needed within the detectable bandwidths of tabletop HHG sources because reflective optics are used in vacuum. The spread of recollision times of short and long electron trajectories that generate different photon energies leads to an “attochirp”, that can be safely ignored in the case of femtosecond-time-resolved experiments.

### Spectral and Kinetic Analysis

XUV spectroscopy can measure line shape functions such as Lorentzian, Fano, Voigt, and Heaviside with a high accuracy, as well as shape resonances in molecular spectra. A Lorentzian to Fano line shape interconversion is exemplified in the work of Ott et al. for atomic He absorption.^225^ Fano resonances are found in other atomic systems when there is sufficient coupling with the continuum. In molecular spectra with more complex and overlapping signals, singular value decomposition and global fitting routines are widely used, as also described in the review of Beckwith et al.^218^ Pre-edge features can be analyzed by exponential fits that deconvolve the Gaussian response. More complex data sets with strongly overlapping excited state absorption over ground state depletion require singular value decomposition exclusively, as routinely used in valence electronic transient absorption spectroscopy.

Calculations of the transition energies and oscillator strengths of core electrons present a good test of the comparison of theory methods with experiment. Shifts of the order of 10 eV in TDDFT are not uncommon.^53,187^ Greater accuracy is achieved with the use of ADC (Algebraic Diagrammatic Construction) methods.^226,227^ In photochemical systems, the choice of the active space can provide a guided framework for theoretical methods and outcomes.^228^ Absolute agreement on a quantitative scale is hard and a distant dream, especially since near-edge transitions can be expected to have strong coupling effects with the ionization continuum. Nevertheless, solving electronic structure and electronic correlation effects with X-ray spectroscopy and theory is poised to be an important future problem for the deployment of quantum computers. We return to this point in the section on tackling emerging problems in catalysis and quantum information science.

## 8 Answering Key Questions in Molecular Spectroscopy

Here, we turn our attention to a brief review of some long-standing questions in molecular spectroscopy and the fate of molecular excited states with respect to ring opening, intersystem crossing, and bond dissociation where femtosecond X-ray transient absorption spectroscopy provided a cross-cutting “edge” in creating new mechanistic understanding from the vantage point of atomic sites. The reactions reviewed in this section are notable examples of non-Born–Oppenheimer processes that are central to determining the fate of molecular excited states. For example, measuring ring-opening dynamics is central to atmospheric chemistry, subcutaneous synthesis of vitamin-D upon exposure to sunlight, and molecular photoswitches. Intersystem crossing processes related to the fast crossing from singlet- to triplet state surfaces are critical to harnessing the full chemical potential of excited state processes pervasive in photoredox molecular systems. Before our study on acetylacetone,^53^ it was not known in the field that molecules composed of light atoms alone, such as carbon and oxygen, can participate in a singlet to triplet crossing on ultrafast time scales. This discovery was possible, thanks to the extension of the optically coupled region in the probing method, as we discuss in more detail below. Finally, chemical reactions with a branched population in the excited state are also very challenging to resolve, and tracking the radical species crucially informed the branching dynamics with ultrafast temporal resolution in bond dissociation reactions bearing more than one possibility for reactive outcomes in the excited state.

### Closed Ring or Open Chain?

The Woodward–Hoffmann rules have shaped our understanding of pericyclic reactions. However, the mixed electronic character in the vicinity of the hypothesized pericyclic minimum in the excited state required direct orbital sensitivity. This was made possible by X-ray spectroscopy through dipole-allowed 1s → 2p transitions at the carbon K-edge. This approach allowed the observation of a short-lived state with a 2π/1π* mixed electronic character in photoexcited 1,3-cyclohexadiene (CHD), with a lifetime of 110 ± 60 fs. Discrimination between ring-opened and ring-closed isomers in an experiment is very challenging because these species share closely similar electronic and vibrational spectra and the isomers have the same molecular mass. As noted by Ashfold and co-workers, “Experimental studies capable of revealing the dynamics of photoinduced ring-opening processes are still in their infancy...The challenges are substantial...Without subsequent collisional relaxation, measurement and assignment of spectra of the ring-opened species are likely to be challenging.”^229^

It can be seen from Figure 20a that the ground state, 1s → π* resonance in CHD is obtained at 284.5 eV.^187^ The next higher energy transition is the 1s → σ* resonance identified at 288.2 eV, demonstrating the direct orbital sensitivity of the technique. In comparison, the ground state, 1s → π* resonance in furfural is split into two peaks (Figure 20b), with the main peak at 286.4 eV and a shoulder at 285.1 eV.^228^ The presence of electronegative oxygen atoms increases the binding energy of the core-1s electron in carbon atoms, which shifts the resonance considerably higher than that of the hydrocarbon CHD. This spectrum demonstrates the sensitivity to chemical environments in X-ray spectroscopy via chemical shifts.

**Figure 20:**
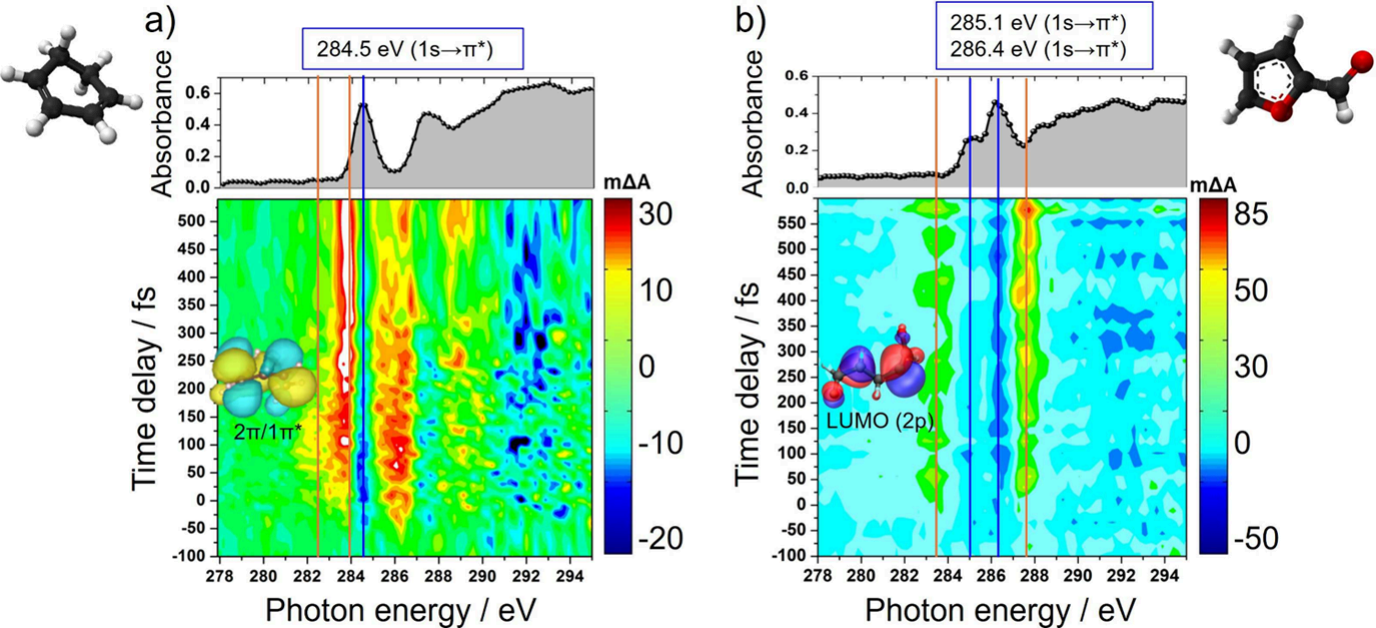
Ring opening in (a) 1,3-cyclohexadiene (CHD) and (b) furfural (FFR) initiated at 266 nm and probed at the carbon K-edge over a few hundred femtoseconds. The top trace shows the static absorption spectra of the molecules whereas the bottom trace shows the soft X-ray, transient absorption spectra. Modified from ref 187 Copyright 2017 AAAS and adapted from ref 28 and ref 228. Copyright 2018 American Chemical Society.

In CHD, a delayed rise of the intermediate is observed with respect to the ground-state depletion, which is the wave packet traversal time to the pericyclic minimum configuration. This delayed rise could be observed as the Franck–Condon region is unambiguously tracked in time-resolved X-ray spectroscopy because of the absence of any coherent artifacts near time zero where pump and probe pulses overlap. The spectrum of the 1,3,5-hexatriene (HT) product is not far from the ground-state CHD resonance due to the similarity of the electronic structures of the reactant and the product. We discuss this issue in more detail in the next section, where other complementary techniques have shown better sensitivity to changes in the nuclear structures of closed-shell, open- and closed-ring isomers.

In the case of ring-opening in furfural, a carbene intermediate is identified from a strongly dipole-allowed 1s → 2p transition at 283.8 eV. The intermediate is clocked on a 350 fs time scale from the dissociation of the C(1)-O bond, which gives rise to a carbon-centered radical. The carbene radicals with a nonbonding 2p unpaired electron light up at the carbon K-edge from the favorable matrix elements in the Fermi’s golden rule expression, making it a valuable probe to discriminate between closed-ring and open product species in this system. The case of the more subtle ring-puckering conical intersection geometry in furfural is a lot harder to detect unambiguously in X-ray spectroscopy, and we return to complementary probes of ring puckering in the next section. However, by tracking the recovery of ground state depletion in furfural, a ground state recovery of ≤ 15% could be inferred and represented an important contribution to the literature in the field that tends to be murky with respect to the extent of product formation versus ground state recovery in quantitative terms.

### Singlet or Triplet?

Acetylacetone (acac) is a notorious chameleon of all small organic molecules: in a bottle, it exists in the liquid state in the diketo form; however, as a vapor in the gas phase, it adopts the enol conformation. As a small organic molecule, it has one of the highest known absorption cross sections (4 × 10^–17^ cm^2^ molecule^–1^) in the ultraviolet; however, it displays very weak to no fluorescence, indicating ultrafast nonadiabatic dynamics at play. Photoexcitation from the ground state in acac leads to the population of the S_2_ (ππ*) excited state. Seminal ultrafast time-resolved studies from the Zewail and Mestdagh groups had indicated ultrafast internal conversion (S_2_ → S_1_) on a 2 ps time scale, followed by the formation of a triplet state on a 250 ps time scale. With access to a carbon K-edge probe and a strongly extended optically coupled region afforded by X-ray pulses at the carbon K-edge, we provided correct mechanistic understanding and new insight into the operation of an ultrafast (1.5 ± 0.2 ps) intersystem crossing process.

Our claim to revise the established photophysical paradigm in gas-phase acetylacetone was driven by the appearance of two consistent new features in the pre-edge region at 281.8 and 283.8 eV (Figure 21). In this region, we expected to see 1s → π resonances which would not make sense to assign to the S_1_ state known to be nπ* in electronic character. Here, *n* denotes a nonbonding 2p orbital on the oxygen atom. Thanks to early electronic structure CASSCF calculations available on acetylacetone,^231^ the experimental basis for assignment is derived from the foundational El Sayed’s rule in organic photochemistry. This rule states that a unit change in the electron spin angular momentum must be accompanied by a unit change in the electron orbital angular momentum. The TDDFT calculations of selected stationary points from the early CASSCF calculations validated the experimental assignment. For example, the calculations showed the 1sπ region of transient states to have a considerable oscillator strength, mainly for states that are ππ* in electronic character instead of nπ*, *i.e.*, the S_2_ and T_1_ states. In particular, the experimental results facilitated the development of an equation-of-motion coupled-cluster singles and doubles approach toward computing X-ray transient absorption spectra of triplet excited states (Figure 22).^230^ The computed signatures were found to be in good qualitative agreement, supporting the photophysical model for the excited state decay process.

**Figure 21:**
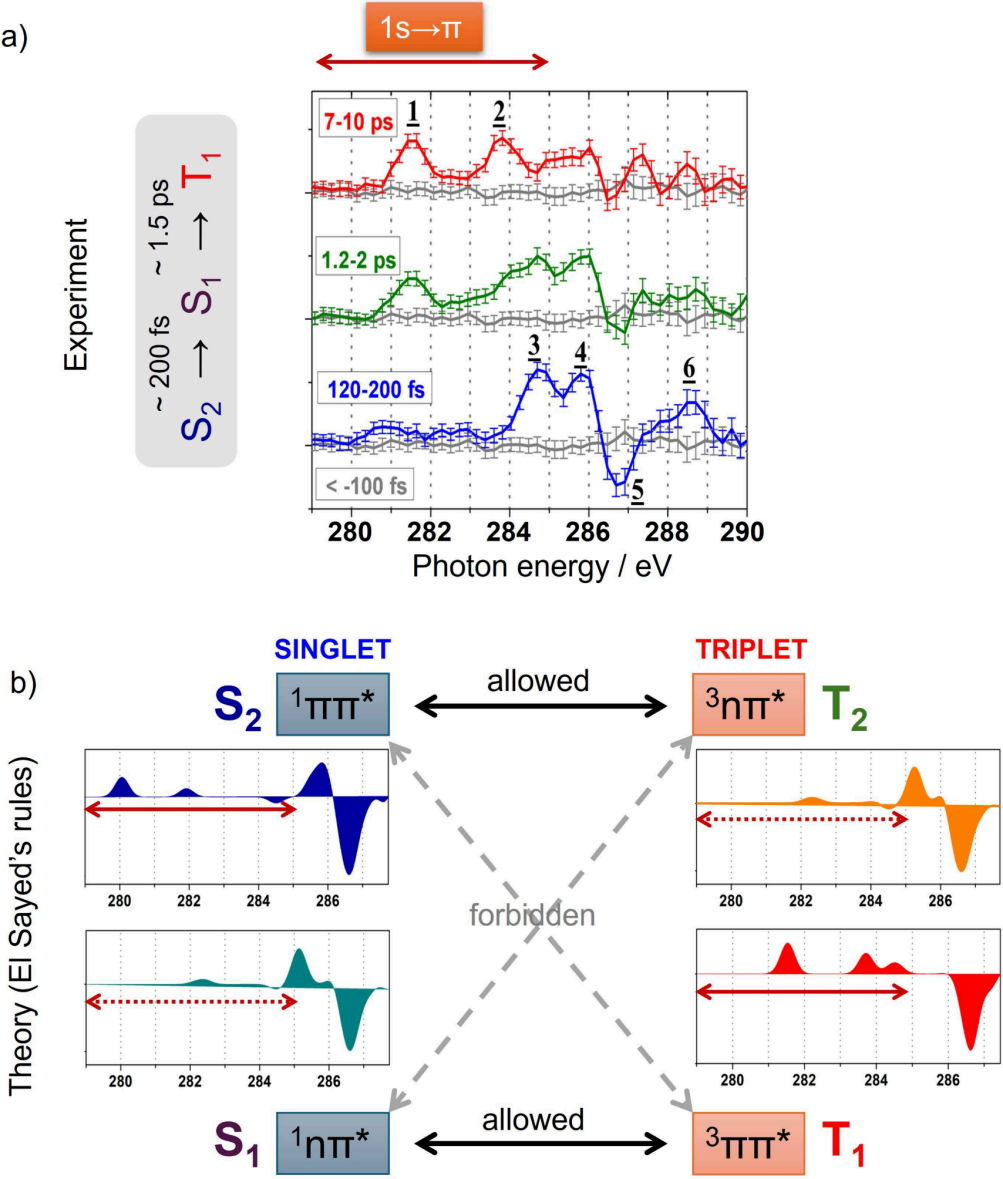
(a) Experiment and (b) theory of the singlet to triplet photochemical conversion in gas-phase acetylacetone. Adapted with permission from ref 53. Copyright 2017 American Chemical Society. The electronic character of the implicated electronic states and the underlying El Sayed’s predictions are denoted as described in the text.

**Figure 22:**
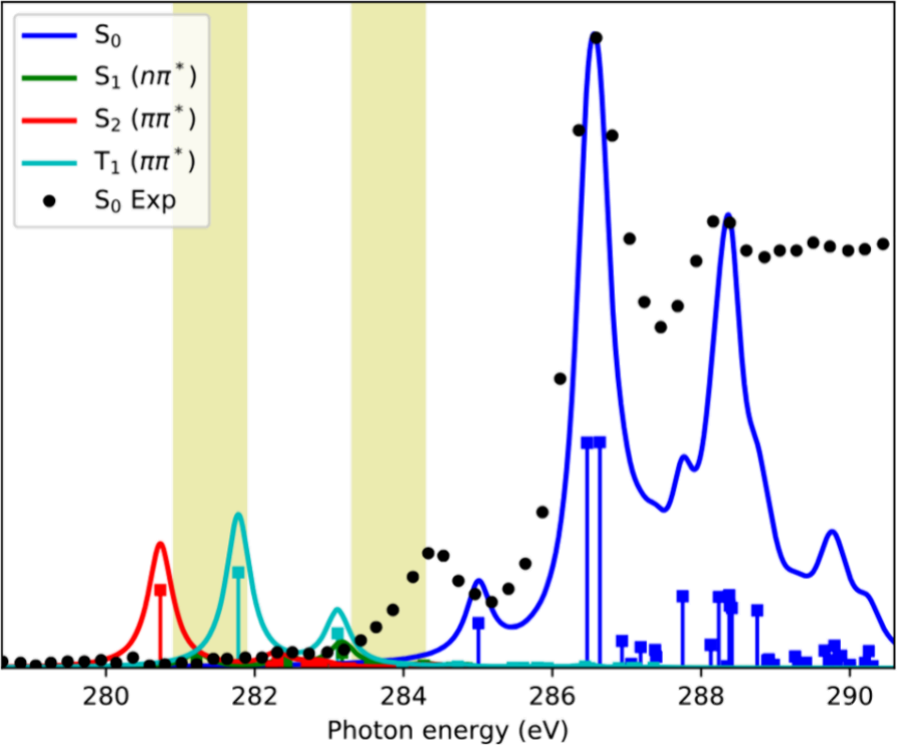
Excited state X-ray absorption spectra (CVS-CCSD/6–311++G** level of theory) of acetylacetone (acac) computed at the stationary point geometry of the ground and excited states indicated. Reprinted from ref 230 with the permission of AIP Publishing, Copyright 2019. Yellow columns in the figure indicate the location of the experimental X-ray transient absorption peaks for the triplet state in acac, and black dots indicate the ground-state static absorption spectrum reported by one of us.^53^

### One Way or the Other: The Case of Branching

Reactions in atmospheric chemistry and combustion processes are centered on volatile organic compounds. These research communities share a common goal for determinations of branched populations and radical intermediates, as accurate experimental measurements of these quantities can advance theoretical modeling efforts. In this context, sulfur compounds are a known contaminant in fossil fuels to the extent that processing fossil fuels and byproducts is based on robust desulfurization techniques. This inspired us to study the case of S–S versus C–S bond dissociation in dimethyl disulfide (DMDS), a model organic compound with carbon and sulfur atoms. The electric-dipole-allowed 2s → 3p transition enabled the observation of the methyl thiyl radical as the main product in the ultrafast dissociation of DMDS (Figure 18a). The sulfur L_1_ edge was critical in tracking this elusive radical intermediate, as a 2p → 3p transition at the sulfur L_2,3_ edge would be dipole forbidden. The carbon K-edge spectrum simultaneously evidenced the formation of a carbon-centered radical through a dipole-allowed 1s → 2p transition. A 1s → 2p transition at the carbon K-edge formally ascribed to the methyl radical was seen to be a minor product via an alternative dissociation channel in the reaction. The dissociation of alkyl halides with one or more than one type of halogen has also been utilized consistently to investigate the radical frontier orbitals (Figure 18b).^70,73^

Remarkably, for the case of dissociation of the axial bond in a metal porphyrin, the use of polarization was crucial to investigate the dynamics of the axial versus equatorial bonds.^232^ Traditionally, the use of polarized X-ray pulses in NEXAFS or XANES had been used to determine the orientation of small molecules on surfaces. Miller et al. have innovated with this concept to distinguish between axial and equatorial modes when applied to a transition metal complex with suitable optical transition moments. Similarly, Zhang et al. have shown vibrational coherence in an iron phenanthroline complex during the ultrafast spin crossover process.^60^ These works are notable not only because they successfully detected the dark intermediate states involved in electronic relaxation, but also because participant modes could be precisely determined. These outcomes are aligned with a central goal in the field of physical inorganic chemistry which is to investigate the significance and implications of quantum mechanical coherences in nonadiabatic excited state relaxation.^233^

## 9 Building a Systems Understanding

The shortcoming of X-ray absorption spectroscopy when applied to molecules is its (in)­ability to distinguish between nuclear structural isomers. Therefore, a dual- or multipronged experimental approach is necessary in many cases to reveal spectroscopy and dynamics at a level where we can construct mechanisms to build predictive theoretical models. Not only would it be an exercise in vain to push one experimental methodology to answer all possible questions about non-Born–Oppenheimer dynamics in a system, but we also run the risk of a skewed understanding of the underlying photophysics. A comprehensive, systems understanding is used to mean a holistic approach to reveal a complex functional molecule or material by probing its fundamental building blocks. Such an approach entails interrogating different intrinsic atoms and bonds within the system of interest, including the use of extrinsic reporter atoms that can collectively inform detailed mechanistic models of the structure, dynamics, and their interplay.

The outcome of a systems approach is often a mechanistic understanding of the factors that govern interactions, dynamics, and nonequilibrium behavior under different initial conditions. Therefore, a systems approach is powerful in providing a complete understanding by putting together the results of different experiments for accurate modeling and the development of predictive theories. Several beamline facilities are now pushing technological frontiers with bright photon and electron sources that encompass spectroscopy, scattering, and diffraction to generate systems understanding of molecular processes and ultrafast phenomena. We list below some pitfalls or blind spots of X-ray absorption spectroscopy as a probe and highlight other research methodologies that afford a complementary viewpoint.

### Structural Isomers

We present this discussion through three illustrative examples, based on the same molecules discussed in the previous section: (i) enol and dione acetylacetone (ii) cis and trans-furfural (iii) CHD and HT (Figure 23a−d). The first occurrence of a core-to-valence 1s → π*­(LUMO) transition in the diketo form of acac is expected to occur at 286.5 eV, which strongly overlaps with the enol tautomer. Therefore, it is not possible to extract a ratio of enol to keto forms in the gas phase without resorting to complementary vibrational spectroscopy. The same can be said for the 1sπ*­(LUMO) resonance in the cis and trans isomers of furfural, as well as in the closed-ring reactant and the open-chain product in the conversion of 1,3-cyclohexadiene to 1,3,5-hexatriene, where the spectra of structural isomers are strongly overlapping and virtually indistinguishable. In this latter work, a nonadiabatic MD simulation was necessary to investigate the effect of vibrational excitation on the oscillator strength for core-to-valence 1sπ* resonances in the reactant and product. These calculations showed that vibrationally excited hexatriene has a higher 1s → π*­(LUMO) oscillator strength than vibrationally excited CHD, from which an 80:20 branching fraction was inferred for the product and ground-state recovery channels. The ambiguity of structural isomers is also transferred to materials such as TiO_2_ that can exist as rutile, anatase, or amorphous with very similar peak positions and oscillator strengths in the near edge (Figure 23e).^234^ These limitations in X-ray absorption spectroscopy are also well recognized in structural elucidations of metal atoms such as zinc in proteins^235^ and investigations of *in situ* or operando electrochemistry.^236^ In fact, spectral assignment in the near-edge to a mixture of isomers is an emerging problem in X-ray absorption spectroscopy poised for quantum computers to tackle in the future, as we highlight in the next section.

**Figure 23:**
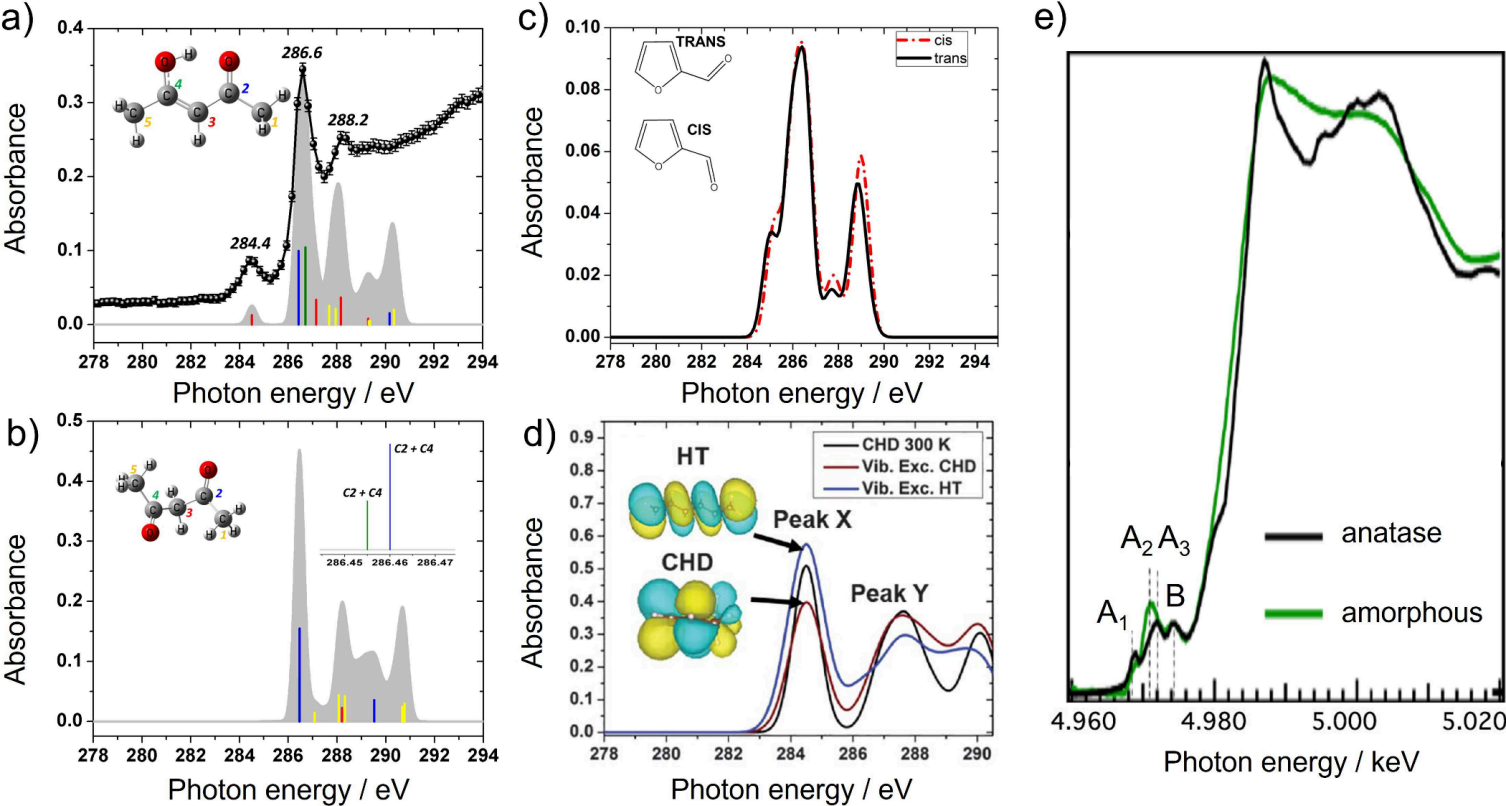
NEXAFS spectra of the structural isomers of (a,b) acetylacetone, enol and diketo form, reproduced with permission from ref 53. Copyright 2017 American Chemical Society, (c) furfural, cis and trans forms, reproduced with permission from ref 228. Copyright 2018 American Chemical Society, (d) 1,3-cyclohexadiene and 1,3,5-hexatriene including vibrational excitation, reproduced with permission from ref 187 Copyright 2017 AAAS, and (e) XANES spectrum of anatase and amorphous forms of TiO_2_, reprinted from ref 234 with the permission of AIP Publishing, Copyright 2016.

### Branched Populations

Ring opening reactions present a prominent example of branching in the molecular phase space. Because X-ray transient absorption spectroscopy is an implementation of a Lambert–Beer-type of measurement with respect to the X-ray probe, the transient X-ray absorption spectra reflect the branched intermediates and products. As discussed above, structural isomers with overlapping X-ray absorption spectra can be difficult to reveal unambiguously. For example, ring opening versus ring puckering in furfural (Figure 24) can both lead to a repopulation of the ground state; however, the method does not have the sensitivity to follow the conical intersection regions (Cl_*O*_ and Cl_*P*_) unambiguously unless both the carbon K-edge and the oxygen K-edge are investigated. Generally, recovery of the parent ground state depletion is used to track the relative branching ratio in photochemical reactions. Additionally, singlet versus triplet state determinations in excited state photochemistry are also difficult to quantify, as X-ray oscillator strengths can vary significantly from one theory method to another, and the amount of vibrational broadening from electronic relaxation also plays a role. Therefore, systematic investigations through multipronged experimental approaches are desirable.

**Figure 24:**
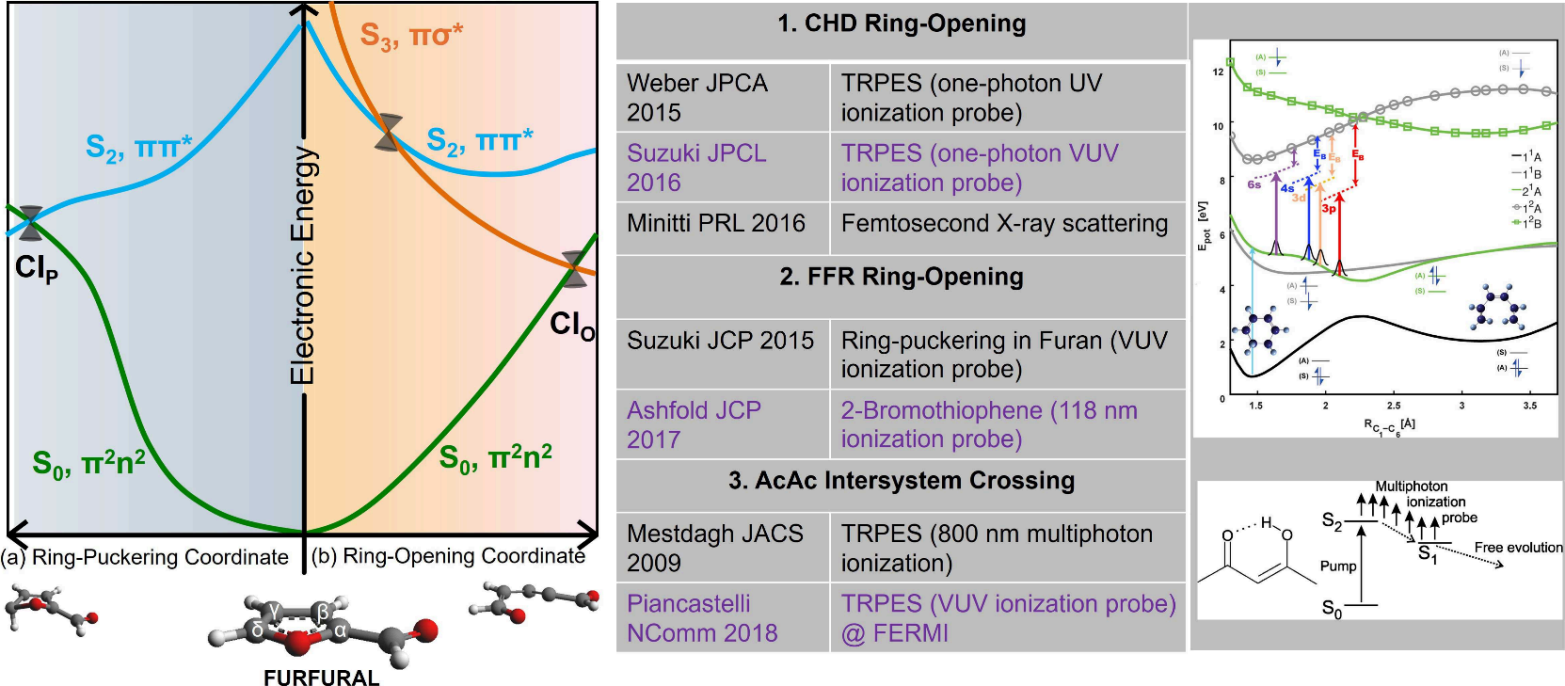
Understanding of chemical reaction mechanisms with multiple reaction outcomes requires a systems approach. In this approach, it should be possible to rationalize the observations of multiple experiments for a given system and initial conditions in a self-consistent manner with regard to temporal evolution and experimentally determined rate constants. Adapted with permission from ref 228, Copyright 2018; ref 237, Copyright 2015; and ref 238, Copyright 2008 American Chemical Society.

One such approach that has been successful is to launch valence ionization through a Rydberg state manifold as a probe (Figure 24). This approach allowed the investigation of dark states involved in CHD to HT photoconversion at 266 nm.^237^ Similarly, time-resolved photoelectron spectroscopy work using XUV pulses reported the detailed nonadiabatic reaction pathway through the doubly excited state, revealing vibrational coherence in the product state.^239^ Studies of ring opening using tabletop sources with isolated attosecond pulses have provided access to conical intersections with a high temporal resolution.^240^ Remarkably, time-resolved scattering using hard X-rays and mega-electronvolt electron beams have shown ring opening with sensitivity to product isomers and ring puckering with sensitivity to hydrogen-out-of-plane.^241,242^ Figure 24 summarizes few other experimental techniques that have enabled a truly systems understanding of complex wave packet dynamics in the molecular systems of cyclohexadiene, furfural, acetylacetone, and related analogs.^238,243–247^

### Measurement Sensitivity

Sensitivity in X-ray transient absorption measurements is much too low (milli-OD) in comparison to charged particle detection, such as ions and photoelectrons (example, particle detection with microchannel plates and channeltrons can be one in ten thousand). This arises because transient absorption is measured against a finite background, whereas emission or photoelectron-based probes are ideally measured against a zero background, providing higher sensitivity. Therefore, the pump power has to be kept relatively high to measure the excited-state dynamics in a reasonable time frame. It is customary to check the dependence of the pump power over a decade of pump power levels to ensure the probing of single-photon excitation events.^53,228^ In ponderomotive shifting IRF measurements, signal strength of 6–7 mOD is measured and it is quite challenging to obtain stable signal levels with SNR of 2 or higher. In comparison, XPS detection for membrane-based gas separations showed exquisite sensitivity with respect to measuring Stark shifts of the trapped argon atoms in a photoexcited carbon nitride membrane.^248^ The limits on measurement sensitivity and detection for XAS as a bulk spectroscopic technique are also recognized with respect to speciation in biological samples or fractions of active sites in transition metal catalysts.^249–251^

## 10 Tackling Emerging Problems

As X-ray spectroscopy has rapidly developed into a mature technique, the possibilities for future applications are numerous and exciting. The elemental sensitivity and ultrafast resolution can be used to interrogate vast regions of the molecular phase space in small molecules and materials relevant for catalysis and quantum information science. As the potential energy landscape straddles 3–6 eV for valence electronic excitation and 8–10 eV for valence ionization, valence electron dynamics is comprehensively covered within the HHG bandwidth, which bodes well for emerging problems in catalysis, energy conversion, quantum sensing, and quantum information science.

### Energy Conversion and Catalysis

The catalytic oxidation of CO on the surface of a 400 nm photoexcited ruthenium substrate was studied by femtosecond X-ray pulses at a free electron laser facility.^252,253^ The study revealed the transition state region of this surface chemical reaction by probing the valence electronic structure of adsorbed CO molecules and O atoms at the oxygen K-edge. The appearance of new electronic states on the order of 800 fs evidenced the formation of new adsorbed species with a substantially elongated C–O bond compared to the CO_2_ final product. Comparison to a quantum oscillator model showed a 10% population in the transition state region within the first few picoseconds of the optical pump pulse. The excitation of electrons in the substrate and subsequent energy transfer to the adsorbate caused significant vibrational excitation of the adsorbate–substrate bonds. Historically, X-ray spectroscopy was developed for the study of molecules deposited on surfaces where the polarization of the electric field can be altered to study the parallel and perpendicular components of the molecular dipole (Figure 25b).

**Figure 25:**
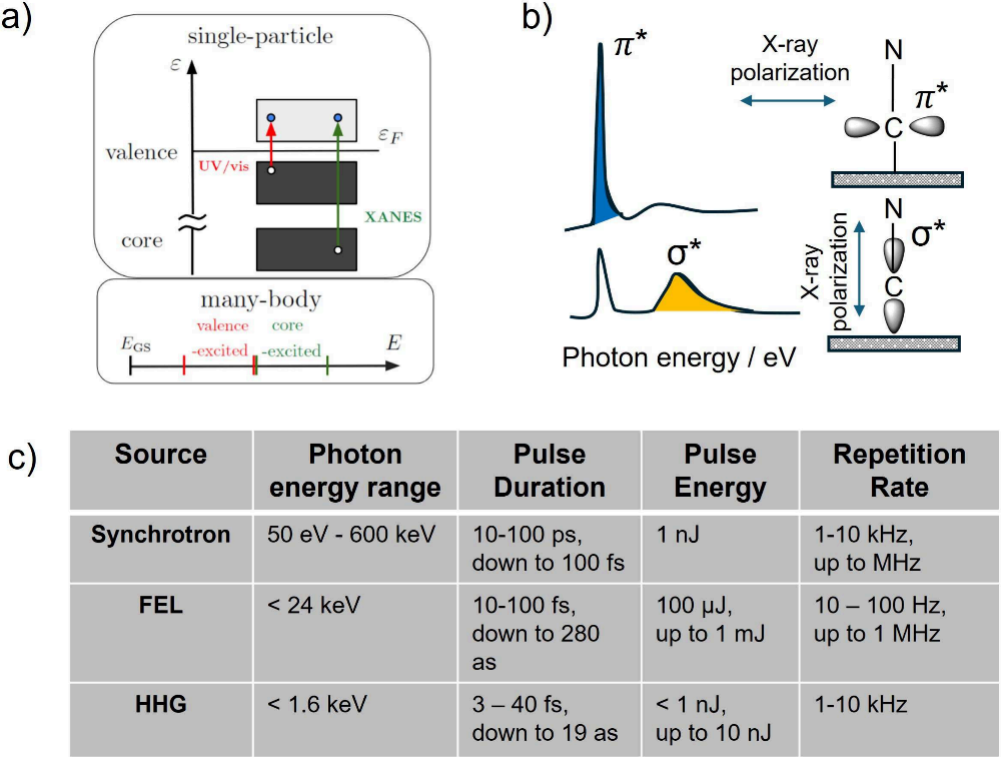
(a) Valence and core-excited states are both relevant for simulating energy (battery) materials on a quantum computer. Reprinted with permission from ref 257. Copyright 2025, Fomichev et al. (b) Cartoon depiction of the sensitivity of 1sπ* and 1sσ* resonances to the X-ray polarization. (c) Roadmap of available short-pulse X-ray light sources to tackle emerging problems in energy conversion, catalysis, and quantum information science.

More recently, the interaction of transition metal catalysts with alkanes has been studied using femtosecond X-ray transient absorption spectroscopy in a liquid jet.^254^ This study, also conducted at a free electron laser (time scales of up to 10 ps) and a synchrotron facility (time scales of up to 25 ns), tracked the frontier orbitals through the metal (rhodium-L_3_) X-ray absorption edge. Electron donation and back-donation effects underlying metal-alkane bonds and metal–carbonyl bonds crucially informed charge-transfer interactions and C–H activation mechanisms using a cyclopentadienyl rhodium carbonyl complex. Together, these experiments are informative for the structures and reactivity of small molecules in a solvent medium or on a surface as the starting reactant molecules are being transformed into value added chemicals. On slower time scales of up to minutes, X-ray spectroscopy coupled with scanning tunneling microscopy has revealed ethylene polymerization mechanisms on a surface.^255^

In uncharted territory, some of the first problems quantum computers will solve in the future are likely to be in the domain of energy materials.^256,257^ As an example, studying the mechanisms of charge migration, energy storage, and material degradation is a systems-level problem with several interconnected research hypotheses. The detailed analysis of mechanistic aspects can focus on a systematic investigation of oxidation states, spin states, or both, where existing theory methods often need to strike a balance between computational runtime and accuracy. Increasing accessibility to spectroscopy data on tabletop experimental HHG sources will provide easy access benchmarks and experimental pivots to refine method development in electronic structure theories. Direct time-resolved measurements of energy gaps and orbital mixing coefficients in different charge transfer excited states on tabletop setups are expected to significantly assist theoretical methods development as these fundamental excited state properties are almost exclusively obtained from electronic structure calculations. Note that 1s K-edge absorption spectroscopy is a well-established method in determining bond covalency and orbital mixing coefficients in diverse systems ranging from transition metal complexes containing metal-chlorine and metal–carbon bonds^258,259^ to the hydrated electron.^260^ Therefore, transient changes in X-ray spectra at multiple absorption edges will enable tracking electron density distributions at localized centers with sensitivity to orbitals, allowing the quantification of mixing of a central metal atom’s *d*-orbitals with the ligand (carbon or halogen) *p*-orbitals in targeted photoexcited states.

### Quantum Information Science

There are many ways to drive a quantum system out of equilibrium, prompting us to return to Figure 1b to emphasize the coherences and quantum mechanical couplings accompanying non-Born–Oppenheimer dynamics. These dynamics are also tractable in the domain of several emerging problems, potentially cross-cutting multiple disciplines. Among DiVincenzo’s five criteria for the physical implementation of quantum computing, (i) a scalable physical system with well-characterized qubits, together with (ii) long relevant decoherence times, much longer than the gate operation time, are central features not necessarily straightforward to address by ultrafast spectroscopy, let alone ultrafast X-ray absorption spectroscopy.^261^ In fact, to this end, it has been articulated that the quantum optics and spectroscopy communities normally have opposite goals.^262^ As a pathway to bridge this divide and potentially innovate at this cross-disciplinary intersection, the atomic and orbital sensitivity of X-ray absorption edges can help parametrize the vast space of quantum superposition and entanglement with regards to both efficiency and fidelity.

Quantum optics in the X-ray regime has recently been considered to address the potential of quantum computing using free electron laser and synchrotron sources.^263^ Platforms such as molecules, semiconductor quantum dots, 2D materials, defects-based single photon emitters, and nitrogen-vacancy centers in diamond are active areas of interest. As the development of quantum technology will rely on noise resistant quantum materials, measurements will need to probe joint entanglement of charge, spin, orbital, and lattice degrees of freedom as shown in Figure 1b. Generating reliable quantum-mechanical descriptions of single particle and many-body effects is possible using this type of spectroscopy (Figure 25a). The use of polarization (linear parallel, linear perpendicular, Figure 25b) as well as circular polarization can spectrally profile these properties at various orientations and depths of the quantum material. The possibility of investigating heteroatom vacancies and defect centers in carbon-based materials can rapidly assist in the search for new quantum materials and molecular qubits.^264^ Signal to noise ratios realizable with tabletop XUV sources indicate that defect densities at a few percent level can be measured and further developments outline a promising direction for X-ray transient absorption spectroscopy.^265–267^

Quantum sensing has been largely focused on materials design, usually ignoring the interaction of small molecules in photoexcited systems. Theoretical work on X-ray Stark spectroscopy which tracks the Stark shifting of Rydberg states in small molecule prototypes in the presence of an external electric field has revealed the scaling behavior of excitations to valence (linear scaling) and Rydberg orbitals (quadratic scaling).^268^ An in-depth understanding of such scaling and its rationalization with predictive models will serve well for applications in separations and quantum sensing. The ability to probe defect centers in quantum materials in a site-specific manner as well as spectroscopy of trapped gas atoms in layered materials are important future prospects.^248,269,270^ On a technical front, the extension of the HHG cutoff energies with bright-squeezed vacuum^271^ and XUV generation with circularly polarized pulses^272^ will open doors to study the influence of chirality on non-Born–Oppenheimer processes and dynamics.

## 11 Outlook and Concluding Remarks

This tutorial review introduces ultrafast XUV spectroscopy as a powerful tool for probing coupled electronic and nuclear dynamics in molecules, particularly in regimes where the Born–Oppenheimer approximation breaks down and optical probes lack sufficient specificity. Benchmark studies in molecular spectroscopy and nonadiabatic dynamics are contextualized with recent and emerging applications of this relatively new form of spectroscopy. The generation of extreme ultraviolet and soft X-ray pulses on a tabletop source is discussed with the underlying theory and motivation. In Section 2, we frame molecular dynamics in terms of motion on potential energy surfaces and the concept of an “optically coupled region” to establish how wave packets are prepared and detected. Comparisons between valence and core-level probes in Section 3 highlight how element specificity, local orbital sensitivity, and spin–orbit effects fundamentally shape the information content of time-resolved measurements at characteristic atomic edges.

A more technical description of how ultrafast XUV pulses are generated and characterized using tabletop high-harmonic generation is addressed in Sections 4 and 5, followed by a practical overview of typical experimental apparatus (Section 6) and data analysis methods (Section 7). These tools are connected to key chemical and physical questions that XUV spectroscopy can address, discussed in Section 8, including tracking electronic populations, identifying transient intermediates, and disentangling competing reaction pathways. Varied and specific cases of the failure of the Born–Oppenheimer approximation are discussed where the electronic relaxation couples significantly with nuclear motions. The ability to detect atomic and radical fragment products of ultrafast chemical reactions is a distinct advantage as the method has direct sensitivity to the character of the frontier molecular orbitals.

The sensitivity to spin states, although not formally allowed in electric-dipole transitions in closed-shell molecules, is indirectly captured through the conservation of total angular momentum, requiring a unit change in the orbital angular momentum where the electron is expected to move from nonbonding (*n*) to π orbitals, as an example. Finally, we emphasize the importance of integrating experiment and theory to build a systems-level understanding of molecular dynamics (Section 9), further outlining emerging challenges and opportunities in X-ray spectroscopy when applied to increasingly complex systems (Section 10). Overall, we expect that this powerful method will continue to be developed for exploring the nooks and corners of potential energy surfaces in complex functional matter to improve our observation, understanding, and control of chemical reactivity at unprecedented time scales.

## Supplementary Material



## Notes

Schematics for Born–Oppenheimer approximation, exchange splitting and K-shell spectroscopy of molecular oxygen, tables with descriptions of equations and parameters used in the main text (PDF)

## Vocabulary

Strong-Field Approximation: in a sufficiently intense laser field, an electron is promoted from the ground-state directly to continuum states, surpassing all bound/resonant-states, and propagates freely in a laser field ignoring the Coulomb potential of the nucleus.
Keldysh Theory: describes how electrons are ionized in strong laser-fields and is classified by the dimensionless Keldysh Parameter, γ, which compares the tunneling time to the period of the optical field, $\gamma =\frac{\omega_{0}\sqrt{2{\mathrm{mI}}_{p}}}{\mathrm{eE}}=\sqrt{\frac{\omega_{0}^{2}2{\mathrm{mI}}_{p}}{e^{2}E^{2}}}=\sqrt{\frac{I_{p}}{2U_{p}}}$ . This delineates when ionization occurs by multiphoton excitation γ > > 1, or by tunnel ionization γ < < 1.
Ponderomotive Energy: is the time-averaged kinetic energy of an electron in an oscillating field and is given by $U_{p}=\frac{{\left(\mathrm{eE}\right)}^{2}}{4m\omega_{0}^{2}}$ .
High-Harmonic Generation: is a process by which high-order harmonics of an intense laser pulse are generated by focusing the pulse into a medium such as a noble gas.
Phase Matching: describes considerations made to ensure that the wave vector of the generated harmonics matches that of the driving laser, *Δk* = 0, such that the harmonics are generated constructively.
Cutoff Energy: marks the theoretical maximum energy produced by high harmonic generation and is given by ℏω = *I*_*p*_ + 3.17*U*_*p*_, where *I*_*p*_ is the ionization potential of the generating medium and *U*_*p*_ is the ponderomotive energy (see above).
Nonadiabatic Coupling: refers to the coupling between electronic and nuclear motion and is paramount in understanding the non-Born–Oppenheimer dynamics considered in the electronically excited state.
Rydberg States: are electronically excited states that follow the Rydberg formula and thus converge toward an ionization state.
Molecular Phase Space: refers to the multidimensional space that completely defines a molecular system, taking the coordinates of position and momentum.
Franck–Condon Region: describes the region in phase space in which an electronic transition is completely vertical, leaving the nuclear coordinates unchanged.

## References

[1] Born, M. ; Oppenheimer, R. Quantum Chemistry: Classic Scientific Papers; World Scientific: 2000; pp 1–24.

[2] Butler L. J. (1998). Chemical
reaction dynamics beyond the Born-Oppenheimer approximation. Annu. Rev. Phys. Chem..

[3] Schuurman M. S., Stolow A. (2018). Dynamics at conical
intersections. Annu. Rev. Phys. Chem..

[4] Bubin S., Pavanello M., Tung W.-C., Sharkey K. L., Adamowicz L. (2013). Born–Oppenheimer
and non-Born–Oppenheimer, atomic and molecular calculations
with explicitly correlated Gaussians. Chem.
Rev..

[5] Scherrer A., Agostini F., Sebastiani D., Gross E., Vuilleumier R. (2017). On the mass
of atoms in molecules: Beyond the Born-Oppenheimer approximation. Physical Review X.

[6] Liu Q., Zewail A. H. (1993). Femtosecond real-time probing of reactions. 10. Reaction
times and model potentials. J. Phys. Chem..

[7] Neville S. P., Chergui M., Stolow A., Schuurman M. S. (2018). Ultrafast
x-ray spectroscopy of conical intersections. Phys. Rev. Lett..

[8] Turro, N. J. Modern molecular photochemistry; University science books: 1991.

[9] Sun X., James T. D., Anslyn E. V. (2018). Arresting “loose bolt”
internal conversion from- B (OH) 2 groups is the mechanism for emission
turn-on in ortho-aminomethylphenylboronic acid-based saccharide sensors. J. Am. Chem. Soc..

[10] Zhai J., Ai Q., Li H., Liu Z., Hu X. (2025). Visible-Light-Driven
Fluorescence Turn-on Photoswitches With Near Quantitative Photocyclization
Yield. Advanced Science.

[11] Zewail A. H. (1988). Laser femtochemistry. Science.

[12] Manthe U., Köppel H. (1990). Dynamics on potential energy surfaces
with a conical
intersection: Adiabatic, intermediate, and diabatic behavior. J. Chem. Phys..

[13] Brixner T., Gerber G. (2003). Quantum control of gas-phase and liquid-phase femtochemistry. ChemPhysChem.

[14] Nuernberger P., Vogt G., Brixner T., Gerber G. (2007). Femtosecond quantum
control of molecular dynamics in the condensed phase. Phys. Chem. Chem. Phys..

[15] Tully J. C. (2012). Perspective:
Nonadiabatic dynamics theory. J. Chem. Phys..

[16] Kammeraad J. A., Zimmerman P. M. (2016). Estimating
the derivative coupling vector using gradients. J. Phys. Chem. Lett..

[17] Nelson T. R., White A. J., Bjorgaard J. A., Sifain A. E., Zhang Y., Nebgen B., Fernandez-Alberti S., Mozyrsky D., Roitberg A. E., Tretiak S. (2020). Non-adiabatic excited-state
molecular dynamics: Theory
and applications for modeling photophysics in extended molecular materials. Chem. Rev..

[18] Rosker M. J., Dantus M., Zewail A. H. (1988). Femtosecond real-time
probing of
reactions. I. The technique. J. Chem. Phys..

[19] Rose T. S., Rosker M. J., Zewail A. H. (1989). Femtosecond
real-time probing of
reactions. IV. The reactions of alkali halides. J. Chem. Phys..

[20] Bernstein R. B., Zewail A. H. (1989). Femtosecond real-time probing of reactions. III. Inversion
to the potential from femtosecond transition-state spectroscopy experiments. J. Chem. Phys..

[21] Pedersen S., Herek J., Zewail A. (1994). The validity of the “diradical”
hypothesis: direct femtoscond studies of the transition-state structures. Science.

[22] Polanyi J. C., Zewail A. H. (1995). Direct observation of the transition
state. Acc. Chem. Res..

[23] Cheng P., Zhong D., Zewail A. H. (1996). Femtosecond
real-time probing of
reactions. XXI. Direct observation of transition-state dynamics and
structure in charge-transfer reactions. J. Chem.
Phys..

[24] Bhattacherjee A., Attar A. R., Leone S. R. (2016). Transition state region in the A-Band
photodissociation of allyl iodide—A femtosecond extreme ultraviolet
transient absorption study. J. Chem. Phys..

[25] Attar A. R., Bhattacherjee A., Leone S. R. (2015). Direct observation of the transition-state
region in the photodissociation of CH3I by femtosecond extreme ultraviolet
transient absorption spectroscopy. J. Phys.
Chem. Lett..

[26] Chergui M., Thomas J. M. (2017). From structure to structural dynamics: Ahmed Zewail’s
legacy. Structural Dynamics.

[27] Stolow A., Bragg A. E., Neumark D. M. (2004). Femtosecond
time-resolved photoelectron
spectroscopy. Chem. Rev..

[28] Bhattacherjee A., Leone S. R. (2018). Ultrafast X-ray
Transient Absorption Spectroscopy of
Gas-Phase Photochemical Reactions: A New Universal Probe of Photoinduced
Molecular Dynamics. Acc. Chem. Res..

[29] Agostini F., Curchod B. F. (2022). Chemistry without
the Born–Oppenheimer approximation. Philosophical
Transactions of the Royal Society A: Mathematical,
Physical and Engineering Sciences.

[30] Hammes-Schiffer S. (2022). Theoretical
perspectives on non-Born–Oppenheimer effects in chemistry. Philosophical Transactions of the Royal Society A: Mathematical,
Physical and Engineering Sciences.

[31] Chachisvilis M., Zewail A. H. (1999). Femtosecond dynamics
of pyridine in the condensed phase:
Valence isomerization by conical intersections. J. Phys. Chem. A.

[32] Polli D., Altoè P., Weingart O., Spillane K. M., Manzoni C., Brida D., Tomasello G., Orlandi G., Kukura P., Mathies R. A. (2010). Conical intersection dynamics of the primary
photoisomerization event in vision. Nature.

[33] DeBeer
George S., Brant P., Solomon E. I. (2005). Metal and ligand
K-Edge XAS of organotitanium complexes: Metal 4p and 3d contributions
to pre-edge intensity and their contributions to bonding. J. Am. Chem. Soc..

[34] Atkins A. J., Bauer M., Jacob C. R. (2013). The chemical sensitivity of X-ray
spectroscopy: high energy resolution XANES versus X-ray emission spectroscopy
of substituted ferrocenes. Phys. Chem. Chem.
Phys..

[35] Seres, E. ; Spielmann, C. In Femtosecond-Scale Optics; Andreev, A. V. , Ed.; IntechOpen: 2011.

[36] Bressler C., Chergui M. (2004). Ultrafast x-ray absorption
spectroscopy. Chem. Rev..

[37] Stöhr, J. NEXAFS Spectroscopy; Springer Berlin Heidelberg: 1992; Vol. 25.

[38] Thomas A., Fischer A., Goettmann F., Antonietti M., Müller J.-O., Schlögl R., Carlsson J. M. (2008). Graphitic carbon
nitride materials: variation of structure and morphology and their
use as metal-free catalysts. J. Mater. Chem..

[39] Ohrwall G., Ottosson N., Pokapanich W., Legendre S., Svensson S., Bjorneholm O. (2010). Charge dependence
of solvent-mediated intermolecular
Coster- Kronig decay dynamics of aqueous ions. J. Phys. Chem. B.

[40] Hikosaka Y., Lablanquie P., Kaneyasu T., Adachi J., Tanaka H., Suzuki I., Ishikawa M., Odagiri T. (2021). Super-Coster-Kronig
decay of Kr 3 p core-hole states studied by multielectron coincidence
spectroscopy. Phys. Rev. A.

[41] Hikosaka Y., Fritzsche S. (2022). Coster-Kronig
and super Coster–Kronig transitions
from the Xe 4s core–hole state. Phys.
Chem. Chem. Phys..

[42] Gaffney K. J. (2021). Capturing
photochemical and photophysical transformations in iron complexes
with ultrafast X-ray spectroscopy and scattering. Chem. Sci..

[43] Gessner O., Guhr M. (2016). Monitoring ultrafast
chemical dynamics by time-domain x-ray photo-and
auger-electron spectroscopy. Accounts of Chemical
Research.

[44] Mayer D., Lever F., Gühr M. (2024). Time-resolved x-ray spectroscopy
of nucleobases and their thionated analogs. Photochemistry and Photobiology.

[45] Griffiths, D. J. ; Schroeter, D. F. Introduction to quantum mechanics; Cambridge university press: 2018.

[46] De
Groot F. (2005). Multiplet effects in X-ray spectroscopy. Coord.
Chem. Rev..

[47] De
Groot F. (2001). High-resolution X-ray emission and X-ray absorption spectroscopy. Chem. Rev..

[48] De
Groot F. (2022). Multiplet approaches in X-ray absorption spectroscopy. International Tables for Crystallography.

[49] Plaschke M., Rothe J., Altmaier M., Denecke M. A., Fanghänel T. (2005). Near edge
X-ray absorption fine structure (NEXAFS) of model compounds for the
humic acid/actinide ion interaction. Journal
of Electron Spectroscopy and Related Phenomena.

[50] Schultze M., Ramasesha K., Pemmaraju C., Sato S., Whitmore D., Gandman A., Prell J. S., Borja L., Prendergast D., Yabana K. (2014). Attosecond band-gap dynamics in silicon. Science.

[51] Bagus P. S., Illas F., Pacchioni G., Parmigiani F. (1999). Mechanisms
responsible for chemical shifts of core-level binding energies and
their relationship to chemical bonding. Journal
of Electron Spectroscopy and Related Phenomena.

[52] Shaw D., King G., Read F., Cvejanovic D. (1982). The observation
of electric-dipole-forbidden inner-shell transitions in N2 and Ar
by the electron energy-loss technique. Journal
of Physics B: Atomic and Molecular Physics.

[53] Bhattacherjee A., Pemmaraju C. D., Schnorr K., Attar A. R., Leone S. R. (2017). Ultrafast
intersystem crossing in acetylacetone via femtosecond X-ray transient
absorption at the carbon K-edge. J. Am. Chem.
Soc..

[54] Ma H., Peng Q., An Z., Huang W., Shuai Z. (2019). Efficient
and long-lived room-temperature organic phosphorescence: theoretical
descriptors for molecular designs. J. Am. Chem.
Soc..

[55] Mao Y., Yao X., Yu Z., An Z., Ma H. (2024). Ground-State Orbital
Descriptors for Accelerated Development of Organic Room-Temperature
Phosphorescent Materials. Angew. Chem..

[56] Glatzel P., Bergmann U. (2005). High resolution 1s
core hole X-ray spectroscopy in
3d transition metal complexes—electronic and structural information. Coordination Chemistry Reviews.

[57] Wolny J. A., Schünemann V., Németh Z., Vankó G. (2018). Spectroscopic
techniques to characterize the spin state: Vibrational, optical, Mössbauer,
NMR, and X-ray spectroscopy. Comptes Rendus
Chimie.

[58] Troß J., Carter-Fenk K., Cole-Filipiak N. C., Schrader P., Word M., McCaslin L. M., Head-Gordon M., Ramasesha K. (2023). Excited-state
dynamics during primary C–I homolysis in acetyl iodide revealed
by ultrafast core-level spectroscopy. J. Phys.
Chem. A.

[59] Carlson D. R., Bandaranayake S., Ramasesha K. (2026). Tabletop Core-to-Valence Transient
Absorption Spectroscopy of Ultrafast Gas-Phase Chemical Dynamics. Annu. Rev. Phys. Chem..

[60] Zhang K., Ash R., Girolami G. S., Vura-Weis J. (2019). Tracking the Metal-Centered Triplet
in Photoinduced Spin Crossover of Fe­(phen)_3_
^2+^ with Tabletop Femtosecond M-Edge X-ray Absorption Near-Edge Structure
Spectroscopy. J. Am. Chem. Soc..

[61] Ryland E. S., Zhang K., Vura-Weis J. (2019). Sub-100 fs
intersystem crossing to
a metal-centered triplet in Ni (II) OEP observed with M-edge XANES. J. Phys. Chem. A.

[62] Vura-Weis J. F. (2025). Extreme
Ultraviolet Absorption Spectroscopy of Transition Metal Complexes. Annu. Rev. Phys. Chem..

[63] Vankó G., Neisius T., Molnar G., Renz F., Karpati S., Shukla A., De Groot F. M. (2006). Probing
the 3d spin momentum with
X-ray emission spectroscopy: The case of molecular-spin transitions. J. Phys. Chem. B.

[64] Zhang W., Alonso-Mori R., Bergmann U., Bressler C., Chollet M., Galler A., Gawelda W., Hadt R. G., Hartsock R. W., Kroll T. (2014). Tracking excited-state charge and spin dynamics in
iron coordination complexes. Nature.

[65] Tatsuno H., Kjær K. S., Kunnus K., Harlang T. C., Timm C., Guo M., Chàbera P., Fredin L. A., Hartsock R. W., Reinhard M. E. (2020). Hot branching dynamics in a light-harvesting
iron carbene complex revealed by ultrafast X-ray emission spectroscopy. Angew. Chem..

[66] Kim Y., Nam D., Ma R., Kim S., Kim M.-j., Kim J., Eom I., Lee J. H., Kim T. K. (2022). Development of an experimental apparatus
to observe ultrafast phenomena by tender X-ray absorption spectroscopy
at PAL-XFEL. Journal of Synchrotron Radiation.

[67] Ding T., Rebholz M., Aufleger L., Hartmann M., Stooß V., Magunia A., Birk P., Borisova G. D., Wachs D., da Costa Castanheira C. (2021). Measuring the frequency chirp of extreme-ultraviolet
free-electron laser pulses by transient absorption spectroscopy. Nat. Commun..

[68] Grguraš I., Maier A. R., Behrens C., Mazza T., Kelly T., Radcliffe P., Düsterer S., Kazansky A., Kabachnik N., Tschentscher T. (2012). Ultrafast X-ray pulse characterization at free-electron
lasers. Nat. Photonics.

[69] Diez M., Kirchberg H., Galler A., Schulz S., Biednov M., Bömer C., Choi T.-K., Rodriguez-Fernandez A., Gawelda W., Khakhulin D. (2023). A sensitive high repetition
rate arrival time monitor for X-ray free electron lasers. Nat. Commun..

[70] Yang Z., Schnorr K., Bhattacherjee A., Lefebvre P.-L., Epshtein M., Xue T., Stanton J. F., Leone S. R. (2018). Electron-withdrawing effects in the
photodissociation of CH2ICl to form CH2Cl radical, simultaneously
viewed through the carbon K and chlorine L2, 3 X-ray edges. J. Am. Chem. Soc..

[71] Chang K. F., Reduzzi M., Wang H., Poullain S. M., Kobayashi Y., Barreau L., Prendergast D., Neumark D. M., Leone S. R. (2020). Revealing
electronic state-switching at conical intersections in alkyl iodides
by ultrafast XUV transient absorption spectroscopy. Nat. Commun..

[72] Olney T. N., Cooper G., Brion C. (1998). Quantitative
studies of the photoabsorption
(4.5–488 eV) and photoionization (9–59.5 eV) of methyl
iodide using dipole electron impact techniques. Chemical Physics.

[73] Attar A. R., Piticco L., Leone S. R. (2014). Core-to-valence
spectroscopic detection
of the CH2Br radical and element-specific femtosecond photodissociation
dynamics of CH2IBr. J. Chem. Phys..

[74] Scarborough T. D., Gorman T. T., Mauger F., Sándor P., Khatri S., Gaarde M. B., Schafer K. J., Agostini P., DiMauro L. F. (2018). Full characterization of a molecular
cooper minimum
using high-harmonic spectroscopy. Applied Sciences.

[75] Dessent C. E., Haines S. R., Müller-Dethlefs K. (1999). A new detection
scheme
for synchronous, high resolution ZEKE and MATI spectroscopy demonstrated
on the Phenol· Ar complex. Chem. Phys.
Lett..

[76] Kostko O., Kim S. K., Leone S. R., Ahmed M. (2009). Mass-analyzed threshold
ionization (MATI) spectroscopy of atoms and molecules using VUV synchrotron
radiation. J. Phys. Chem. A.

[77] Prince K. C., Richter R., de Simone M., Alagia M., Coreno M. (2003). Near edge
X-ray absorption spectra of some small polyatomic molecules. J. Phys. Chem. A.

[78] Wang H., Friedrich S., Li L., Mao Z., Ge P., Balasubramanian M., Patil D. S. (2018). L-edge sum rule analysis on 3d transition
metal sites: from d 10 to d 0 and towards application to extremely
dilute metallo-enzymes. Phys. Chem. Chem. Phys..

[79] Wheeler J., Bearden J. (1934). The variation of the
K resonating strength with atomic
number. Phys. Rev..

[80] Schäfer, M. ; Merkt, F. Frontiers of Molecular Spectroscopy; Elsevier: 2009; pp 35–61.

[81] Garratt D., Matthews M., Marangos J. (2024). Toward ultrafast soft
x-ray spectroscopy
of organic photovoltaic devices. Structural
Dynamics.

[82] Kleine C., Winghart M.-O., Zhang Z.-Y., Richter M., Ekimova M., Eckert S., Vrakking M. J., Nibbering E. T., Rouzée A., Grant E. R. (2022). Electronic state
population dynamics
upon ultrafast strong field ionization and fragmentation of molecular
nitrogen. Phys. Rev. Lett..

[83] Saito N., Sannohe H., Ishii N., Kanai T., Kosugi N., Wu Y., Chew A., Han S., Chang Z., Itatani J. (2019). Real-time
observation of electronic, vibrational, and rotational dynamics in
nitric oxide with attosecond soft x-ray pulses at 400 eV. Optica.

[84] Zhang Z.-Y., Restaino L., Sen A., Winghart M.-O., Coates M. R., Odelius M., Kowalewski M., Nibbering E. T., Rouzée A. (2024). Ultrafast Mapping of Electronic and Nuclear Structure
in the Photo Dissociation of Nitrogen Dioxide. J. Phys. Chem. Lett..

[85] Kleine C., Ekimova M., Goldsztejn G., Raabe S., Struber C., Ludwig J., Yarlagadda S., Eisebitt S., Vrakking M. J., Elsaesser T. (2019). Soft X-ray absorption spectroscopy of aqueous
solutions using a table-top femtosecond soft X-ray source. J. Phys. Chem. Lett..

[86] Smith A. D., Balciunas T., Chang Y.-P., Schmidt C., Zinchenko K., Nunes F. B., Rossi E., Svoboda V., Yin Z., Wolf J.-P. (2020). Femtosecond soft-X-ray absorption spectroscopy
of liquids
with a water-window high-harmonic source. J.
Phys. Chem. Lett..

[87] Yin Z., Chang Y.-P., Balciunas T., Shakya Y., Djorovic A., Gaulier G., Fazio G., Santra R., Inhester L., Wolf J.-P., Worner H. J. (2023). Femtosecond proton transfer
in urea solutions probed by X-ray spectroscopy. Nature.

[88] Chang Y.-P., Balciunas T., Yin Z., Sapunar M., Tenorio B. N., Paul A. C., Tsuru S., Koch H., Wolf J.-P., Coriani S. (2025). Electronic dynamics created at conical intersections
and its dephasing in aqueous solution. Nat.
Phys..

[89] Garratt D., Misiekis L., Wood D., Larsen E. W., Matthews M., Alexander O., Ye P., Jarosch S., Ferchaud C., Strüber C. (2022). Direct observation of ultrafast exciton localization
in an organic semiconductor with soft X-ray transient absorption spectroscopy. Nat. Commun..

[90] Wörner H. J., Wolf J.-P. (2025). Ultrafast spectroscopy
of liquids using extreme-ultraviolet
to soft-X-ray pulses. Nature Reviews Chemistry.

[91] Reinhard J., Wiesner F., Hennecke M., Sidiropoulos T., Kaleta S., Späthe J., Abel J. J., Wünsche M., Schmidl G., Plentz J. (2026). Soft X-ray imaging with
coherence tomography in the water window spectral range using high-harmonic
generation. Light: Science & Applications.

[92] Ramasesha K., Leone S. R., Neumark D. M. (2016). Real-time
probing of electron dynamics
using attosecond time-resolved spectroscopy. Annu. Rev. Phys. Chem..

[93] Takahashi E. J., Kanai T., Ishikawa K. L., Nabekawa Y., Midorikawa K. (2008). Coherent Water
Window X Ray by Phase-Matched High-Order Harmonic Generation in Neutral
Media. Phys. Rev. Lett..

[94] Popmintchev T., Chen M.-C., Bahabad A., Gerrity M., Sidorenko P., Cohen O., Christov I. P., Murnane M. M., Kapteyn H. C. (2009). Phase matching
of high harmonic generation in the soft and hard X-ray regions of
the spectrum. Proc. Natl. Acad. Sci. U. S. A..

[95] Chen M.-C., Arpin P., Popmintchev T., Gerrity M., Zhang B., Seaberg M., Popmintchev D., Murnane M., Kapteyn H. (2010). Bright, Coherent,
Ultrafast Soft X-Ray Harmonics Spanning the Water Window from a Tabletop
Light Source. Physical Review Letters.

[96] Popmintchev T., Chen M.-C., Popmintchev D., Arpin P., Brown S., Ališauskas S., Andriukaitis G., Balčiunas T., Mücke O. D., Pugzlys A. (2012). Bright Coherent Ultrahigh
Harmonics in the keV X-ray Regime from Mid-Infrared Femtosecond Lasers. Science.

[97] Cousin S., Silva F., Teichmann S., Hemmer M., Buades B., Biegert J. (2014). High-flux table-top soft x-ray source driven by sub-2-cycle,
CEP stable, 1.85-*μ*m 1-kHz pulses for carbon
K-edge spectroscopy. Optics letters.

[98] Ishii N., Kaneshima K., Kitano K., Kanai T., Watanabe S., Itatani J. (2014). Carrier-envelope
phase-dependent high harmonic generation
in the water window using few-cycle infrared pulses. Nat. Commun..

[99] Dorner-Kirchner M., Shumakova V., Coccia G., Kaksis E., Schmidt B. E., Pervak V., Pugzlys A., Baltuska A., Kitzler-Zeiler M., Carpeggiani P. A. (2023). HHG at the carbon K-edge directly driven by SRS red-shifted
pulses from an ytterbium amplifier. ACS Photonics.

[100] Yan J., Qin W., Chen Y., Decking W., Dijkstal P., Guetg M., Inoue I., Kujala N., Liu S., Long T. (2024). Terawatt-attosecond
hard X-ray free-electron laser
at high repetition rate. Nat. Photonics.

[101] Yang G., Shen Y. R. (1984). Spectral broadening
of ultrashort
pulses in a nonlinear medium. Opt. Lett..

[102] Brodeur A., Chin S. L. (1999). Ultrafast white-light
continuum generation
and self-focusing in transparent condensed media. Journal of the Optical Society of America B.

[103] Bradler M., Baum P., Riedle E. (2009). Femtosecond
continuum
generation in bulk laser host materials with sub-*μ*J pump pulses. Applied Physics B: Lasers and
Optics.

[104] Boyd, R. W. Nonlinear Optics, 3rd ed.; Academic Press: 2008.

[105] Schaffer C. B., Brodeur A., Mazur E. (2001). Laser-induced breakdown
and damage in bulk transparent materials induced by tightly focused
femtosecond laser pulses. Measurement Science
and Technology.

[106] Keldysh L. V. (1965). Ionization in the field of a strong electromagnetic
wave. Soviet Physics JETP.

[107] Winterfeldt C., Spielmann C., Gerber G. (2008). Colloquium: Optimal
control of high-harmonic generation. Rev. Mod.
Phys..

[108] Corkum P. B. (1993). Plasma
perspective on strong field multiphoton ionization. Phys. Rev. Lett..

[109] Ammosov, M. V. ; Delone, N. B. ; Krainov, V. P. In High Intensity Laser Processes; Alcock, J. A. , Ed.; SPIE: 1986; Vol. 0664; pp 138–141.

[110] Tong X. M., Zhao Z. X., Lin C. D. (2002). Theory
of molecular
tunneling ionization. Phys. Rev. A.

[111] Tate J., Auguste T., Muller H. G., Salières P., Agostini P., DiMauro L. F. (2007). Scaling of Wave-Packet
Dynamics in
an Intense Midinfrared Field. Phys. Rev. Lett..

[112] Shiner A. D., Trallero-Herrero C., Kajumba N., Bandulet H.-C., Comtois D., Légaré F., Giguère M., Kieffer J.-C., Corkum P. B., Villeneuve D. M. (2009). Wavelength
Scaling of High Harmonic Generation Efficiency. Phys. Rev. Lett..

[113] Heyl C. M., Arnold C. L., Couairon A., L’Huillier A. (2017). Introduction
to macroscopic power scaling principles for high-order harmonic generation. J. Phys. B: At. Mol. Opt. Phys..

[114] Brabec T., Krausz F. (2000). Intense few-cycle laser
fields: Frontiers
of nonlinear optics. Rev. Mod. Phys..

[115] Gaarde M. B., Tate J. L., Schafer K. J. (2008). Macroscopic
aspects
of attosecond pulse generation. J. Phys. B:
At. Mol. Opt. Phys..

[116] Lewenstein M., Balcou P., Ivanov M. Y., L’Huillier A., Corkum P. B. (1994). Theory of high-harmonic generation
by low-frequency
laser fields. Phys. Rev. A.

[117] Ben-Tal N., Moiseyev N., Beswick A. (1993). The effect
of Hamiltonian
symmetry on generation of odd and even harmonics. J. Phys. B: At. Mol. Opt. Phys..

[118] Sayrac M., Kolomenskii A. A., Dong J., Schuessler H. A. (2021). Generation
of even and odd harmonics in the XUV region with controlling the relative
delay and polarization of two-color fields. Optik.

[119] Rulliere, C. Femtosecond Laser Pulses: Principles and Experiments, 2nd ed.; Springer: 2003.

[120] Kondo K., Kobayashi Y., Sagisaka A., Nabekawa Y., Watanabe S. (1996). Tunneling
ionization and harmonic generation in two-color
fields. Journal of the Optical Society of America
B.

[121] Zamith S., Ni Y., Gürtler A., Noordam L., Muller H., Vrakking M. (2004). Control of atomic ionization
by two-color few-cycle pulses. Opt. Lett..

[122] Ehlotzky F. (2001). Atomic phenomena in bichromatic laser
fields. Phys. Rep..

[123] Rundquist A., Durfee C. G., Chang Z., Herne C., Backus S., Murnane M. M., Kapteyn H. C. (1998). Phase-Matched
Generation
of Coherent Soft X-rays. Science.

[124] Feng S., Winful H. G. (2001). Physical origin
of the Gouy phase
shift. Opt. Lett..

[125] Lindner F., Paulus G. G., Walther H., Baltuška A., Goulielmakis E., Lezius M., Krausz F. (2004). Gouy Phase
Shift for
Few-Cycle Laser Pulses. Phys. Rev. Lett..

[126] Li X. F., L’Huillier A., Ferray M., Lompré L. A., Mainfray G. (1989). Multiple-harmonic generation
in rare gases at high
laser intensity. Phys. Rev. A.

[127] Marcatili E. A. J., Schmeltzer R. A. (1964). Hollow
Metallic and Dielectric Waveguides
for Long Distance Optical Transmission and Lasers. Bell System Technical Journal.

[128] Durfee C. G., Rundquist A. R., Backus S., Herne C., Murnane M. M., Kapteyn H. C. (1999). Phase Matching
of High-Order Harmonics
in Hollow Waveguides. Phys. Rev. Lett..

[129] Zhu B., Fu Z., Chen Y., Peng S., Jin C., Fan G., Zhang S., Wang S., Ru H., Tian C. (2022). Spatially
homogeneous few-cycle compression of Yb lasers via all-solid-state
free-space soliton management. Opt. Express.

[130] Klas R., Eschen W., Kirsche A., Rothhardt J., Limpert J. (2020). Generation of coherent broadband
high photon flux continua
in the XUV with a sub-two-cycle fiber laser. Opt. Express.

[131] Spielmann C., Burnett N. H., Sartania S., Koppitsch R., Schnürer M., Kan C., Lenzner M., Wobrauschek P., Krausz F. (1997). Generation of Coherent X-rays in the Water Window Using
5-fs Laser Pulses. Science.

[132] Chen Y., Fu Z., Li B., Peng S., Zhu B., Fan G., Liu Y., Ding C., Jin C., Tao Z. (2023). Phase-Matched High-Harmonic
Generation under Nonadiabatic Conditions:
Model and Experiment. Ultrafast Science.

[133] Geissler M., Tempea G., Brabec T. (2000). Phase-matched
high-order
harmonic generation in the nonadiabatic limit. Phys. Rev. A.

[134] Fu Z., Chen Y., Peng S., Zhu B., Li B., Martín-Hernández R., Fan G., Wang Y., Hernández-García C., Jin C. (2022). Extension
of the bright high-harmonic photon energy range via nonadiabatic critical
phase matching. Science Advances.

[135] Ferray M, L'Huillier A, Li X F, Lompre L A, Mainfray G, Manus C (1988). Multiple-harmonic conversion
of 1064
nm radiation in rare gases. J. Phys. B: At.
Mol. Opt. Phys..

[136] Hänsch T. (1990). A proposed sub-femtosecond pulse
synthesizer using
separate phase-locked laser oscillators. Opt.
Commun..

[137] Harris S. E., Macklin J. J., Hansch T. W. (1993). Atomic scale temporal
structure inherent to high-order harmonic generation. Opt. Commun..

[138] Farkas G., Tóth C. (1992). Proposal for attosecond light pulse
generation using laser induced multiple-harmonic conversion processes
in rare gases. Phys. Lett. A.

[139] L’Huillier A., Schafer K. J., Kulander K. C. (1991). Theoretical
aspects
of intense field harmonic generation. J. Phys.
B: At. Mol. Opt. Phys..

[140] Lompré L., L’Huillier A., Ferray M., Monot P., Mainfray G., Manus C. (1990). High-order
harmonic generation in
xenon: intensity and propagation effects. Journal
of the Optical Society of America B.

[141] Hentschel M., Kienberger R., Spielmann C., Reider G. A., Milosevic N., Brabec T., Corkum P., Heinzmann U., Drescher M., Krausz F. (2001). Attosecond metrology. Nature.

[142] Pfeifer T., Abel M. J., Nagel P. M., Boutu W., Bell M. J., Liu Y., Neumark D. M., Leone S. R. (2009). Measurement
and optimization of isolated attosecond pulse contrast. Opt. Lett..

[143] Frank F., Arrell C., Witting T., Okell W. A., McKenna J., Robinson J. S., Haworth C. A., Austin D., Teng H., Walmsley I. A. (2012). Invited Review Article:
Technology for Attosecond Science. Rev. Sci.
Instrum..

[144] Wang H., Chini M., Khan S. D., Chen S., Gilbertson S., Feng X., Mashiko H., Chang Z. (2009). Practical
issues of retrieving isolated attosecond pulses. J. Phys. B: At. Mol. Opt. Phys..

[145] Chini M., Mashiko H., Wang H., Chen S., Yun C., Scott S., Gilbertson S., Chang Z. (2009). Delay control in attosecond
pump-probe experiments. Opt. Express.

[146] Goulielmakis E., Uiberacker M., Kienberger R., Baltuska A., Yakovlev V., Scrinzi A., Westerwalbesloh T., Kleineberg U., Heinzmann U., Drescher M. (2004). Direct
Measurement of Light Waves. Science.

[147] Goulielmakis E., Schultze M., Hofstetter M., Yakovlev V. S., Gagnon J., Uiberacker M., Aquila A. L., Gullikson E. M., Attwood D. T., Kienberger R. (2008). Single-Cycle Nonlinear Optics. Science.

[148] Weissenbilder R., Carlström S., Rego L., Guo C., Heyl C. M., Smorenburg P., Constant E., Arnold C. L., L’Huillier A. (2022). How to optimize
high-order harmonic generation in gases. Nature
Reviews Physics.

[149] Tempea G., Brabec T. (2000). Optimization of high-harmonic generation. Appl. Phys. B: Laser Opt..

[150] Loh Z.-H., Khalil M., Correa R. E., Leone S. R. (2008). A tabletop
femtosecond time-resolved soft x-ray transient absorption spectrometer. Rev. Sci. Instrum..

[151] Constant E., Garzella D., Breger P., Mével E., Dorrer C., Blanc C. L., Salin F., Agostini P. (1999). Optimizing
High Harmonic Generation in Absorbing Gases: Model and Experiment. Phys. Rev. Lett..

[152] Bartels R. A., Paul A., Green H., Kapteyn H. C., Murnane M. M., Backus S., Christov I. P., Liu Y., Attwood D., Jacobsen C. (2002). Generation of Spatially Coherent
Light at Extreme Ultraviolet Wavelengths. Science.

[153] Schnorr K., Bhattacherjee A., Oosterbaan K. J., Delcey M. G., Yang Z., Xue T., Attar A. R., Chatterley A. S., Head-Gordon M., Leone S. R. (2019). Tracing
the 267 nm-induced radical formation in dimethyl disulfide using time-resolved
x-ray absorption spectroscopy. J. Phys. Chem.
Lett..

[154] Quinlan F., Fortier T. M., Jiang H., Hati A., Nelson C., Fu Y., Campbell J. C., Diddams S. A. (2013). Exploiting
shot noise correlations in the photodetection of ultrashort optical
pulse trains. Nat. Photonics.

[155] Zia H. (2018). Simulation of white light generation
and near light bullets using
a novel numerical technique. Communications
in Nonlinear Science and Numerical Simulation.

[156] Chen J., Hengyuan X., Zhang T., Siqin D., Hua J., Wei L. (2025). Energy stability of
supercontinuum via femtosecond
filamentation in sapphire. Opt. Express.

[157] Calendron A.-L., Çankaya H., Cirmi G., Kärtner F. X. (2015). White-light
generation with sub-ps pulses. Opt. Express.

[158] Altucci C., Bruzzese R., Lisio C. D., Nisoli M., Stagira S., Silvestri S. D., Svelto O., Boscolo A., Ceccherini P., Poletto L. (1999). Tunable soft-x-ray radiation
by high-order harmonic generation. Phys. Rev.
A.

[159] Shin H. J., Lee D. G., Cha Y. H., Hong K. H., Nam C. H. (1999). Generation of Nonadiabatic Blueshift
of High Harmonics
in an Intense Femtosecond Laser Field. Phys.
Rev. Lett..

[160] Steingrube D. S., Vockerodt T., Schulz E., Morgner U., Kovačev M. (2009). Phase matching
of high-order harmonics in a semi-infinite
gas cell. Phys. Rev. A.

[161] Volkov M., Pupeikis J., Phillips C. R., Schlaepfer F., Gallmann L., Keller U. (2019). Reduction of laser-intensity-correlated
noise in high-harmonic generation. Opt. Express.

[162] Künzel S., Williams G. O., Boutu W., Galtier E., Barbrel B., Lee H. J., Nagler B., Zastrau U., Dovillaire G., Lee R. W. (2015). Shot-to-shot
intensity
and wavefront stability of high-harmonic generation. Appl. Opt..

[163] Géneaux R., Chang H.-T., Schwartzberg A. M., Marroux H. J. (2021). Source noise suppression in attosecond transient absorption
spectroscopy by edge-pixel referencing. Opt.
Express.

[164] Faccialà D., Toulson B. W., Gessner O. (2021). Removal of correlated
background in a high-order harmonic transient absorption spectra with
principal component regression. Opt. Express.

[165] Ash R., Abhari Z., Candela R., Welke N., Murawski J., Gardezi S. M., Venkatasubramanian N., Munawar M., Siewert F., Sokolov A. (2023). X-FAST:
A versatile, high-throughput, and user-friendly
XUV femtosecond absorption spectroscopy tabletop instrument. Rev. Sci. Instrum..

[166] Grant-Jacob J., Mills B., Butcher T. J., Chapman R. T., Brocklesby W. S., Frey J. G. (2011). Gas jet structure influence on high
harmonic generation. Opt. Express.

[167] Sutherland J. R., Christensen E. L., Powers N. D., Rhynard S. E., Painter J. C., Peatross J. (2004). High harmonic
generation in a semi-infinite
gas cell. Opt. Express.

[168] Vismarra F., Fernández-Galán M., Mocci D., Colaizzi L., Segundo V. W., Boyero-
García R., Serrano J., Conejero-Jarque E., Pini M., Mai L. (2024). Isolated attosecond
pulse generation in a semi-infinite gas cell driven by time-gated
phase matching. Light: Science & Applications.

[169] Moore, J. H. ; Davis, C. C. ; Coplan, M. A. Building scientific apparatus: a practical guide to design and construction; Addison-Wesley Publishing Company: 1983.

[170] Stebbings S. L., Rogers E. T., Paula A. M. D., Praeger M., Froud C. A., Mills B., Hanna D. C., Baumberg J. J., Brocklesby W. S., Frey J. G. (2008). Molecular variation
of capillary-produced
soft x-ray high harmonics. J. Phys. B: At. Mol.
Opt. Phys..

[171] Shari’ati Y., Vura-Weis J. (2021). Polymer thin films as universal substrates
for extreme ultraviolet absorption spectroscopy of molecular transition
metal complexes. Journal of Synchrotron Radiation.

[172] Schreck S., Gavrila G., Weniger C., Wernet P. (2011). A sample holder
for soft x-ray absorption spectroscopy of liquids in transmission
mode. Rev. Sci. Instrum..

[173] Baker L. B. (2014). Charge carrier dynamics
of photoexcited Co3O4
in methanol: Extending high harmonic transient absorption spectroscopy
to liquid environments. Nano Lett..

[174] Cirri A., Husek J., Biswas S., Baker L. R. (2017). Achieving
Surface Sensitivity in Ultrafast XUV Spectroscopy: M2,3-Edge Reflection-Absorption
of Transition Metal Oxides. J. Phys. Chem. C.

[175] Biswas S., Baker L. R. (2022). Extreme Ultraviolet
Reflection—Absorption
Spectroscopy: Probing Dynamics at Surfaces from a Molecular Perspective. Acc. Chem. Res..

[176] Biswas S., Husek J., Baker L. R. (2018). Elucidating ultrafast
electron dynamics at surfaces using extreme ultraviolet (XUV) reflection-absorption
spectroscopy. Chem. Commun..

[177] Husek J., Cirri A., Biswas S., Baker L. R. (2017). Surface
electron dynamics in hematite (*α*-Fe2O3): Correlation
between ultrafast surface electron trapping and small polaron formation. Chemical Science.

[178] Bandaranayake S., Hruska E., Londo S., Biswas S., Baker L. R. (2020). Small Polarons
and Surface Defects in Metal Oxide Photocatalysts
Studied Using XUV Reflection-Absorption Spectroscopy. J. Phys. Chem. C.

[179] Biswas S., Wallentine S., Bandaranayake S., Baker L. R. (2019). Controlling polaron formation at hematite surfaces
by molecular functionalization probed by XUV reflection-absorption
spectroscopy. J. Chem. Phys..

[180] Biswas S., Husek J., Londo S., Baker L. R. (2018). Highly
Localized Charge Transfer Excitons in Metal Oxide Semiconductors. Nano Lett..

[181] Larsson J., Chang Z., Judd E., Schuck P. J., Falcone R. W., Heimann P. A., Padmore H. A., Kapteyn H. C., Bucksbaum P. H., Murnane M. M. (1997). Ultrafast x-ray diffraction
using a streak-camera detector in averaging mode. Opt. Lett..

[182] Nyakuchena J., Zhang X., Huang J. (2023). Synchrotron based transient
x-ray absorption spectroscopy for emerging solid-state energy materials. Chemical Physics Reviews.

[183] Paresce F. (1975). Quantum efficiency
of a channel electron multiplier
in the far ultraviolet. Appl. Opt..

[184] Holland S. E., Groom D. E., Palaio N. P., Stover R. J., Wei M. (2003). Fully depleted, back-illuminated
charge-coupled devices fabricated
on high-resistivity silicon. IEEE Trans. Electron
Devices.

[185] Poletto L., Boscolo A., Tondello G. (1999). Characterization of
a charge-coupled-device detector in the 1100–0.14-nm 1-eV to
9-keV spectral region. Appl. Opt..

[186] Moy J.-P. (2000). Recent developments in X-ray imaging
detectors. Nuclear Instruments and Methods in
Physics Research A.

[187] Attar A. R., Bhattacherjee A., Pemmaraju C., Schnorr K., Closser K. D., Prendergast D., Leone S. R. (2017). Femtosecond x-ray spectroscopy of an electrocyclic
ring-opening reaction. Science.

[188] Nakano N., Kuroda H., Kita T., Harada T. (1984). Development
of a flat-field grazing-incidence XUV spectrometer and its application
in picosecond XUV spectroscopy. Appl. Opt..

[189] Wang X., Chini M., Cheng Y., Wu Y., Chang Z. (2013). In situ calibration of an extreme ultraviolet spectrometer
for attosecond
transient absorption experiments. Appl. Opt..

[190] Brodeur A., Chin S. L. (1998). Band-Gap Dependence
of the Ultrafast
White-Light Continuum. Phys. Rev. Lett..

[191] Corkum P. B., Rolland C., Srinivasan-Rao T. (1986). Supercontinuum
Generation in Gases. Phys. Rev. Lett..

[192] Brimhall N., Painter J. C., Powers N., Giraud G., Turner M., Ware M., Peatross J. (2007). Measured laser-beam
evolution during high-order harmonic generation in a semi-infinite
gas cell. Opt. Express.

[193] Takahashi E. J., Nabekawa Y., Midorikawa K. (2004). Low-divergence
coherent soft x-ray source at 13 nm by high-order harmonics. Appl. Phys. Lett..

[194] Roscam
Abbing S., Campi F., Sajjadian F. S., Lin N., Smorenburg P., Kraus P. M. (2020). Divergence control of high-harmonic
generation. Physical Review Applied.

[195] Hilbert V., Tschernajew M., Klas R., Limpert J., Rothhardt J. (2020). A compact,
turnkey, narrow-bandwidth, tunable, and
high-photon-flux extreme ultraviolet source. AIP Advances.

[196] Wang H., Xu Y., Ulonska S., Robinson J. S., Ranitovic P., Kaindl R. A. (2015). Bright high-repetition-rate
source
of narrowband extreme-ultraviolet harmonics beyond 22 eV. Nat. Commun..

[197] Chen M.-C., Gerrity M., Backus S., Popmintchev T., Zhou X., Arpin P., Zhang X., Kapteyn H., Murnane M. (2009). Spatially coherent, phase matched,
high-order harmonic
EUV beams at 50 kHz. Opt. Express.

[198] Miao J., Ishikawa T., Robinson I. K., Murnane M. M. (2015). Beyond
crystallography: Diffractive imaging using coherent x-ray light sources. Science.

[199] Lin M.-F., Verkamp M. A., Ryland E. S., Zhang K., Vura-Weis J. (2016). Impact of spatial chirp on high-harmonic extreme ultraviolet
absorption spectroscopy of thin films. Journal
of the Optical Society of America B.

[200] Jager M. F., Ott C., Kaplan C. J., Kraus P. M., Neumark D. M., Leone S. R. (2018). Attosecond transient
absorption instrumentation
for thin film materials: Phase transitions, heat dissipation, signal
stabilization, timing correction, and rapid sample rotation. Rev. Sci. Instrum..

[201] Zhang B., Gardner D. F., Seaberg M. D., Shanblatt E. R., Kapteyn H. C., Murnane M. M., Adams D. E. (2015). High contrast 3D
imaging of surfaces near the wavelength limit using tabletop EUV ptychography. Ultramicroscopy.

[202] Shanblatt E. R., Porter C. L., Gardner D. F., Mancini G. F., Karl R. M., Tanksalvala M. D., Bevis C. S., Vartanian V. H., Kapteyn H. C., Adams D. E. (2016). Quantitative Chemically
Specific Coherent Diffractive Imaging of Reactions at Buried Interfaces
with Few Nanometer Precision. Nano Lett..

[203] Gardner D. F., Tanksalvala M., Shanblatt E. R., Zhang X., Galloway B. R., Porter C. L., Karl R., Bevis C., Adams D. E., Kapteyn H. C. (2017). Subwavelength
coherent imaging of periodic samples using a 13.5 nm tabletop high-harmonic
light source. Nat. Photonics.

[204] Fienup J. R. (1978). Reconstruction of an object from
the modulus of its
Fourier transform. Opt. Lett..

[205] Marchesini S., He H., Chapman N., Hau-Riege P., Noy A., Howells R., Weierstall U., Spence H. (2003). X-ray image reconstruction
from a diffraction pattern alone. Physical Review
B - Condensed Matter and Materials Physics.

[206] Fienup J. R. (1982). Phase retrieval algorithms: a comparison. Appl. Opt..

[207] Elser V. (2003). Random projections and the optimization of an algorithm for phase
retrieval. Journal of Physics A: Mathematical
and General.

[208] Maiden A. M., Rodenburg J. M. (2009). An improved ptychographical phase
retrieval algorithm for diffractive imaging. Ultramicroscopy.

[209] Seaberg M. D., Zhang B., Gardner D. F., Shanblatt E. R., Murnane M. M., Kapteyn H. C., Adams D. E. (2014). Tabletop
nanometer
extreme ultraviolet imaging in an extended reflection mode using coherent
Fresnel ptychography. Optica.

[210] Ishii N., Itakura R. (2024). Sub-two-cycle intense
pulse generation
based on two-stage hollow-core fiber compression using an ytterbium
amplifier. Applied Physics Express.

[211] Beetar J. E., Rivas F., Gholam-Mirzaei S., Liu Y., Chini M. (2019). Hollow-core fiber compression of a commercial Yb:KGW
laser amplifier. J. Opt. Soc. Am. B.

[212] Schmidt C., Pertot Y., Balciunas T., Zinchenko K., Matthews M., Wörner H. J., Wolf J.-P. (2018). High-order harmonic source spanning up to the oxygen
K-edge based on filamentation pulse compression. Opt. Express.

[213] Zhao K., Zhang Q., Chini M., Wu Y., Wang X., Chang Z. (2012). Tailoring a 67 attosecond pulse through
advantageous phase-mismatch. Opt. Lett..

[214] Midorikawa K. (2022). Progress on table-top isolated attosecond
light sources. Nat. Photonics.

[215] Sansone G., Poletto L., Nisoli M. (2011). High-energy
attosecond
light sources. Nat. Photonics.

[216] Feng X., Gilbertson S., Mashiko H., Wang H., Khan S. D., Chini M., Wu Y., Zhao K., Chang Z. (2009). Generation of isolated attosecond
pulses with 20 to 28 fs lasers. Phys. Rev. Lett..

[217] Abel M. J., Pfeifer T., Nagel P. M., Boutu W., Bell M. J., Steiner C. P., Neumark D. M., Leone S. R. (2009). Isolated
attosecond pulses from ionization gating of high-harmonic emission. Chem. Phys..

[218] Beckwith J. S., Rumble C. A., Vauthey E. (2020). Data analysis
in transient
electronic spectroscopy–an experimentalist’s view. Int. Rev. Phys. Chem..

[219] Heinrich T., Chang H.-T., Zayko S., Rossnagel K., Sivis M., Ropers C. (2023). Electronic and structural fingerprints
of charge-density-wave excitations in extreme ultraviolet transient
absorption spectroscopy. Physical Review X.

[220] Zhang K., Lin M.-F., Ryland E. S., Verkamp M. A., Benke K., De Groot F. M. F., Girolami G. S., Vura-Weis J. (2016). Shrinking
the Synchrotron: Tabletop Extreme Ultraviolet Absorption of Transition-Metal
Complexes. J. Phys. Chem. Lett..

[221] Troß J., Arias-Martinez J. E., Carter-Fenk K., Cole-Filipiak N. C., Schrader P., McCaslin L. M., Head-Gordon M., Ramasesha K. (2024). Femtosecond core-level spectroscopy
reveals involvement
of triplet states in the gas-phase photodissociation of Fe­(CO)­5. J. Am. Chem. Soc..

[222] Feng Y., Vinogradov I., Ge N.-H. (2017). General noise suppression
scheme with reference detection in heterodyne nonlinear spectroscopy. Opt. Express.

[223] Robben K. C., Cheatum C. M. (2020). Edge-pixel referencing suppresses
correlated baseline noise in heterodyned spectroscopies. J. Chem. Phys..

[224] Zinchenko K. S., Ardana-Lamas F., Lanfaloni V. U., Luu T. T., Pertot Y., Huppert M., Wörner H. J. (2023). Apparatus
for attosecond transient-absorption spectroscopy in the water-window
soft-X-ray region. Sci. Rep..

[225] Ott C., Kaldun A., Raith P., Meyer K., Laux M., Evers J., Keitel C. H., Greene C. H., Pfeifer T. (2013). Lorentz meets
Fano in spectral line shapes: a universal phase and its laser control. Science.

[226] Wenzel J., Wormit M., Dreuw A. (2014). Calculating core-level
excitations and X-ray absorption spectra of medium-sized closed-shell
molecules with the algebraic-diagrammatic construction scheme for
the polarization propagator. Journal of Computational
Chemistry.

[227] Seidu I., Neville S. P., Kleinschmidt M., Heil A., Marian C. M., Schuurman M. S. (2019). The simulation
of X-ray absorption spectra from ground and excited electronic states
using core-valence separated DFT/MRCI. J. Chem.
Phys..

[228] Bhattacherjee A., Schnorr K., Oesterling S., Yang Z., Xue T., de Vivie-Riedle R., Leone S. R. (2018). Photoinduced heterocyclic ring opening of furfural:
Distinct open-chain product identification by ultrafast X-ray transient
absorption spectroscopy. J. Am. Chem. Soc..

[229] Ashfold M. N., Bain M., Hansen C. S., Ingle R. A., Karsili T. N., Marchetti B., Murdock D. (2017). Exploring the dynamics
of the photoinduced ring-opening of heterocyclic molecules. J. Phys. Chem. Lett..

[230] Faber R., Kjønstad E. F., Koch H., Coriani S. (2019). Spin adapted
implementation of EOM-CCSD for triplet excited states: Probing intersystem
crossings of acetylacetone at the carbon and oxygen K-edges. J. Chem. Phys..

[231] Chen X.-B., Fang W.-H., Phillips D. L. (2006). Theoretical
studies
of the photochemical dynamics of acetylacetone: Isomerzation, dissociation,
and dehydration reactions. J. Phys. Chem. A.

[232] Miller N. A., Deb A., Alonso-Mori R., Garabato B. D., Glownia J. M., Kiefer L. M., Koralek J., Sikorski M., Spears K. G., Wiley T. E. (2017). Polarized
XANES monitors femtosecond structural evolution of photoexcited vitamin
B12. J. Am. Chem. Soc..

[233] Paulus B. C., McCusker J. K. (2022). On the use of vibronic
coherence
to identify reaction coordinates for ultrafast excited-state dynamics
of transition metal-based chromophores. Faraday
Discuss..

[234] Chergui M. (2016). Time-resolved
X-ray spectroscopies of chemical systems:
New perspectives. Structural Dynamics.

[235] Clark-Baldwin K., Tierney D. L., Govindaswamy N., Gruff E. S., Kim C., Berg J., Koch S. A., Penner-Hahn J. E. (1998). The limitations of X-ray absorption spectroscopy for
determining the structure of zinc sites in proteins. When is a tetrathiolate
not a tetrathiolate?. J. Am. Chem. Soc..

[236] Wang M., Feng Z. (2021). Pitfalls in X-ray absorption
spectroscopy
analysis and interpretation: A practical guide for general users. Current opinion in electrochemistry.

[237] Pemberton C. C., Zhang Y., Saita K., Kirrander A., Weber P. M. (2015). From the (1B) spectroscopic state to the photochemical
product of the ultrafast ring-opening of 1, 3-cyclohexadiene: a spectral
observation of the complete reaction path. J.
Phys. Chem. A.

[238] Poisson L., Roubin P., Coussan S., Soep B., Mestdagh J.-M. (2008). Ultrafast dynamics of acetylacetone (2, 4-pentanedione)
in the S2 state. J. Am. Chem. Soc..

[239] Karashima S., Humeniuk A., Uenishi R., Horio T., Kanno M., Ohta T., Nishitani J., Mitric R., Suzuki T. (2021). Ultrafast ring-opening reaction of
1, 3-cyclohexadiene: identification of nonadiabatic pathway via doubly
excited state. J. Am. Chem. Soc..

[240] Severino S., Ziems K., Reduzzi M., Summers A., Sun H.-W., Chien Y.-H., Gräfe S., Biegert J. (2024). Attosecond core-level absorption spectroscopy reveals
the electronic and nuclear dynamics of molecular ring opening. Nat. Photonics.

[241] Wolf T. J. A., Sanchez D. M., Yang J., Parrish R. M., Nunes J. P. F., Centurion M., Coffee R., Cryan J. P., Gühr M., Hegazy K. (2019). The photochemical ring-opening
of 1, 3-cyclohexadiene imaged by ultrafast electron diffraction. Nat. Chem..

[242] Yang J., Zhu X., Nunes J. P. F., Yu J. K., Parrish R. M., Wolf T. J. A., Centurion M., Gühr M., Li R., Liu Y. (2020). Simultaneous
observation of nuclear and electronic dynamics by ultrafast electron
diffraction. Science.

[243] Squibb R. J., Sapunar M., Ponzi A., Richter R., Kivimäki A., Plekan O., Finetti P., Sisourat N., Zhaunerchyk V., Marchenko T. (2018). Acetylacetone photodynamics
at a seeded free-electron laser. Nat. Commun..

[244] Adachi S., Sato M., Suzuki T. (2015). Direct observation
of ground-state product formation in a 1, 3-cyclohexadiene ring-opening
reaction. J. Phys. Chem. Lett..

[245] Minitti M. P., Budarz J. M., Kirrander A., Robinson J. S., Ratner D., Lane T. J., Zhu D., Glownia J. M., Kozina M., Lemke H. T. (2015). Imaging
molecular motion: femtosecond x-ray scattering of an electrocyclic
chemical reaction. Phys. Rev. Lett..

[246] Spesyvtsev R., Horio T., Suzuki Y.-I., Suzuki T. (2015). Excited-state
dynamics of furan studied by sub-20-fs time-resolved photoelectron
imaging using 159-nm pulses. J. Chem. Phys..

[247] Ingle R. A., Hansen C. S., Elsdon E., Bain M., King S. J., Lee J. W., Brouard M., Vallance C., Turchetta R., Ashfold M. N. (2017). Ultraviolet photochemistry
of 2-bromothiophene
explored using universal ionization detection and multi-mass velocity-map
imaging with a PImMS2 sensor. J. Chem. Phys..

[248] Ashirov T., Siena J. S., Zhang M., Ozgur Yazaydin A., Antonietti M., Coskun A. (2022). Fast light-switchable
polymeric carbon
nitride membranes for tunable gas separation. Nat. Commun..

[249] Ortega R., Carmona A., Llorens I., Solari P. L. (2012). X-ray absorption
spectroscopy of biological samples. A tutorial. Journal of Analytical Atomic Spectrometry.

[250] Guilherme Buzanich A. (2022). Recent developments of X-ray absorption
spectroscopy
as analytical tool for biological and biomedical applications. X-Ray Spectrometry.

[251] Cutsail G. E., DeBeer S. (2022). Challenges and opportunities
for applications of advanced X-ray spectroscopy in catalysis research. ACS Catal..

[252] Öström H., Öberg H., Xin H., LaRue J. b., Beye M., Dell’Angela M., Gladh J., Ng M., Sellberg J. A., Kaya S. (2015). Probing the transition
state region in catalytic CO oxidation on Ru. Science.

[253] Nilsson A. (2025). Time-resolved
x-ray absorption spectroscopy probe in
ultrafast surface chemistry. Structural Dynamics.

[254] Jay R. M., Banerjee A., Leitner T., Wang R.-P., Harich J., Stefanuik R., Wikmark H., Coates M. R., Beale E. V., Kabanova V. (2023). Tracking C–H
activation with orbital resolution. Science.

[255] Guo W., Yin J., Xu Z., Li W., Peng Z., Weststrate C. J., Yu X., He Y., Cao Z., Wen X. (2022). Visualization of on-surface ethylene polymerization
through ethylene insertion. Science.

[256] Fomichev, S. ; Hejazi, K. ; Loaiza, I. ; Zini, M. S. ; Delgado, A. ; Voigt, A.-C. ; Mueller, J. E. ; Arrazola, J. M. Simulating X-ray absorption spectroscopy of battery materials on a quantum computer. arXiv 2024, arXiv:2405.11015 [quant-ph].

[257] Fomichev, S. ; Casares, P. A. ; Soni, J. ; Azad, U. ; Kunitsa, A. ; Voigt, A.-C. ; Mueller, J. E. ; Arrazola, J. M. Fast simulations of X-ray absorption spectroscopy for battery materials on a quantum computer. arXiv 2025, arXiv:2506.15784 [quant-ph].

[258] Minasian S. G., Keith J. M., Batista E. R., Boland K. S., Kozimor S. A., Martin R. L., Shuh D. K., Tyliszczak T., Vernon L. J. (2013). Carbon K-Edge X-ray Absorption Spectroscopy and Time-Dependent
Density Functional Theory Examination of Metal–Carbon Bonding
in Metallocene Dichlorides. J. Am. Chem. Soc..

[259] Kozimor S. A., Yang P., Batista E. R., Boland K. S., Burns C. J., Christensen C. N., Clark D. L., Conradson S. D., Hay P. J., Lezama J. S. (2008). Covalency trends in
group IV metallocene dichlorides. Chlorine K-edge X-ray absorption
spectroscopy and time dependent-density functional theory. Inorganic Chemistry.

[260] Li X., Jia X., Paz A. S., Cao Y., Glover W. J. (2022). Evidence
for water antibonding orbital mixing in the hydrated electron from
its oxygen 1s X-ray absorption spectrum. J.
Am. Chem. Soc..

[261] DiVincenzo D. P. (2000). The physical implementation of quantum computation. Fortschritte der Physik: Progress of Physics.

[262] Mukamel S., Freyberger M., Schleich W., Bellini M., Zavatta A., Leuchs G., Silberhorn C., Boyd R. W., Sánchez-Soto L.
L., Stefanov A. (2020). Roadmap on quantum light spectroscopy. J. Phys.
B: At. Mol. Opt. Phys..

[263] Rader O., Pascarelli S., Attenkofer K., Makarova A. A., Holldack K., Rossnagel K., Temst K., Kourousias G., Carretta S., Biscari C. (2026). Synchrotron Radiation
for Quantum Technology. Adv. Funct. Mater..

[264] Buades B., Picón A., Berger E., León I., Di Palo N., Cousin S. L., Cocchi C., Pellegrin E., Martin J. H., Mañas-Valero S. (2021). Attosecond state-resolved
carrier motion in quantum materials probed by soft x-ray XANES. Applied Physics Reviews.

[265] Cushing S.
K., Molesky I. J., de Roulet B. R., Lee A., Marsh B. M., Szoke S., Vaida M. E., Leone S. R. (2020). Layer-resolved
ultrafast extreme ultraviolet measurement of hole transport in a Ni-TiO2-Si
photoanode. Science Advances.

[266] Hruska E., Husek J., Bandaranayake S., Baker L. R. (2022). Visible Light Absorption and Hot Carrier Trapping in
Anatase TiO_2_: The Role of Surface Oxygen Vacancies. J. Phys. Chem. C.

[267] Liu H., Klein I. M., Michelsen J. M., Cushing S. K. (2021). Element-specific
electronic and structural dynamics using transient XUV and soft X-ray
spectroscopy. Chem..

[268] Datar A., Wright C., Matthews D. A. (2023). Theoretical
investigation
of the x-ray stark effect in small molecules. J. Phys. Chem. A.

[269] Spreinat A., Dohmen M. M., Luttgens J., Herrmann N., Klepzig L. F., Nißler R., Weber S., Mann F. A., Lauth J., Kruss S. (2021). Quantum defects in fluorescent carbon
nanotubes for sensing and mechanistic studies. J. Phys. Chem. C.

[270] Zhang Y., Huang Z., Dong C.-L., Shi J., Cheng C., Guan X., Zong S., Luo B., Cheng Z., Wei D. (2022). Synergistic effect of
nitrogen vacancy on ultrathin graphitic carbon nitride porous nanosheets
for highly efficient photocatalytic H2 evolution. Chemical Engineering Journal.

[271] Gorlach A., Tzur M. E., Birk M., Krüger M., Rivera N., Cohen O., Kaminer I. (2023). High-harmonic generation
driven by quantum light. Nat. Phys..

[272] Fan T., Grychtol P., Knut R., Hernández-García C., Hickstein D. D., Zusin D., Gentry C., Dollar F. J., Mancuso C. A., Hogle C. W. (2015). Bright circularly polarized
soft X-ray high harmonics for X-ray magnetic circular dichroism. Proc. Natl. Acad. Sci. U. S. A..

