# Supplementary material for: Ultrafast Spectroscopy with an Edge—Basic Concepts and Tabletop Applications of Femtosecond X‑ray Transient Absorption Spectroscopy: A Tutorial Review
Source: ACS Phys Chem Au. 2026 Jun 24;6(4):597–632. doi: 10.1021/acsphyschemau.6c00024 (PMC13397460; doi:10.1021/acsphyschemau.6c00024)
Supplement: Supplementary file 1 [file pg6c00024_si_001.pdf]

Ultrafast Spectroscopy with an *Edge* – Basic Concepts and Tabletop Applications of Femtosecond X-ray Transient Absorption Spectroscopy: A Tutorial Review

Caleb H. DeWitt and Aditi Bhattacherjee*

Department of Chemistry, University of Iowa, Iowa City, Iowa 52242, United States

E-mail: aditi-bhattacherjee@uiowa.edu

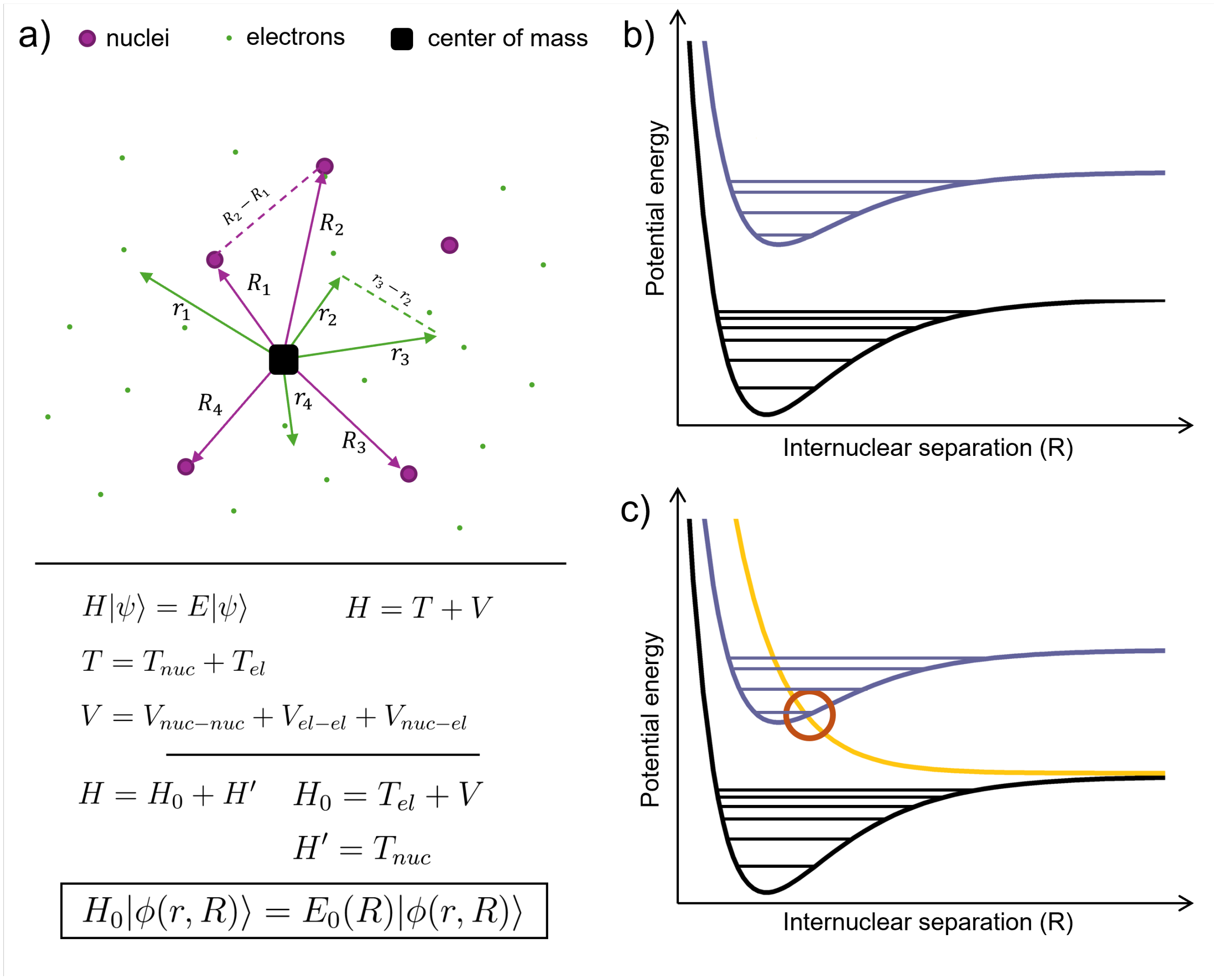

Figure S1: Premise and failure of the Born-Oppenheimer approximation. (a) For a molecule with K nuclei (mass M_k, charge Z_Ke) and N electrons (mass m, charge -e) in the center of mass frame, $H = T + V = (T_{nuc} + T_{el}) + (V_{nuc-nuc} + V_{el-el} + V_{nuc-el})$, where T and V are the kinetic energy and potential energy operators, respectively. More explicitly, $H\Psi(r,R) = \Big(-\frac{\hbar^2}{2}\sum_{k=1}^{K}\frac{1}{M_k}\nabla_k^2 - \frac{\hbar^2}{2m}\sum_{i=1}^{N}\nabla_i^2 + \frac{1}{4\pi\epsilon_0}\sum_{k=1,k>k'}^{K}\frac{Z_k e Z_{k'} e}{R_{kk'}} + \frac{1}{4\pi\epsilon_0}\sum_{i=1,i>i'}^{N}\frac{e^2}{r_{ii'}} - \frac{1}{4\pi\epsilon_0}\sum_{k=1,k>k'}^{K}\frac{Z_k e^2}{r_{ik}}\Big)\Psi = E\Psi(r,R)$ where $r_{ii'} = |r_{i'} - r_i|$, $R_{kk'} = |R_{k'} - R_k|$, and $r_{ik} = |R_k - r_i|$ denote electronic coordinates, nuclear coordinates, and electron-nuclei interparticle distances in the center of mass frame. We use the Hamiltonian $H = H_0 + H'$, where $H_0 = T_{el} + V_{nuc-nuc} + V_{el-el} + V_{nuc-el}$. (b) According to the Born-Oppenheimer approximation, $H' = T_{nuc}$ is treated as a perturbation such that solutions for the electronic energies provide the potential in which the nuclei move. Electronic potential energy curves therefore contain many vibrational levels as shown, and each vibrational level further has a set of rotational levels (not shown). (c) The Born-Oppenheimer approximation fails when potential energy surfaces approach or cross each other and non-adiabatic couplings become significant.

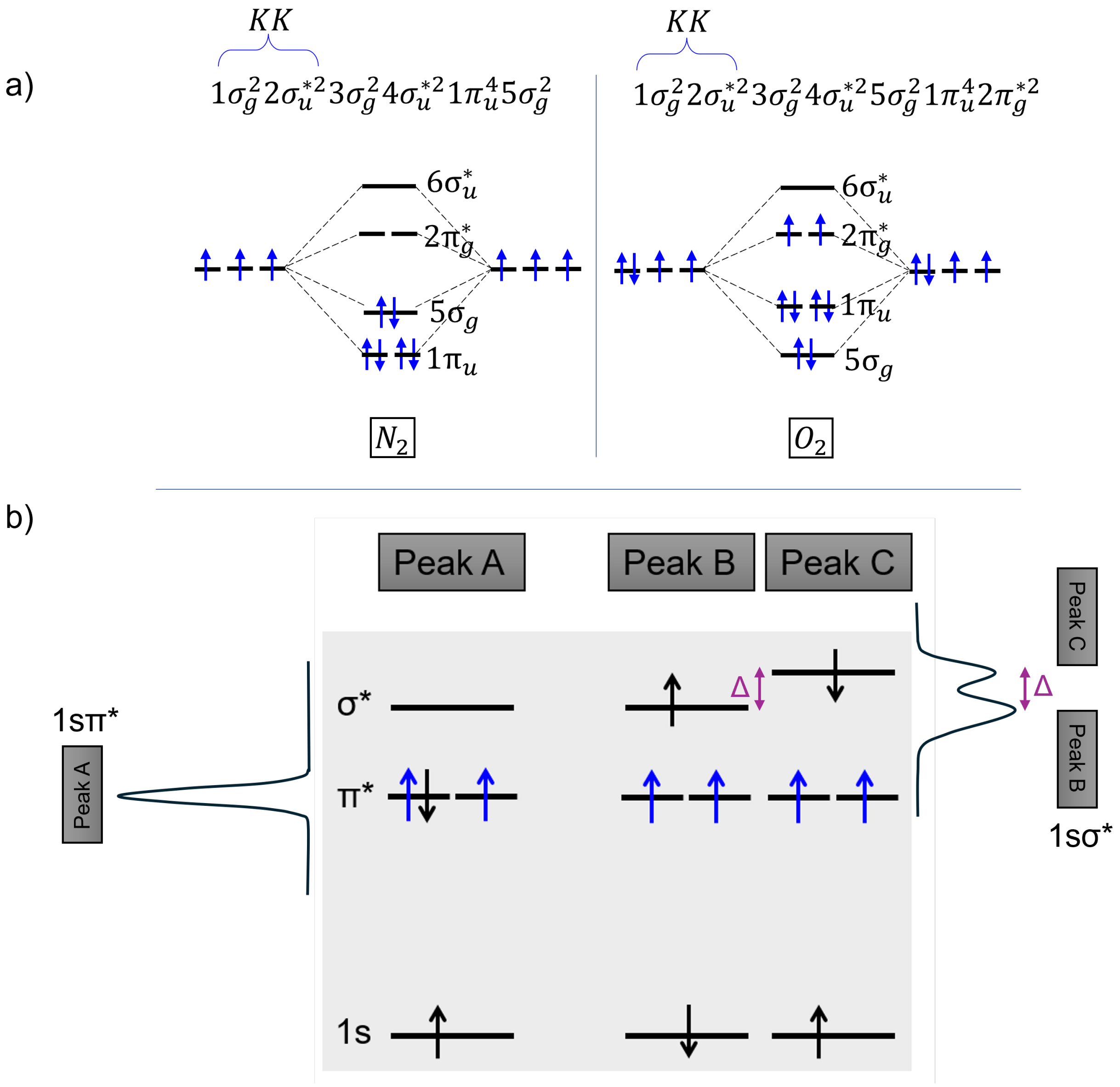

Figure S2: (a) Qualitative Hückel molecular orbital diagram for homonuclear diatomic molecules nitrogen (closed-shell) and oxygen (open-shell). (b) Cartoon depiction of exchange splitting and K-shell spectroscopy of molecular oxygen (O_2). The exchange splitting (Δ) is only observed for the σ^* resonance. Assuming the valence electrons ($2\pi_g^{*2}$) are spin-up, only a spin-down 1s core-electron can be excited into the partially occupiedπ^* level giving rise to a single $1s\pi^*$ (peak A) in the spectrum whereas two possibilities (peaks B and C) exist for excitation into the unoccupied σ^* level. Spin pairing in the final state lowers the energy by the exchange interaction energy (Δ) in comparison to the case where the spins are unpaired. The experimental value of Δ in O_2 is 3 eV.[1]

Table S1: The **molecular Schrödinger equation**, see Figure S1, is the quantum-mechanical machinery for molecules which takes into account the interaction between multiple charged particles (electron and nuclei) in a pairwise manner. It is not exactly solvable beyond the hydrogen molecular ion due to the complexity of electron-electron correlation. The Born-Oppenheimer approximation simplifies it by invoking the separation of variables that describe electronic and nuclear motion.

$$H\Psi(r,R) =$$

$$\left(-\frac{\hbar^2}{2}\sum_{k=1}^{K}\frac{1}{M_k}\nabla_k^2 - \frac{\hbar^2}{2m}\sum_{i=1}^{N}\nabla_i^2 + \frac{1}{4\pi\epsilon_0}\sum_{k=1,k>k'}^{K}\frac{Z_k e Z_{k'} e}{R_{kk'}} + \frac{1}{4\pi\epsilon_0}\sum_{i=1,i>i'}^{N}\frac{e^2}{r_{ii'}} - \frac{1}{4\pi\epsilon_0}\sum_{k=1,k>k'}^{K}\frac{Z_k e^2}{r_{ik}}\right)\Psi$$

$$= E\Psi(r,R)$$

H	Hamiltonian
$\Psi(r,R)$	Molecular wavefunction in electronic, r, and nuclear, R, coordinates
r	Electronic spatial coordinate
R	Nuclear spatial coordinate
$\hbar$	Reduced Planck's constant defined by $\hbar = \frac{h}{2\pi} = 1.05 \times 10^{-34}$ J·s
k	Index of summation of nuclei
K	Total number of nuclei
M_k	Mass of the kth nucleus
∇_k^2	Laplacian of the nuclear coordinates of the kth atom
i	Index of summation of electrons
N	Total number of Electrons
m	Mass of an electron
∇_i^2	Laplacian of the electronic coordinates of the ith electron
ϵ_0	Vacuum permittivity ($\epsilon_0 = 8.85 \times 10^{-12}$ F·m^{-1})
Z_k	Nuclear charge of the kth atom
e	Charge of an electron ($e = 1.60 \times 10^{-19}$ C)
$R_{kk'}$	$\lvert R_{k'} - R_k\rvert$ Nuclear interparticle distance
$r_{ii'}$	$\lvert r_{i'} - r_i\rvert$ Electron interparticle distance
r_{ik}	$\lvert R_k - r_i\rvert$ Electron-nuclei interparticle distance

Table S2: **Transition State Theory**[2] assumes that reactants are in thermal equilibrium with the transition state and that motion along the reaction coordinate is one-dimensional and irreversible (*i.e.,* translational motion separated from all other motions where products cannot reform reactants). It takes statistical properties (in the form of partition functions) of the reactive systems into account but ignores microscopic collision theory.

$$k(T) = \frac{Q^{\dagger}}{Q}\frac{k_BT}{h}e^{-E_a/k_BT}$$

$k(T)$	Temperature dependent rate constant
$Q^{\dagger}$	Partition function of the transition state (†)
Q	Partition function of the reactants
E_a	Activation energy
k_B	Boltzmann constant = 1.38×10^{-23} m^2·kg·s^{-2}·K^{-1} (where k_BT = 25 meV at room temperature, 300 K)
T	Temperature
h	Planck's constant = 6.626×10^{-34} m^2·kg·s^{-1} (equivalently, 4.14×10^{-15} eV·s)

Table S3: Difference gradient (**g**) and derivative coupling (**h**) vectors span the branching plane near a conical intersection. These vectors determine the energy gap and coupling, respectively, between two electronic states.[3–5]

$$\mathbf{g} = \frac{\partial(E_1 - E_2)}{\partial R}$$

$$\mathbf{h} = \langle\psi_1|\tfrac{\partial H}{\partial R}|\psi_2\rangle$$

E_n	Energy of state $\|n\rangle$
R	Nuclear coordinate
ψ_n	Molecular wavefunction for state $\|n\rangle$
H	Hamiltonian

Table S4: **Landau-Zener Transition Probability**, (P_{12}), represents the electronic transition probability between two crossing states. It assumes linear potential energy curves for the two intersecting diabatic states, a constant relative velocity between the atoms, and a constant off-diagonal coupling.[6,7]

$$P_{12} = \exp\left[\frac{-2\pi H_{12}^2}{\hbar v \frac{\partial(H_{11}-H_{22})}{\partial R}}\right]$$

H_{12}	Off-diagonal coupling elements $\langle 1\|H\|2\rangle$
H_{11} or H_{22}	E_1 or E_2, the linear potential energies for the diabatic electronic states $\|1\rangle$ and $\|2\rangle$
$\hbar$	Reduced Planck's constant defined by $\hbar = \frac{h}{2\pi} = 1.05 \times 10^{-34}$ J·s
v	Velocity of the nuclear wave packet, $\frac{dR}{dt}$
$\frac{\partial E}{\partial R}$	Gradient of the potential energy surface

Table S5: **Fermi's Golden Rule**[8] is the transition probability per unit time from an initial state to a final state that lies within a continuum of states. The transition rate is proportional to the coupling strength squared and the final density of states. It is derived using first-order time-dependent perturbation theory where the perturbation is assumed to be weak and the system is in its initial state at time, t=0. Although most textbooks will apply the long-time limit of $t \to \infty$, it is well applicable for a short, finite time and even breaks down beyond the Heisenberg time.[9] It has also been shown to energy from discrete final states with non-zero energy spacing.[10]

$$W_{fi} = \frac{2\pi}{\hbar} |\langle \psi_f | T | \psi_i \rangle|^2 \delta(E_f - E_i - \hbar\omega)$$

W_{fi}	Transition probability between an initial state $\|\psi_i\rangle$ and a final state $\|\psi_f\rangle$
$\hbar$	Reduced Planck's constant defined by $\hbar = \frac{h}{2\pi} = 1.05 \times 10^{-34}$ J·s
T	Transition operator, which could be $\mu = e \cdot r$ for the electric dipole transition, $x_i x_j$ for the electric quadrupole transitions, μ_m for the magnetic dipole, etc.
$\delta(E_f - E_i - \hbar\omega)$	A delta function to define energy conservation, such that the photon energy $\hbar\omega$ is equal to the difference in energy of the final and initial states, $E_f - E_i$

Table S6: The **EXAFS Formula**[1,11] is obtained with the use of partial wave scattering theory and appropriate boundary conditions within an average (muffin-tin) potential. A complete derivation is in Appendix A of Ref.[1]

$$\chi(k) = \sum_j S_0^2 N_j \frac{|f_j(k)|}{kR_j^2} \sin[2kR_j + 2\delta_e + \Phi] \exp(-2R_j/\lambda(k)) \exp(-2\sigma_j^2 k^2)$$

$\chi(k)$	EXAFS signal
S_0^2	Amplitude reduction factor
N_j	Number of atoms, where subscript j indicates the index of summation
$f_j(k)$	The backscattering amplitude
k	Photoelectron wavevector
R_j	Interatomic distance
δ_e	Central-atom partial-wave phase shift of the final state
Φ	Phase offset
$\lambda(k)$	Energy-dependent photoelectron mean free path
σ_j	Root-mean square fluctuation in the bond length

Table S7: Spin-orbit Hamiltonian[12]

$$H_{SO} = -\mu \cdot B = \frac{1}{4\pi\epsilon_0}\frac{e^2}{m^2c^2r^3}S \cdot L$$

S	Total spin angular momentum
L	Total orbital angular momentum
H_{SO}	Spin-orbit Hamiltonian
μ	Magnetic moment
B	Magnetic field
ϵ_0	Vacuum permittivity ($\epsilon_0 = 8.85 \times 10^{-12}$ F·m^{-1})
e	Charge of an electron ($e = 1.60 \times 10^{-19}$ C)
m	Mass of an electron ($m = 9.11 \times 10^{-31}$ kg)
c	Speed of light ($c = 2.998{\times}10^8$ m·s^{-1})
r	Electron coordinates

Table S8: $L \cdot S$ Operator[12]

$$L \cdot S = \frac{1}{2}(J^2 - L^2 - S^2) = \frac{\hbar^2}{2}[j(j+1) - l(l+1) - s(s+1)]$$

J	Total angular momentum
L	Total orbital angular momentum
S	Total spin angular momentum
j	Total angular momentum quantum number, defined as $j = l + s$
l	Orbital angular momentum quantum number
s	Spin quantum number

Table S9: Eigenvalues of spin-orbit coupling[12]

$$E_{SO} = \langle H_{SO} \rangle = \frac{1}{8\pi\epsilon_0}\frac{e^2}{m^2c^2}\frac{\hbar^2/2\,[j(j+1) - l(l+1) - 3/4]}{l(l+1/2)(l+1)n^3a^3}$$

ϵ_0	Vacuum permittivity ($\epsilon_0 = 8.85 \times 10^{-12}$ F·m^{-1})
e	Charge of an electron ($e = 1.60 \times 10^{-19}$ C)
m	Mass of an electron ($m = 9.11 \times 10^{-31}$ kg)
c	Speed of light ($c = 2.998{\times}10^8$ m·s^{-1})
$\hbar$	Reduced Planck's constant defined by $\hbar = \frac{h}{2\pi} = 1.05 \times 10^{-34}$ J·s
j	Total angular momentum quantum number
l	Orbital angular momentum quantum number
n	Principal quantum number
a	Bohr radius, $a = 0.529$ Å

Table S10: Ratio of Einstein co-efficients for spontaneous and stimulated emission[8]

$$\frac{A_{21}}{B_{21}} = \frac{8\pi h\nu^3}{c^3}$$

A_{21}	Einstein co-efficient of spontaneous emission
B_{21}	Einstein co-efficient of stimulated emission
h	Planck's constant
ν	Frequency
c	Speed of light ($c = 2.998 \times 10^8$ m·s^{-1})

Table S11: Self-phase modulation[13]

$$\delta\omega(t) = \omega(t) - \omega_0 = -\frac{\omega_0 n_2 x}{2c}\frac{\partial I(t)}{\partial t}$$

$\delta\omega(t)$	Instantaneous change in the frequency at time t
t	Time
$\omega(t)$	Instantaneous frequency at time t
ω_0	Initial frequency
n_2	Nonlinear refractive index which is dependent on the intensity, $I(t)$
x	Spatial coordinate
c	Speed of light ($c = 2.998\times10^8$ m·s^{-1})
$I(t)$	Intensity of the light pulse at time t

Table S12: The Keldysh parameter[14]

$$\gamma = \frac{t_{tu}}{T_0} = \frac{\omega_0}{\omega_t} = \frac{\omega_0\sqrt{2mI_p}}{eE} = \sqrt{\frac{\omega_0^2 2mI_p}{e^2E^2}} = \sqrt{\frac{I_p}{2U_p}}$$

γ	The Keldysh parameter, which provides a measure of the ratio of the tunneling time and the period of the driving frequency, $\gamma = \frac{t_{tu}}{T_0} = \frac{\omega_0}{\omega_t}$
t_{tu}	Tunneling time
T_0	Period of driving laser
ω_0	Angular frequency of the laser field
ω_t	Tunnel ionization frequency given by $\omega_t = \frac{eE}{\sqrt{2m_eI_p}}$
m	Mass of an electron ($m = 9.11 \times 10^{-31}$ kg)
I_p	Ionization potential of the medium
e	Charge of an electron ($e = 1.60 \times 10^{-19}$ C)
E	Field strength of the laser field
U_p	Ponderomotive energy given by $U_p = \frac{(eE_0)^2}{4m\omega_0^2}$, which is defined in Table S16

Table S13: Electron velocity in a time-varying electric field[14,15]

$$v(t) = \frac{dx}{dt} = \int_{t'}^{t} -\frac{e}{m} E_0 \cos(\omega_0 t) dt = -\frac{eE_0}{m\omega_0} \left[\sin(\omega_0 t) - \sin(\omega_0 t') \right]$$

v	Velocity of the electron
m	Mass of an electron ($m = 9.11 \times 10^{-31}$ kg)
E_0	Amplitude of laser field
ω_0	Angular frequency of laser field
t	Time
x	Position of the electron (in one-dimension)
t'	Ionization time

Table S14: Electron position in a time-varying electric field[14,15]

$$x(t) = \int_{t'}^{t} v(t)dt = \frac{eE_0}{m\omega_0^2}\Big[\big[\cos(\omega_0 t) - \cos(\omega_0 t')\big] + \omega_0 \sin(\omega_0 t')(t - t')\Big]$$

v	Velocity of the electron
m	Mass of an electron ($m = 9.11 \times 10^{-31}$ kg)
E_0	Amplitude of laser field
ω_0	Angular frequency of laser field
t	Time
x	Position of the electron (in one-dimension)
t'	Ionization time

Table S15: Energy of photon released upon recombination[14,15]

$$\hbar\omega = I_p + \frac{1}{2}m\left[-\frac{eE_0}{m\omega_0}[\sin(\omega_0 t) - \sin(\omega_0 t')]\right]^2$$

I_p	Ionization potential of the gas
m	Mass of an electron ($m = 9.11 \times 10^{-31}$ kg)
e	Charge of an electron ($e = 1.60 \times 10^{-19}$ C)
E_0	Amplitude of laser field
ω_0	Angular frequency of laser field
t	Time
t'	Ionization time

Table S16: Cutoff and ponderomotive energy[14,15]

$$\hbar\omega_{max} = I_p + 3.17U_p$$

$$U_p = \frac{e^2 I}{2\epsilon_0 c m \omega_0^2} = \frac{e^2}{8\pi^2\epsilon_0 c^3 m} I\lambda^2$$

$\hbar$	Reduced Planck's constant
ω_{max}	Maximum achievable photon frequency defined in the semiclassical model
I_p	Ionization potential of the gas
U_p	Ponderomotive energy, which describes the cycle-averaged kinetic energy of an electron in an oscillating electric field
e	Charge of an electron ($e = 1.60 \times 10^{-19}$ C)
I	Intensity of the laser pulse
ϵ_0	Vacuum permittivity ($\epsilon_0 = 8.85 \times 10^{-12}$ F·m^{-1})
c	Speed of light ($c = 2.998\times10^8$ m·s^{-1})
m	Mass of an electron ($m = 9.11 \times 10^{-31}$)
ω_0	Angular frequency of the fundamental field
λ	Wavelength of the fundamental field

Table S17: Periodicity in the harmonic spectrum[13]

$$E_{\omega_q} = A_{\omega_q} \exp\left[-i(\omega_q t + \phi_q)\right] \sum_n \exp\left[-i\left(\frac{\omega_q n T_0}{2} - n\pi\right)\right]$$

ω_q	Frequency of arbitrary harmonic, q
E_{ω_q}	Electric field of arbitrary harmonic frequency ω_q
A_{ω_q}	Amplitude of the electric field for arbitrary frequency, ω_q
t	Time
n	Index of summation
T_0	Period of the driving laser field
ϕ_q	Phase of E_{ω_q}

Table S18: Wavevector of a laser field in a gaseous medium[16]

$$k = \frac{2\pi}{\lambda} + \frac{2\pi N_a n(\lambda)}{\lambda} - N_e r_e \lambda$$

k	Wavevector
λ	Wavelength
N_a	Number density of neutral atoms
$n(\lambda)$	Linear refractive index per neutral atom density at wavelength λ
N_e	Number density of free-electrons
r_e	Classical electron radius, $r_e = 2.818\times10^{-15}$ m

Table S19: Phase matching for the qth harmonic order[17]

$$\Delta k_q = \frac{2\pi q}{\lambda}(1-\eta)P\Delta n - P\eta N_{atm} r_e \lambda \left[\frac{q^2-1}{q}\right]$$

Δk_q	Phase matching parameter, which describes the difference in wavevectors k of the driving wave of the fundamental frequency and the generated wave at the qth harmonic order
λ	Wavelength of the fundamental frequency
q	Harmonic order
η	Ionization fraction
P	Gas pressure
Δn	Difference in the refractive indices of the neutral gas at atmospheric pressure $\Delta n = n_{laser}^{atm} - n_{x-ray}^{atm}$
N_{atm}	Atom density at atmospheric pressure

Table S20: Gouy phase[18–20]

$$\Delta\phi_{Gouy} = -\arctan\left(\frac{z}{z_R}\right)$$

$\Delta\phi_{Gouy}$	The Gouy phase, which describes the phase shift of a Gaussian beam through its focus
z	Spatial coordinate in the direction of laser propagation
z_r	Rayleigh length of the focus

Table S21: Free-standing thickness-to-diameter ratio[21]

$$\frac{t}{D} = \sqrt{\frac{KpS_f}{4Y}}$$

t	Material thickness
D	Free-standing diameter of material
K	Proportionality constant, which varies between 0.75 and 1.125 for a clamped or free edge, respectively.
p	Pressure differential
S_f	Safety factor, where $S_f > 4$ is considered adequate
Y	Flexural strength

Table S22: Grating equation

$$m\lambda = d(\sin\alpha - \sin\beta)$$

m	Integer value of diffraction order
λ	Wavelength
d	Groove spacing
α	Incidence angle
β	Diffraction angle

References

(1) Stöhr, J. *NEXAFS Spectroscopy*; Springer Berlin Heidelberg, 1992; Vol. 25.

(2) Steinfeld, J. I.; Francisco, J. S.; Hase, W. L. *Chemical kinetics and dynamics*; Prentice Hall Upper Saddle River, NJ, 1999; Vol. 2.

(3) Yarkony, D. R. Conical intersections: Diabolical and often misunderstood. *Accounts of Chemical Research* **1998**, *31*, 511–518.

(4) Lee, A.; Coe, J.; Ullrich, S.; Ho, M.-L.; Lee, S.-J.; Cheng, B.-M.; Zgierski, M.; Chen, I.; Martinez, T.; Stolow, A. Substituent effects on dynamics at conical intersections: α, β-enones. *J. Phys. Chem. A* **2007**, *111*, 11948–11960.

(5) Domcke, W.; Yarkony, D. *Conical intersections: theory, computation and experiment*; World Scientific, 2011; Vol. 17.

(6) Tully, J. C. Perspective: Nonadiabatic dynamics theory. *J. Chem. Phys.* **2012**, *137*, 22A301.

(7) Nelson, T. R.; White, A. J.; Bjorgaard, J. A.; Sifain, A. E.; Zhang, Y.; Nebgen, B.; Fernandez-Alberti, S.; Mozyrsky, D.; Roitberg, A. E.; Tretiak, S. Non-adiabatic excited-state molecular dynamics: Theory and applications for modeling photophysics in extended molecular materials. *Chemical Reviews* **2020**, *120*, 2215–2287.

(8) McHale, J. L. *Molecular spectroscopy*; CRC Press, 2017.

(9) Zhang, J.-M.; Liu, Y. Fermi's golden rule: its derivation and breakdown by an ideal model. *European Journal of Physics* **2016**, *37*, 065406.

(10) Micklitz, T.; Morningstar, A.; Altland, A.; Huse, D. A. Emergence of fermi's golden rule. *Phys. Rev. Lett.* **2022**, *129*, 140402.

(11) Bressler, C.; Chergui, M. Ultrafast x-ray absorption spectroscopy. *Chemical Reviews* **2004**, *104*, 1781–1812.

(12) Griffiths, D. J.; Schroeter, D. F. *Introduction to quantum mechanics*; Cambridge university press, 2018.

(13) Rulliere, C. *Femtosecond Laser Pulses: Principles and Experiments*, 2nd ed.; Springer, 2003.

(14) Chang, Z. *Fundamentals of attosecond optics*; CRC press, 2016.

(15) Corkum, P. B. Plasma perspective on strong field multiphoton ionization. *Phys. Rev. Lett.* **1993**, *71*, 1994–1997.

(16) Rundquist, A.; Durfee, C. G.; Chang, Z.; Herne, C.; Backus, S.; Murnane, M. M.; Kapteyn, H. C. Phase-Matched Generation of Coherent Soft X-rays. *Science* **1998**, *280*, 1412–1415.

(17) Durfee, C. G.; Rundquist, A. R.; Backus, S.; Herne, C.; Murnane, M. M.; Kapteyn, H. C. Phase Matching of High-Order Harmonics in Hollow Waveguides. *Phys. Rev. Lett.* **1999**, *83*, 2187–2190.

(18) Feng, S.; Winful, H. G. Physical origin of the Gouy phase shift. *Opt. Lett.* **2001**, *26*, 485–487.

(19) Lindner, F.; Paulus, G. G.; Walther, H.; Baltuška, A.; Goulielmakis, E.; Lezius, M.; Krausz, F. Gouy Phase Shift for Few-Cycle Laser Pulses. *Phys. Rev. Lett.* **2004**, *92*, 113001.

(20) Li, X. F.; L'Huillier, A.; Ferray, M.; Lompré, L. A.; Mainfray, G. Multiple-harmonic generation in rare gases at high laser intensity. *Physical Review A* **1989**, *39*, 5751–5761.

(21) Moore, J. H.; Davis, C. C.; Coplan, M. A. *Building scientific apparatus : a practical guide to design and construction*; Addison-Wesley Publishing Company, 1983.
